# Design, Synthesis, and Mechanism of Dihydroartemisinin–Coumarin Hybrids as Potential Anti-Neuroinflammatory Agents

**DOI:** 10.3390/molecules24091672

**Published:** 2019-04-28

**Authors:** Haonan Yu, Zhuang Hou, Xiaoguang Yang, Yanhua Mou, Chun Guo

**Affiliations:** 1 School of Pharmaceutical Engineering, Shenyang Pharmaceutical University, Shenyang 110016, China; yhna380@hotmail.com (H.Y.); houzhuang8@sina.com (Z.H.); Xiaog_yang@163.com (X.Y.); 2 School of Life Sciences and Biological Pharmacy, Shenyang Pharmaceutical University, Shenyang 110016, China

**Keywords:** anticancer, anti-neuroinflammation, coumarin, dihydroartemisinin

## Abstract

Cancer patients frequently suffer from cancer-related fatigue (CRF), which is a complex syndrome associated with weakness and depressed mood. Neuroinflammation is one of the major inducers of CRF. The aim of this study is to find a potential agent not only on the treatment of cancer, but also for reducing CRF level of cancer patients. In this study, total-thirty new Dihydroartemisinin–Coumarin hybrids (DCH) were designed and synthesized. The in vitro cytotoxicity against cancer cell lines (HT-29, MDA-MB-231, HCT-116, and A549) was evaluated. Simultaneously, we also tested the anti-neuroinflammatory activity of DCH. DCH could inhibit the activated microglia N9 release of NO, TNF-α, and IL-6. The docking analysis was shown that MD-2, the coreceptor of TLR4, might be one of the targets of DCH.

## 1. Introduction

Chemotherapy is an important method in the therapy of cancer, but the adverse side effects of anticancer agents should also be focused on. Gastrointestinal injury induced by chemotherapeutic agents could result in bacterial/endotoxin translocation from the gut to the systemic blood circulation [1,2]. The released endotoxin would cause a systemic inflammatory response including neuroinflammation [3]. Cancer patients frequently suffer from cancer-related fatigue (CRF), which is a complex syndrome associated with weakness and depressed mood. Diana M., Norden et al. demonstrated that the neuroinflammation is one of the major reasons which induce CRF [4]. Currently, there were no effective method to reduce CRF [5]. The aim of this study is to find a potential anti-neuroinflammatory agents which might reduce CRF of cancer patients.

Central nervous system (CNS) consists of two major types of cells, nerve cells and glial cells. Microglia is a major glial cell and plays an important role as the resident macrophage population in the CNS [6,7]. There has been wide recognition that the fully activated microglial cells are neurotoxic. They release reactive oxygen species, nitric oxide (NO), tumor necrosis factor-alpha (TNF-α), and other potentially cytotoxic molecules [8,9]. It means that activation of microglia could induce neuroinflammation.

Lipopolysaccharide (LPS), also known as endotoxin, is a natural Toll-like receptor 4 (TLR4) ligand and it is also a potent microglial activator that can trigger the production of inflammatory mediators. TLR-4, a transmembrane receptor, plays a vital role in the initiation and acceleration of the inflammatory response induced by LPS [10,11]. TLR4 alone does not directly bind LPS and needs the coreceptor MD-2 [12]. MD-2 is linked with the extracellular domain of TLR4 and is indispensable for LPS recognition [13]. MD-2 is a member of the MD-2-related lipid-recognition protein family [14]. MD-2 directly binds to LPS in its hydrophobic cavity with high affinity [15].

Artemisinin and its derivatives have been the first-line antimalarial drug since the late 1990s. Dihydroartemisinin (DHA) is the primary bioactive metabolite of artemisinin and its derivatives, but not artemisinin per se [16]. Additionally, DHA has higher bioavailability and less side effects than artemisinin [17]. Recent research showed that besides antimalarial activity, DHA also exhibits anticancer and anti-inflammatory activity [18,19,20,21]. As the anti-inflammatory agent, DHA could decrease LPS-induced protein expression of TLR4 [22].

Coumarins are widely distributed in different parts of plants. There are various pharmacological activities of coumarins, which includes anti-inflammatory [23,24] and anticancer activity [25,26]. As the anticancer agents, coumarins were reported to exhibit negligible or mild side effects [27,28]. Substitution at the C-3 or C-4 position of coumarins exhibit good cytotoxic activity in various cancer cell lines [26,29,30,31]. Additionally, as anti-inflammatory agents, some kinds of coumarins could also target TLR4/MD-2 complex [32,33].

In our previous study, Dihydroartemisinin–Coumarin hybrids (DCH) were designed and synthesized referring to the principle of hybridization [34,35]. Each of them contains coumarin moiety, DHA moiety and a linker. DCH could inhibit HT-29 cells proliferation and arrest the G_0_/G_1_ phase of HT-29 cells [35]. They could also suppress cancer cell migration, and induce apoptosis and ferroptosis of HT-29 cancer cells [35]. It also has been confirmed that the DCH having 1,2,3-triazole linker generally exhibit better cytotoxicity exhibition than the others [35].

**Figure 1:**
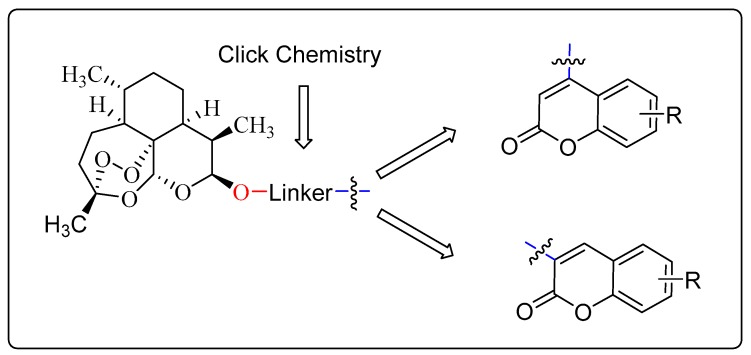
Structures of new designed Dihydroartemisinin–Coumarin hybrids.

With this background, total-thirty new DCH were designed and synthesized, and the synthetic approaches were shown in Figure 1. All of them contained 1,2,3-triazole linker. We focused on the modifications of positions 3 and 4 of the coumarin moiety. Due to the new synthesized DCH were for the cancer patients, we evaluated the in vitro cytotoxicity against cancer cell lines (HT-29, MDA-MB-231, HCT-116, and A549). In addition, DCH could inhibit the activated N9 cell release of NO, TNF-α and IL-6. Furthermore, to investigate the mechanism of the anti-neuroinflammatory activity, docking analysis was applied. The results might support that the anti-neuroinflammatory activity was related to inhibiting TLR4/MD-2 complex.

## 2. Results and Discussion

### 2.1. Chemistry

**Scheme 1:**
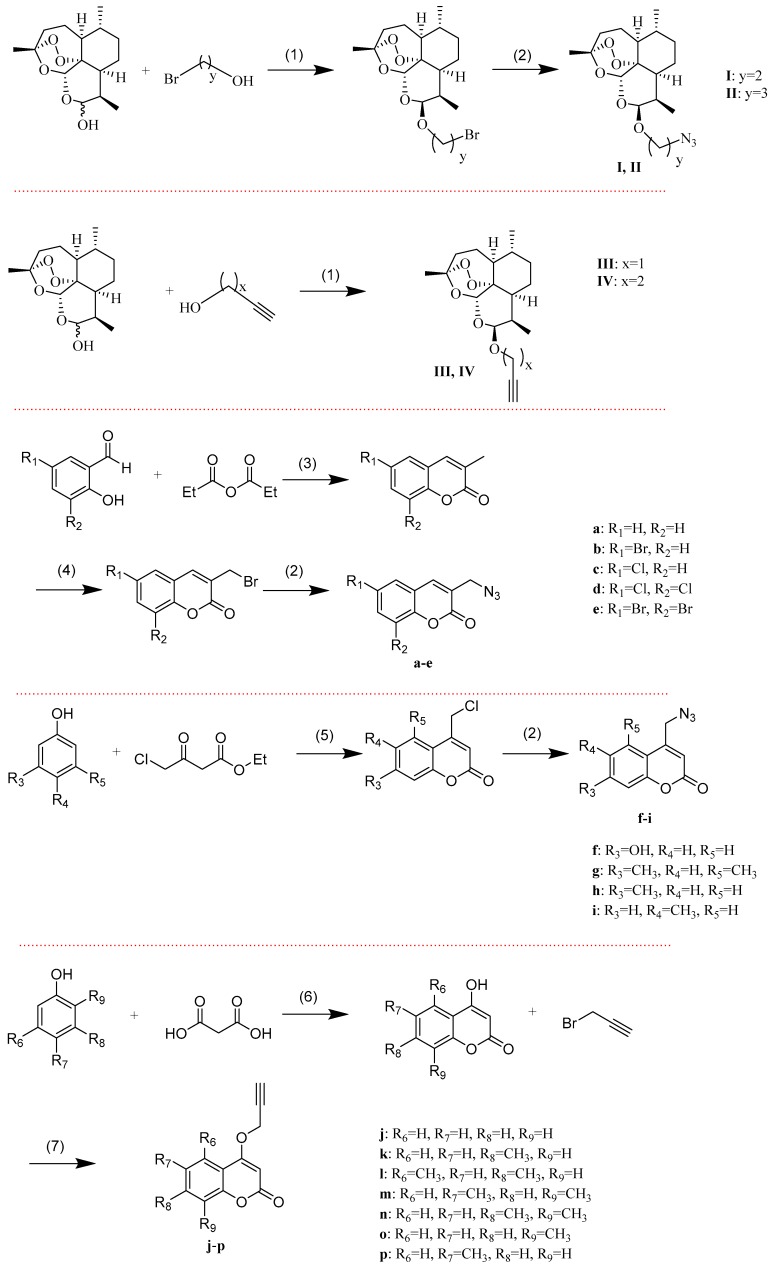
Synthetic routes of intermediates. Reagents and conditions: (1) BF_3_^.^Et_2_O, CH_2_Cl_2_, 0 °C; (2) NaN_3_, DMF, 60 °C; (3) CH_3_CH_2_COONa, Et_3_N, reflux; (4) NBS, BPO, CCl_4_, reflux; (5) 70% H_2_SO_4_, r.t.; (6) ZnCl_2_, POCl_3_, 65 °C; and (7) K_2_CO_3_, DMF, 60 °C.

The synthesis of the intermediates **a**–**p** and **I**–**IV** is presented in Scheme 1. The intermediates **I** and **II** were obtained by 2-bromoethanol or 3-bromo-1-propanol, with dihydroartemisinin (DHA), respectively, followed by treating the product with sodium azide. The intermediates **III** and **IV** were synthesized by propargyl alcohol or 3-butyn-1-ol with DHA, respectively. The required 3-methylcoumarin derivatives were obtained by substitution of salicylaldehyde with propionic anhydride in the presence of sodium propionate and triethylamine. The substituted-3-bromomethylcoumarins were prepared by the radical bromination of 3-methylcoumarin derivatives through the reaction with N-Bromosuccinimide (NBS) in the presence of dibenzoylperoxide [36]. The intermediates **a**–**e** were synthesized by substituted-3-bromomethylcoumarins via azidation reaction. The required substituted-4-chloromethylcoumarins were prepared by the Pechmann cyclisation [37] of substituted phenols with 4-chloroethylacetoacetate using sulfuric acid as the condensing agent. The intermediates **f**–**i** were synthesized by substituted-4-chloromethylcoumarin via azidation reaction. To synthesize the intermediates **j**–**p**, several substituted-4-hydroxycoumarin were prepared by condensing the substituted phenol with malonic acid [38]. Then the products were treated with 3-bromo-1-propyne in the presence of potassium carbonate.

**Scheme 2:**
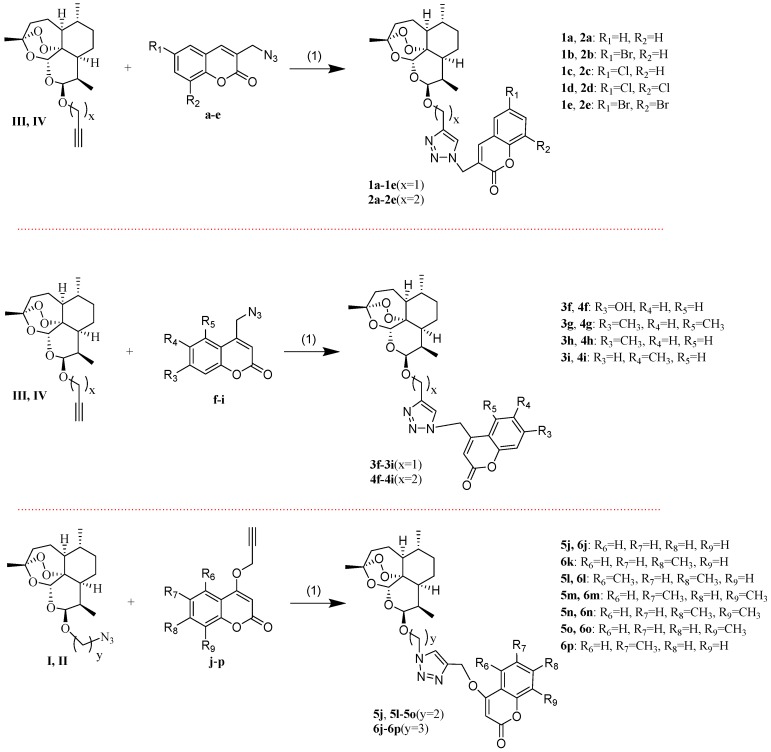
Synthetic routes of target compounds. Reagents and conditions: (1) CuI, triethylamine, dichloromethane, r.t.

The synthesis of the desired new compounds was presented in Scheme 2. The target compounds **1a**–**1e** and **2a**–**2e** were obtained by the reaction of the intermediates **III** or **IV** with **a**–**e** via click chemistry, respectively. The target compounds **3f**–**3i** and **4f**–**4i** were synthesized by intermediates **III** or **IV** with **f**–**i** via click chemistry, respectively. The target compounds **5j**, **5l**–**5o**, and **6j**–**6p** were prepared by the reaction of the intermediates **I** or **II** with **j**–**p** via click chemistry, respectively.

### 2.2. Biological Assay

#### 2.2.1. In Vitro Cytotoxic Activity of the Compounds

As the anti-neuroinflammatory agents for cancer patients, all newly synthesized compounds were evaluated in vitro for antitumor activity against human breast cancer cell line MDA-MB-231, colorectal cancer cells HT-29 and HCT-116, and lung cancer cell line A549 by using the standard MTT method, respectively. Doxorubicin (DOX) and DHA were used as the reference standard. Additionally, owing to the rapid proliferation, the tumor cells actually was always in hypoxia condition. Therefore, the in vitro cytotoxicity was evaluated in two different conditions (hypoxia and normoxia). All data are summarized in Table 1. Most of the target compounds exhibited moderate activities against the tested cell lines and they showed a general promotion under hypoxia conditions.

The structure–activity relationship was evaluated: (i) the series of compounds **5** and **6** generally exhibited better cytotoxicity activity than that of the series of compounds **3** and **4**, which indicated that the 4-oxygen group in the coumarin moiety as a part of linker could improve the antitumor activity; (ii) although compounds **3** and **4** did not exhibit good activity against three cell lines (MDA-MB-231, HCT-116, and A549), they showed good activity against HT-29 like the other derivatives. Therefore, 4-methyl group in the coumarin moiety could improve the selectivity of DCH against HT-29.

In addition, almost all of the target compounds exhibited good cytotoxicity activity against HT-29. Therefore, HT-29 was selected for the next explorations.

#### 2.2.2. Morphological Changes and Effect of Compound 5n on Cell Viability

**Figure 2:**
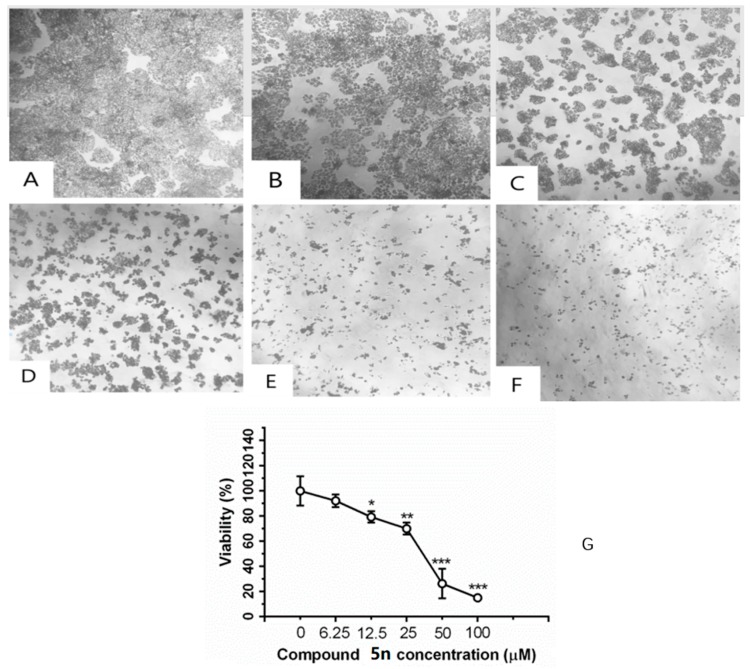
Inhibition of compound **5n** on cell viability in HT-29 cells by MTT staining. Cells were treated with (**A**) 0 μM, (**B**) 6.25 μM, (**C**) 12.5 μM, (**D**) 25.0 μM, (**E**) 50.0 μM, and (**F**) 100.0 μM compound **5n** for 48 h. Representative images were shown; 200×. (**G**) Cells were treated with 6.25, 12.5, 25, 50, and 100 μM compound **5n** for 48 h. Data were shown as mean ± SD. * *p* < 0.05, ** *p* < 0.01 and *** *p* < 0.001 compared with group 0 μM.

As shown in A–F of Figure 2, there showed a concentration-dependent cell death after treatment with compound **5n** for 48 h. As the concentration up to 100 μM, the compound **5n** could obviously inhibit the proliferation of HT-29. As shown in the (G) of Figure 2, after HT-29 cells were treated with different concentrations of **5n** for 48 h, the cell viability decreased in a dose-dependent manner.

#### 2.2.3. Anti-Neuroinflammatory Activity

The anti-neuroinflammatory activity of DCH was evaluated and then the representative compounds were selected for the further study. As shown in Table 2, murine microglial N9 cells were treated with 1μg/mL LPS for 48 h and the NO level in the medium was measured as the preliminary screening. Most of the tested compounds exhibited good anti-inflammatory activity, and especially the IC_50_ of some compounds were even less than 1 μM. Among them, compound **5n** had the best activity and the IC_50_ reached 0.22 μM. In addition, in order to exclude the death influence, MTT-IC20 of all the compounds were measured. The results showed that DCH did not exhibit cytotoxic activity in N9 cells. The exact reason was still unclear, but we supposed that it might be related to the homeostasis of cells. The tumor cells actually were always in hypoxia condition, in which DCH could promote the cytotoxic activity against tumor cells. In brief, DCH could reduce the release of NO caused by LPS-induced microglial cells.

##### NO Capture Capability

**Figure 3:**
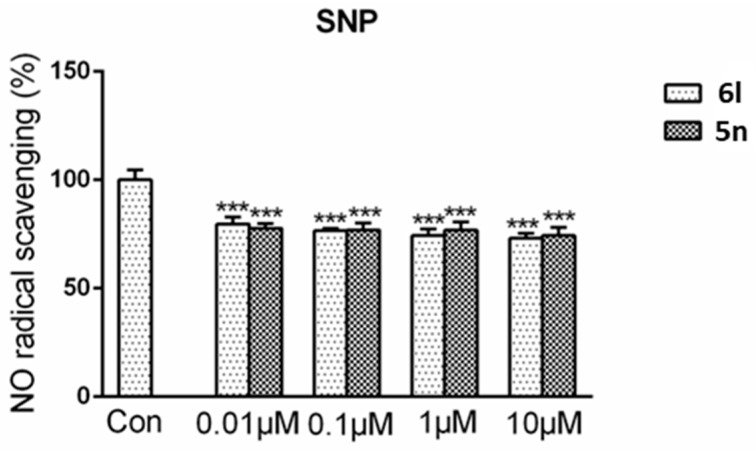
Compound 6l and 5n had NO radical scavenging activity. 6l (0.01 μM, 0.1 μM, 1 μM, and 10 μM) or **5n** (0.01 μM, 0.1 μM, 1 μM, and 10 μM) was added to a concentration of 0.5 mM SNP solution. After reacted for 1 h under normal sun light at room temperature, Griess reagent was added and the optical density was observed at 540 nm. Data were shown as mean ± SD. *** *p* < 0.001 compared with control group.

Sodium nitroprusside (SNP) is a NO donor. In this experiment, the reason of the DCH reducing NO level was preliminary explored. As shown in Figure 3, the results showed that the compounds only partially reduced NO levels by direct NO capture. That means the main reason of the NO level reduction is due to species-specific effect.

##### Effect of Representative Compounds on TNF-α and IL-6 Release

As shown in Table 3, four kinds of DCH were involved to measure the effect on the release of TNF-α and IL-6 from the microglia cells which induced by LPS for 48 h. The results showed that compounds **2b**, **2c**, **5n**, and **6p** could inhibit the release of TNF-α with the inhibition rate of 22% to 32% and the release of IL-6 with the inhibition rate of 26% to 43%. The results indicated that the inhibition effect of DCH on NO release was not only by nonspecific effects, but also by slightly influencing the synthesis and release of upstream inflammatory pathway.

##### Docking Analysis

**Figure 4:**
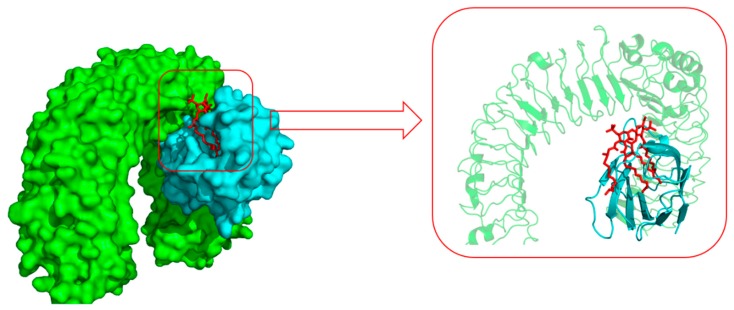
The surface representation of LPS (red) and Toll-like receptor 4 (TLR4) (green)/MD-2 (cyan) binding pocket (PDB: 3VQ2).

**Figure 5:**
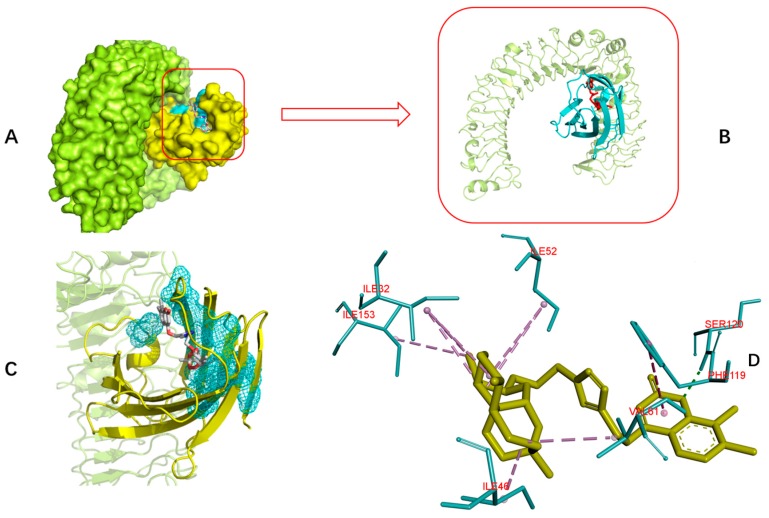
Interaction diagrams of the selected docked conformations of compound 5n to TLR4/MD-2 complex. (**A**) The surface representation of compound **5n** and TLR4 (limon)/MD-2 (yellow) binding pocket. (**B**) The magnified representation of compound **5n** (red) and TLR4 (limon)/MD-2 (cyan) binding pocket. (**C**) The interface of compound **5n** and TLR4/MD-2 complex; (**D**) 3D ligand interactions diagram. Dotted line; pink: alkyl hydrophobic; blue: pi hydrophobic; green: hydrogen bond.

Toll-like receptor 4 (TLR4) and its coreceptor—MD-2—are the target of LPS, which could influence innate immune response to release potentially cytotoxic molecules. MD-2 is linked with the extracellular domain of TLR4 and is indispensable for LPS recognition [13]. Therefore, this docking analysis was applied to explore whether or not the target compounds inhibit MD-2 to reduce the release of inflammatory mediators. Umeharu Ohto et al. [39] had revealed the crystal structure of mouse TLR4/MD-2/LPS complex, which was shown in Figure 4. In their study, MD-2 directly bound to LPS in its hydrophobic cavity with high affinity. With this background, compound **5n** was used as the ligand to explore the relationship between DCH and TLR4/MD-2/LPS complex. As shown in Figure 5, compound **5n** could integrate closely with the hydrophobic cavity of MD-2, which is the important TLR4/MD-2/LPS binding position. That means the DCH might disturb the interactions between LPS and TLR4/MD-2 complex. The minimum binding energy of (ΔG) is −10.08 kcal/mol. Given to this result, we supposed TLR4/MD-2 complex is likely to be one of the drug targets of DCH.

## 3. Conclusions

Total-thirty new DCH were designed and synthesized. Most of the target compounds exhibited moderate activities against the tested cell lines and they showed a general promotion under hypoxia conditions. Meanwhile, the new potential activity of DCH was found that DCH could exhibit anti-neuroinflammatory activity. DCH could inhibit the release of NO, TNF-α, and IL-6, which were induced by LPS. The docking analysis was shown that MD-2, the coreceptor of TLR4, might be one of the targets of DCH. Additionally, DCH could also reduce NO levels by direct NO capture. In brief, DCH were the potential anti-neuroinflammatory agents which might reduce CRF of cancer patients.

## 4. Experimental Section

### 4.1. Chemistry

Unless otherwise specified, all solvents and reagents were commercially available and used without further purification. Analytical TLC was performed using silica gel precoated GF254 plates. The ^1^H-NMR and ^13^C-NMR spectra were recorded at 600 MHz and 150 MHZ on a Bruker AV-400 spectrometer (Bruker Bioscience, Billerica, MA, USA), using CDCl_3_ as solvent with tetramethylsilane as the internal standard. NMR spectra were analyzed using MestReNova. High-resolution mass spectra (HRMS) were measured using an Agilent Accurate-Mass Q-TOF 6530 (Agilent, Santa Clara, CA, USA) instrument in ESI mode. The ^1^H-NMR and ^13^C-NMR spectra of synthesized compounds, please see the supplementary materials.

#### 4.1.1. General Procedure for the Synthesis of Compounds **1a**–**1e** and **2a**–**2e**

Substituted coumarin (**a**–**e**) (10 mmol), substituted DHA (**III** or **IV**) (10 mmol), and triethylamine (10 mmol) were dissolved in 20 mL CH_2_Cl_2_. In addition, CuI (100 mg) was added and the reaction mixture was stirred at room temperature for 8 h under the protection of nitrogen. The mixture was filtered, washed with water, dried over Na_2_SO_4_ and evaporated to dryness. The crude product was purified through column chromatography.

*3-((4-((10S)-dihydroartemisininoxy)methyl-1H-1,2,3-triazol-1-yl)-methyl)-2H-1-chromen-2-one* (**1a**) White solid; m.p. 73–75 °C; yield 52%; ^1^H NMR (600 MHz, CDCl_3_) δ (ppm): 7.77 (s, 1H), 7.68 (s, 1H), 7.56 (t, *J* = 7.9 Hz, 1H), 7.47 (d, *J* = 7.7 Hz, 1H), 7.34 (d, *J* = 8.4 Hz, 1H), 7.30 (t, *J* = 7.6 Hz, 1H), 5.47 (s, 2H), 5.39 (s, 1H), 4.94 (d, *J* = 12.6 Hz, 1H), 4.92 (d, *J* = 3.5 Hz, 1H), 4.68 (d, *J* = 12.6 Hz, 1H), 2.67–2.60 (m, 1H), 2.38–2.31 (m, 1H), 2.01–1.97 (m, 1H), 1.85–1.81 (m, 1H), 1.77–1.68 (m, 3H), 1.59 (dd, *J* = 13.2, 3.4 Hz, 1H), 1.46–1.43 (m, 1H), 1.41 (s, 3H), 1.31–1.18 (m, 1H), 1.26–1.16 (m, 2H), 0.91 (d, *J* = 6.3 Hz, 3H), 0.88 (d, *J* = 7.3 Hz, 3H); ^13^C NMR (150 MHz, CDCl_3_) δ (ppm): 159.64, 152.65, 144.62, 141.31, 131.46, 127.30, 123.92, 122.67, 121.84, 117.53, 115.71, 103.10, 100.77, 86.93, 80.06, 76.22, 76.01, 75.80, 60.70, 51.47, 47.97, 43.33, 36.33, 35.36, 33.52, 29.78, 25.09, 23.59, 23.41, 19.30, 11.99; ESI-HRMS [M + Na]^+^: (*m*/*z*) Calcd. for C_28_H_33_N_3_O_7_Na: 546.2216. Found: 546.2181.

*6-bromo-3-((4-((10S)-dihydroartemisininoxy)methyl-1H-1,2,3-triazol-1-yl)-methyl)-2H-1-chromen-2-one* (**1b**) White solid; m.p. 78–80 °C; yield 48%; ^1^H NMR (600 MHz, CDCl_3_) δ (ppm): 7.75 (s, 1H), 7.64 (dd, *J* = 8.8, 2.3 Hz, 1H), 7.61 (d, *J* = 2.3 Hz, 1H), 7.55 (s, 1H), 7.24 (d, *J* = 8.8 Hz, 1H), 5.47 (s, 2H), 5.39 (s, 1H), 4.94 (d, *J* = 12.7 Hz, 1H), 4.93 (d, *J* = 3.5 Hz, 1H), 4.69 (d, *J* = 12.8 Hz, 1H), 2.67–2.63 (m, 1H), 2.38–2.33 (m, 1H), 2.03–1.99 (m, 1H), 1.86–1.82 (m, 1H), 1.75–1.70 (m, 2H), 1.62–1.58 (m, 2H), 1.42 (s, 3H), 1.31–1.19 (m, 4H), 0.92 (d, *J* = 6.3 Hz, 3H), 0.89 (d, *J* = 7.4 Hz, 3H); ^13^C NMR (150 MHz, CDCl_3_) *δ* (ppm): 158.90, 151.42, 144.74, 139.67, 134.18, 129.51, 123.17, 122.67, 119.00, 117.43, 116.53, 103.12, 100.87, 86.95, 80.06, 76.20, 75.99, 75.78, 60.76, 51.46, 47.82, 43.32, 36.36, 35.36, 33.52, 29.79, 25.10, 23.62, 23.42, 19.30, 12.00; ESI-HRMS [M + Na]^+^: (*m*/*z*) Calcd. for C_28_H_32_BrN_3_O_7_Na: 624.1321. Found: 624.1264.

*6-chloro-3-((4-((10S)-dihydroartemisininoxy)methyl-1H-1,2,3-triazol-1-yl)-methyl)-2H-1-chromen-2-one* (**1c**) White solid; m.p. 93–95 °C; yield 41%; ^1^H NMR (600 MHz, CDCl_3_) δ (ppm): 7.75 (s, 1H), 7.56 (s, 1H), 7.51 (dd, *J* = 8.8, 2.5 Hz, 1H), 7.45 (d, *J* = 2.4 Hz, 1H), 7.30 (d, *J* = 8.9 Hz, 1H), 5.47 (s, 2H), 5.39 (s, 1H), 4.95 (d, *J* = 12.7 Hz, 1H), 4.93 (d, *J* = 3.6 Hz, 1H), 4.69 (d, *J* = 12.6 Hz, 1H), 2.68–2.62 (m, 1H), 2.35 (td, *J* = 14.0, 4.0 Hz, 1H), 2.03–1.99 (m, 1H), 1.87–1.82 (m, 1H), 1.75–1.71 (m, 2H), 1.62–1.59 (m, 1H), 1.48–1.43 (m, 2H), 1.42 (s, 3H), 1.31–1.19 (m, 3H), 0.92 (d, *J* = 6.3 Hz, 3H), 0.89 (d, *J* = 7.4 Hz, 3H); ^13^C NMR (150 MHz, CDCl_3_) *δ* (ppm): 158.96, 150.95, 144.73, 139.79, 131.37, 129.27, 126.46, 123.19, 122.67, 118.51, 117.16, 103.12, 100.86, 86.95, 80.06, 76.21, 75.99, 75.78, 60.75, 51.47, 47.84, 43.33, 36.36, 35.36, 33.52, 29.79, 25.10, 23.62, 23.42, 19.30, 12.00; ESI-HRMS [M + Na]^+^: (*m*/*z*) Calcd. for C_28_H_32_ClN_3_O_7_Na: 580.1826. Found: 580.1788.

*6,8-dichloro-3-((4-((10S)-dihydroartemisininoxy)methyl-1H-1,2,3-triazol-1-yl)-methyl)-2H-1-chromen-2-one* (**1d**) White solid; m.p. 113–115 °C; yield 60%; ^1^H NMR (600 MHz, CDCl_3_) δ (ppm): 7.75 (s, 1H), 7.61 (d, *J* = 2.3 Hz, 1H), 7.55 (s, 1H), 7.37 (d, *J* = 2.4 Hz, 1H), 5.48 (s, 2H), 5.37 (s, 1H), 4.96–4.92 (m, 2H), 4.70 (d, *J* = 12.7 Hz, 1H), 2.69–2.62 (m, 1H), 2.38–2.33 (m, 1H), 2.04–1.99 (m, 1H), 1.87–1.82 (m, 1H), 1.76–1.72 (m, 3H), 1.62–1.59 (m, 1H), 1.42 (s, 3H), 1.32–1.18 (m, 4H), 0.92 (d, *J* = 6.3 Hz, 3H), 0.89 (d, *J* = 7.3 Hz, 3H); ^13^C NMR (150 MHz, CDCl_3_) *δ* (ppm): 157.92, 146.92, 144.86, 139.45, 131.36, 129.12, 125.03, 124.04, 122.68, 121.71, 119.30, 103.13, 100.95, 86.94, 80.04, 76.20, 75.99, 75.78, 60.80, 51.44, 47.63, 43.31, 36.37, 35.35, 33.51, 29.79, 25.09, 23.61, 23.42, 19.28, 12.00; ESI-HRMS [M + Na]^+^: (*m*/*z*) Calcd. for C_28_H_31_Cl_2_N_3_O_7_Na: 614.1437. Found: 614.1428.

*6,8-dibromo-3-((4-((10S)-dihydroartemisininoxy)methyl-1H-1,2,3-triazol-1-yl)-methyl)-2H-1-chromen-2-one* (**1e**) White solid; m.p. 103–105 °C; yield 61%; ^1^H NMR (600 MHz, CDCl_3_) δ (ppm): 7.90 (d, *J* = 2.2 Hz, 1H), 7.75 (s, 1H), 7.56 (d, *J* = 2.2 Hz, 1H), 7.52 (s, 1H), 5.48 (s, 2H), 5.37 (s, 1H), 4.96–4.92 (m, 2H), 4.70 (d, *J* = 12.7 Hz, 1H), 2.67–2.63 (m, 1H), 2.38–2.33 (m, 1H), 2.03–1.99 (m, 1H), 1.87–1.82 (m, 1H), 1.76–1.72 (m, 2H), 1.62–1.58 (m, 4H), 1.42 (s, 3H), 1.25–1.19 (m, 2H), 0.92 (d, *J* = 6.3 Hz, 3H), 0.90 (d, *J* = 7.4 Hz, 3H); ^13^C NMR (150 MHz, CDCl_3_) *δ* (ppm): 158.01, 148.45, 144.87, 139.36, 136.89, 128.75, 123.97, 122.67, 119.74, 116.48, 110.29, 103.13, 100.97, 86.95, 80.04, 76.20, 75.99, 75.78, 60.82, 51.45, 47.56, 43.31, 36.37, 35.35, 33.51, 29.79, 25.09, 23.62, 23.42, 19.30, 12.00; ESI-HRMS [M + Na]^+^: (*m*/*z*) Calcd. for C_28_H_31_Br_2_N_3_O_7_Na: 702.0426. Found: 702.0421.

*3-((4-(2-((10S)-dihydroartemisininoxy)ethyl)-1H-1,2,3-triazol-1-yl)-methyl)-2H-1-chromen-2-one* (**2a**) White solid; m.p. 143–145 °C; yield 66%; ^1^H NMR (600 MHz, CDCl_3_) δ (ppm): 7.65 (s, 1H), 7.63 (s, 1H), 7.56 (ddd, *J* = 8.7, 7.3, 1.6 Hz, 1H), 7.47 (dd, *J* = 7.8, 1.6 Hz, 1H), 7.35 (d, *J* = 8.4 Hz, 1H), 7.30 (td, *J* = 7.5, 1.1 Hz, 1H), 5.44 (s, 2H), 5.29 (d, *J* = 9.7 Hz, 1H), 4.78 (d, *J* = 3.6 Hz, 1H), 4.13–4.09 (m, 1H), 3.69–3.65 (m, 1H), 3.07–2.97 (m, 2H), 2.61–2.55 (m, 1H), 2.33 (td, *J* = 14.0, 4.0 Hz, 1H), 2.01–1.97 (m, 1H), 1.85–1.81 (m, 1H), 1.71–1.67 (m, 2H), 1.59–1.56 (m, 1H), 1.41 (s, 3H), 1.40–1.38 (m, 1H), 1.28–1.16 (m, 3H), 0.91 (d, *J* = 6.3 Hz, 3H), 0.89–0.83 (m, 1H), 0.80 (d, *J* = 7.3 Hz, 3H); ^13^C NMR (150 MHz, CDCl_3_) *δ* (ppm): 159.65, 152.64, 144.77, 141.15, 131.43, 127.30, 123.90, 121.98, 121.78, 117.52, 115.70, 103.03, 100.92, 86.84, 80.02, 76.21, 76.00, 75.78, 66.18, 51.47, 47.86, 43.28, 36.34, 35.36, 33.55, 29.79, 25.57, 25.14, 23.62, 23.34, 19.33, 11.93; ESI-HRMS [M + Na]^+^: (*m*/*z*) Calcd. for C_29_H_35_N_3_O_7_Na: 560.2373. Found: 560.2417.

*6-bromo-3-((4-(2-((10S)-dihydroartemisininoxy)ethyl)-1H-1,2,3-triazol-1-yl)-methyl)-2H-1-chromen-2-one* (**2b**) White solid; m.p. 138–140 °C; yield 52%; ^1^H NMR (600 MHz, CDCl_3_) δ (ppm): 7.64 (dd, *J* = 8.8, 2.3 Hz, 1H), 7.61 (d, *J* = 2.3 Hz, 1H), 7.60 (s, 1H), 7.52 (s, 1H), 7.23 (d, *J* = 8.8 Hz, 1H), 5.43 (s, 2H), 5.28 (s, 1H), 4.79 (d, *J* = 3.5 Hz, 1H), 4.13–4.09 (m, 1H), 3.70–3.66 (m, 1H), 3.07–2.98 (m, 2H), 2.62–2.57 (m, 1H), 2.34 (td, *J* = 14.0, 4.0 Hz, 1H), 2.01–1.98 (m, 1H), 1.86–1.81 (m, 1H), 1.71–1.67 (m, 2H), 1.60–1.56 (m, 1H), 1.42 (s, 3H), 1.40–1.37 (m, 1H), 1.27–1.17 (m, 3H), 0.92 (d, *J* = 6.2 Hz, 3H), 0.89–0.85 (m, 1H), 0.82 (d, *J* = 7.3 Hz, 3H); ^13^C NMR (150 MHz, CDCl_3_) *δ* (ppm): 158.90, 151.40, 144.86, 139.50, 134.15, 129.51, 123.31, 121.76, 118.99, 117.41, 116.51, 103.06, 100.92, 86.85, 80.00, 76.20, 75.99, 75.78, 66.18, 51.45, 47.72, 43.26, 36.37, 35.35, 33.55, 29.78, 25.56, 25.14, 23.63, 23.35, 19.32, 11.95; ESI-HRMS [M + Na]^+^: (*m*/*z*) Calcd. for C_29_H_34_BrN_3_O_7_Na: 638.1478. Found: 638.1508.

*6-chloro-3-((4-(2-((10S)-dihydroartemisininoxy)ethyl)-1H-1,2,3-triazol-1-yl)-methyl)-2H-1-chromen-2-one* (**2c**) White solid; m.p. 138–140 °C; yield 52%; ^1^H NMR (600 MHz, CDCl_3_) δ (ppm): 7.60 (s, 1H), 7.53 (s, 1H), 7.50 (dd, *J* = 8.8, 2.5 Hz, 1H), 7.45 (d, *J* = 2.4 Hz, 1H), 7.29 (d, *J* = 8.8 Hz, 1H), 5.43 (d, *J* = 1.1 Hz, 2H), 5.28 (s, 1H), 4.79 (d, *J* = 3.6 Hz, 1H), 4.13–4.09 (m, 1H), 3.70–3.66 (m, 1H), 3.05–2.99 (m, 2H), 2.61–2.58 (m, 1H), 2.37–2.31 (m, 1H), 2.01–1.98 (m, 1H), 1.86–1.81 (m, 1H), 1.70–1.68 (m, 2H), 1.60–1.56 (m, 1H), 1.42 (s, 3H), 1.41–1.36 (m, 1H), 1.26–1.17 (m, 3H), 0.92 (d, *J* = 6.2 Hz, 3H), 0.89–0.85 (m, 1H), 0.82 (d, *J* = 7.3 Hz, 3H); ^13^C NMR (150 MHz, CDCl_3_) *δ* (ppm): 158.97, 150.93, 144.87, 139.62, 131.34, 129.25, 126.47, 123.35, 121.76, 118.51, 117.14, 103.06, 100.93, 86.85, 80.00, 76.20, 75.99, 75.78, 66.19, 51.45, 47.72, 43.27, 36.37, 35.35, 33.56, 29.79, 25.57, 25.14, 23.63, 23.35, 19.32, 11.95; ESI-HRMS [M + Na]^+^: (*m*/*z*) Calcd. for C_29_H_34_ClN_3_O_7_Na: 594.1983. Found: 594.2002.

*6,8-dichloro-3-((4-(2-((10S)-dihydroartemisininoxy)ethyl)-1H-1,2,3-triazol-1-yl)-methyl)-2H-1-chromen-2-one* (**2d**) White solid; m.p. 101–103 °C; yield 50%; ^1^H NMR (600 MHz, CDCl_3_) δ (ppm): 7.60 (d, *J* = 2.2 Hz, 2H), 7.52 (s, 1H), 7.37 (d, *J* = 2.3 Hz, 1H), 5.45 (s, 2H), 5.27 (s, 1H), 4.79 (d, *J* = 3.5 Hz, 1H), 4.13–4.08 (m, 1H), 3.71–3.67 (m, 1H), 3.04–3.01 (m, 2H), 2.61–2.59 (m, 1H), 2.34 (td, *J* = 14.0, 4.0 Hz, 1H), 2.02–1.98 (m, 1H), 1.85–1.82 (m, 1H), 1.71–1.68 (m, 2H), 1.60–1.58 (m, 1H), 1.42 (s, 3H), 1.41–1.36 (m, 1H), 1.26–1.17 (m, 3H), 0.92 (d, *J* = 6.1 Hz, 3H), 0.90–0.85 (m, 1H), 0.82 (d, *J* = 7.3 Hz, 3H); ^13^C NMR (150 MHz, CDCl_3_) *δ* (ppm): 157.93, 146.90, 144.92, 139.29, 131.34, 129.12, 125.04, 124.16, 121.82, 121.70, 119.30, 103.07, 100.94, 86.86, 79.99, 76.20, 75.99, 75.78, 66.17, 51.44, 47.57, 43.26, 36.39, 35.35, 33.55, 29.78, 25.53, 25.13, 23.63, 23.35, 19.32, 11.95; ESI-HRMS [M + Na]^+^: (*m*/*z*) Calcd. for C_29_H_33_Cl_2_N_3_O_7_Na: 628.1593. Found: 628.1360.

*6,8-dibromo-3-((4-(2-((10S)-dihydroartemisininoxy)ethyl)-1H-1,2,3-triazol-1-yl)-methyl)-2H-1-chromen-2-one (***2e**) White solid; m.p. 93–95 °C; yield 44%; ^1^H NMR (600 MHz, CDCl_3_) δ (ppm): 7.83 (d, *J* = 2.1 Hz, 1H), 7.53 (s, 1H), 7.49 (d, *J* = 2.2 Hz, 1H), 7.42 (s, 1H), 5.38 (s, 2H), 5.21 (s, 1H), 4.73 (d, *J* = 3.4 Hz, 1H), 4.06–4.02 (m, 1H), 3.64–3.60 (m, 1H), 2.97–2.94 (m, 2H), 2.56–2.51 (m, 1H), 2.30–2.25 (m, 1H), 1.95–1.91 (m, 1H), 1.79–1.75 (m, 1H), 1.64–1.60 (m, 2H), 1.52 (dd, *J* = 13.2, 3.3 Hz, 1H), 1.35 (s, 3H), 1.34–1.29 (m, 1H), 1.19–1.11 (m, 3H), 0.85 (d, *J* = 6.1 Hz, 3H), 0.83–0.78 (m, 1H), 0.75 (d, *J* = 7.4 Hz, 3H); ^13^C NMR (150 MHz, CDCl_3_) *δ* (ppm): 158.01, 148.41, 144.96, 139.16, 136.85, 128.75, 124.12, 121.77, 119.74, 116.47, 110.26, 103.07, 100.93, 86.86, 80.00, 76.21, 76.00, 75.79, 66.19, 51.44, 47.48, 43.26, 36.39, 35.35, 33.55, 29.78, 25.56, 25.13, 23.63, 23.35, 19.33, 11.96; ESI-HRMS [M + Na]^+^: (*m*/*z*) Calcd. for C_29_H_33_Br_2_N_3_O_7_Na: 716.0583. Found: 716.0381.

#### 4.1.2. General Procedure for the Synthesis of Compounds **3f**–**3i** and **4f**–**4i**

Substituted coumarin (**f**–**i**) (10 mmol), substituted DHA (**III** or **IV**) (10 mmol) and triethylamine (10 mmol) were dissolved in 20 mL CH_2_Cl_2_. In addition, CuI (100 mg) was added and the reaction mixture was stirred at room temperature for 8 h under the protection of nitrogen. The mixture was filtered, washed with water, dried over Na_2_SO_4_ and evaporated to dryness. The crude product was purified through column chromatography.

*7-hydroxy-4-((4-((10S)-dihydroartemisininoxy)methyl-1H-1,2,3-triazol-1-yl)-methyl)-2H-1-chromen-2-one* (**3f**) White solid; m.p. 165–167 °C; yield 54%; ^1^H NMR (600 MHz, CDCl_3_) δ (ppm): 9.61 (s, 1H), 7.68 (d, *J* = 8.8 Hz, 1H), 7.67 (s, 1H), 6.99 (dd, *J* = 8.8, 2.4 Hz, 1H), 6.85 (d, *J* = 2.4 Hz, 1H), 6.15 (s, 1H), 5.76 (d, *J* = 15.4 Hz, 1H), 5.57 (d, *J* = 15.3 Hz, 1H), 5.20 (s, 1H), 4.87 (d, *J* = 1.9 Hz, 1H), 4.86 (d, *J* = 7.4 Hz, 1H), 4.68 (d, *J* = 12.9 Hz, 1H), 2.62–2.60 (m, 1H), 2.34–2.29 (m, 1H), 2.02−1.98 (m, 1H), 1.82–1.78 (m, 1H), 1.66−1.61 (m, 3H), 1.61−1.57 (m, 1H), 1.45–1.42 (m, 1H), 1.40 (s, 3H), 1.20−1.08 (m, 3H), 0.84 (d, *J* = 7.4 Hz, 3H), 0.82 (d, *J* = 6.1 Hz, 3H); ^13^C NMR (150 MHz, CDCl_3_) *δ* (ppm): 160.88, 159.75, 154.96, 146.45, 145.19, 124.19, 122.29, 112.78, 111.57, 108.80, 103.21, 103.04, 101.40, 86.91, 79.92, 76.20, 75.99, 75.78, 60.44, 51.32, 50.16, 43.18, 36.28, 35.26, 33.42, 29.74, 25.00, 23.53, 23.32, 19.26, 11.89; ESI-HRMS [M + Na]^+^: (*m*/*z*) Calcd. for C_28_H_33_N_3_O_8_Na: 562.2165. Found: 562.2172.

*5,7-dimethyl-4-((4-((10S)-dihydroartemisininoxy)methyl-1H-1,2,3-triazol-1-yl)-methyl)-2H-1-chromen-2-one* (**3g**) White solid; m.p. 133~135 °C; yield 57%; ^1^H NMR (600 MHz, CDCl_3_) δ (ppm): 7.56 (s, 1H), 7.07 (s, 1H), 6.95 (s, 1H), 5.90 (d, *J* = 17.0 Hz, 1H), 5.86 (d, *J* = 17.0 Hz, 1H), 5.51 (s, 1H), 5.41 (s, 1H), 4.98 (d, *J* = 12.8 Hz, 1H), 4.93 (d, *J* = 3.5 Hz, 1H), 4.73 (d, *J* = 12.8 Hz, 1H), 2.68 (s, 3H), 2.40 (s, 3H), 2.35 (dd, *J* = 14.0, 4.0 Hz, 1H), 2.05−2.01 (m, 1H), 1.88−1.85 (m, 1H), 1.75–1.70 (m, 2H), 1.60 (dd, *J* = 13.2, 3.4 Hz, 1H), 1.48–1.46 (m, 1H), 1.43 (s, 3H), 1.32−1.20 (m, 5H), 0.93 (d, *J* = 6.3 Hz, 3H), 0.89 (d, *J* = 7.4 Hz, 3H); ^13^C NMR (150 MHz, CDCl_3_) *δ* (ppm): 158.67, 154.26, 149.33, 145.35, 142.10, 133.93, 129.26, 122.10, 115.63, 113.36, 112.80, 103.16, 100.93, 86.98, 80.05, 76.21, 76.00, 75.79, 60.80, 52.05, 51.48, 43.31, 36.34, 35.36, 33.50, 29.78, 25.11, 23.61, 23.44, 23.28, 20.24, 19.31, 12.01; ESI-HRMS [M + Na]^+^: (*m*/*z*) Calcd. for C_30_H_37_N_3_O_7_Na: 574.2539. Found: 574.2553.

*7-methyl-4-((4-((10S)-dihydroartemisininoxy)methyl-1H-1,2,3-triazol-1-yl)-methyl)-2H-1-chromen-2-one* (**3h**) White solid; m.p. 107–109 °C; yield 50%; ^1^H NMR (600 MHz, CDCl_3_) δ (ppm): 7.58 (s, 1H), 7.50 (d, *J* = 8.1 Hz, 1H), 7.19 (s, 1H), 7.11 (d, *J* = 8.1 Hz, 1H), 6.01 (s, 1H), 5.74 (d, *J* = 16.3 Hz, 1H), 5.64 (d, *J* = 16.0 Hz, 1H), 5.30 (s, 1H), 4.92–4.88 (m, 2H), 4.72 (d, *J* = 12.9 Hz, 1H), 2.65–2.61 (m, 1H), 2.45 (s, 3H), 2.34 (td, *J* = 14.0, 4.0 Hz, 1H), 2.04–2.00 (m, 1H), 1.86–1.81 (m, 1H), 1.71–1.66 (m, 2H), 1.58–1.55 (m, 1H), 1.42 (s, 3H), 1.26–1.18 (m, 5H), 0.91 (d, *J* = 5.7 Hz, 3H), 0.86 (d, *J* = 7.3 Hz, 3H); ^13^C NMR (150 MHz, CDCl_3_) *δ* (ppm): 159.01, 152.86, 146.75, 145.42, 143.16, 125.03, 122.23, 121.95, 116.71, 113.56, 113.41, 103.15, 101.10, 86.93, 80.00, 76.21, 75.99, 75.78, 60.90, 51.41, 49.29, 43.27, 36.34, 35.31, 33.47, 29.77, 25.06, 23.58, 23.38, 20.70, 19.30, 11.96; ESI-HRMS [M + Na]^+^: (*m*/*z*) Calcd. for C_29_H_35_N_3_O_7_Na: 560.2373. Found: 560.2375.

*6-methyl-4-((4-((10S)-dihydroartemisininoxy)methyl-1H-1,2,3-triazol-1-yl)-methyl)-2H-1-chromen-2-one* (**3i**) White solid; m.p. 88–90°C; yield 60%; ^1^H NMR (600 MHz, CDCl_3_) δ (ppm): 7.58 (s, 1H), 7.39 (d, *J* = 8.8 Hz, 2H), 7.28 (d, *J* = 8.3 Hz, 1H), 6.01 (s, 1H), 5.75 (d, *J* = 16.3 Hz, 1H), 5.66 (d, *J* = 16.3 Hz, 1H), 5.32 (s, 1H), 4.92 (d, *J* = 12.8 Hz, 1H), 4.90 (d, *J* = 3.5 Hz, 1H), 4.73 (d, *J* = 12.8 Hz, 1H), 2.67–2.60 (m, 1H), 2.41 (s, 3H), 2.34 (td, *J* = 14.0, 4.0 Hz, 1H), 2.04–1.99 (m, 1H), 1.86–1.81 (m, 1H), 1.72–1.68 (m, 2H), 1.57 (dd, *J* = 13.2, 3.1 Hz, 1H), 1.42 (s, 3H), 1.26–1.19 (m, 5H), 0.91 (d, *J* = 5.7 Hz, 3H), 0.86 (d, *J* = 7.3 Hz, 3H); ^13^C NMR (150 MHz, CDCl_3_) *δ* (ppm): 158.91, 150.87, 146.72, 145.41, 133.69, 132.71, 122.26, 121.95, 116.32, 115.68, 114.29, 103.15, 101.05, 86.94, 80.00, 76.21, 76.00, 75.78, 60.86, 51.42, 49.17, 43.27, 36.34, 35.31, 33.47, 29.76, 25.07, 23.59, 23.39, 20.01, 19.30, 11.96; ESI-HRMS [M + Na]^+^: (*m*/*z*) Calcd. for C_29_H_35_N_3_O_7_Na: 560.2373. Found: 560.2403.

*7-hydroxy-4-((4-(2-((10S)-dihydroartemisininoxy)ethyl)-1H-1,2,3-triazol-1-yl)-methyl)-2H-1-chromen-2-one* (**4f**) White solid; m.p. 121–123 °C; yield 54%; ^1^H NMR (600 MHz, CDCl_3_) δ (ppm): 9.98 (s, 1H), 7.68 (d, *J* = 8.8 Hz, 1H), 7.49 (s, 1H), 7.01 (dd, *J* = 8.8, 2.4 Hz, 1H), 6.85 (d, *J* = 2.4 Hz, 1H), 6.16 (s, 1H), 5.68–5.59 (m, 2H), 5.20 (s, 1H), 4.71 (d, *J* = 3.5 Hz, 1H), 4.08–4.04 (m, 1H), 3.67–3.63 (m, 1H), 3.03–2.94 (m, 2H), 2.56–2.53 (m, 1H), 2.33 (td, *J* = 14.1, 4.0 Hz, 1H), 2.03–1.99 (m, 1H), 1.86–1.82 (m, 1H), 1.58–1.54 (m, 2H), 1.51–1.48 (m, 1H), 1.40 (s, 3H), 1.40–1.35 (m, 2H), 1.27–1.25 (m, 1H), 1.20–1.16 (m, 2H), 0.88 (d, *J* = 5.6 Hz, 3H), 0.68 (d, *J* = 7.3 Hz, 3H); ^13^C NMR (150 MHz, CDCl_3_) *δ* (ppm): 161.02, 159.72, 155.05, 146.56, 145.44, 124.28, 121.23, 112.79, 111.51, 108.74, 103.16, 103.05, 100.88, 86.87, 79.91, 76.20, 75.99, 75.78, 65.75, 51.37, 50.15, 43.11, 36.37, 35.30, 33.45, 29.66, 25.24, 25.09, 23.62, 23.29, 19.29, 11.73; ESI-HRMS [M + Na]^+^: (*m*/*z*) Calcd. for C_29_H_35_N_3_O_8_Na: 576.2322. Found: 576.2352.

*5,7-dimethyl-4-((4-(2-((10S)-dihydroartemisininoxy)ethyl)-1H-1,2,3-triazol-1-yl)-methyl)-2H-1-chromen-2-one* (**4g**) White solid; m.p. 135–137 °C; yield 52%; ^1^H NMR (600 MHz, CDCl_3_) δ (ppm): 7.41 (s, 1H), 7.06 (s, 1H), 6.94 (s, 1H), 5.84 (s, 2H), 5.54 (s, 1H), 5.28 (s, 1H), 4.80 (d, *J* = 3.4 Hz, 1H), 4.15–4.10 (m, 1H), 3.73–3.69 (m, 1H), 3.11–3.00 (m, 2H), 2.66 (s, 3H), 2.61–2.58 (m, 1H), 2.40 (s, 3H), 2.35 (td, *J* = 14.1, 4.0 Hz, 1H), 2.03–1.99 (m, 1H), 1.89–1.84 (m, 1H), 1.70–1.63 (m, 2H), 1.59–1.55 (m, 1H), 1.49–1.43 (m, 1H), 1.42 (s, 3H), 1.29–1.18 (m, 3H), 0.93 (d, *J* = 6.3 Hz, 3H), 0.87 (dd, *J* = 12.4, 4.6 Hz, 1H), 0.80 (d, *J* = 7.3 Hz, 3H); ^13^C NMR (150 MHz, CDCl_3_) *δ* (ppm): 158.63, 154.30, 149.34, 145.42, 142.09, 133.98, 129.26, 121.15, 115.62, 113.33, 112.98, 103.07, 100.86, 86.87, 80.00, 76.21, 76.00, 75.79, 65.93, 52.03, 51.47, 43.25, 36.36, 35.37, 33.55, 29.76, 25.58, 25.15, 23.66, 23.32, 23.21, 20.24, 19.35, 11.98; ESI-HRMS [M + Na]^+^: (*m*/*z*) Calcd. for C_31_H_39_N_3_O_7_Na: 588.2686. Found: 588.2680.

*7-methyl-4-((4-(2-((10S)-dihydroartemisininoxy)ethyl)-1H-1,2,3-triazol-1-yl)-methyl)-2H-1-chromen-2-one* (**4h**) White solid; m.p. 100–102 °C; yield 53%; ^1^H NMR (600 MHz, CDCl_3_) δ (ppm): 7.48 (d, *J* = 8.1 Hz, 1H), 7.40 (s, 1H), 7.18 (s, 1H), 7.11 (d, *J* = 8.2 Hz, 1H), 6.00 (s, 1H), 5.68–5.62 (m, 2H), 5.29 (d, *J* = 12.6 Hz, 1H), 4.76 (d, *J* = 3.5 Hz, 1H), 4.11–4.08 (m, 1H), 3.69–3.65 (m, 1H), 3.06–2.96 (m, 2H), 2.60–2.54 (m, 1H), 2.45 (s, 3H), 2.34 (td, *J* = 14.0, 4.0 Hz, 1H), 2.05–1.99 (m, 1H), 1.88–1.83 (m, 1H), 1.66–1.63 (m, 2H), 1.60–1.58 (m, 1H), 1.56–1.53 (m, 1H), 1.41 (s, 3H), 1.27–1.17 (m, 3H), 0.93 (d, *J* = 6.1 Hz, 3H), 0.89–0.84 (m, 1H), 0.74 (d, *J* = 7.3 Hz, 3H); ^13^C NMR (150 MHz, CDCl_3_) *δ* (ppm): 158.98, 152.88, 146.91, 145.50, 143.18, 125.06, 122.27, 120.82, 116.69, 113.56, 113.35, 103.08, 100.86, 86.86, 79.97, 76.21, 76.00, 75.78, 65.94, 51.44, 49.21, 43.21, 36.36, 35.35, 33.54, 29.72, 25.55, 25.14, 23.65, 23.31, 20.69, 19.34, 11.86; ESI-HRMS [M + Na]^+^: (*m*/*z*) Calcd. for C_30_H_37_N_3_O_7_Na: 574.2529. Found: 574.2567.

*6-methyl-4-((4-(2-((10S)-dihydroartemisininoxy)ethyl)-1H-1,2,3-triazol-1-yl)-methyl)-2H-1-chromen-2-one* (**4i**) White solid; m.p. 100–102 °C; yield 43%; ^1^H NMR (600 MHz, CDCl_3_) δ (ppm): 7.40 (s, 1H), 7.40 (s, 1H), 7.38 (d, *J* = 1.9 Hz, 1H), 7.28 (d, *J* = 8.3 Hz, 1H), 6.00 (d, *J* = 1.2 Hz, 1H), 5.67 (s, 2H), 5.29 (s, 1H), 4.77 (d, *J* = 3.4 Hz, 1H), 4.13–4.09 (m, 1H), 3.71–3.67 (m, 1H), 3.05–2.99 (m, 2H), 2.61–2.56 (m, 1H), 2.41 (s, 3H), 2.36–2.32 (m, 1H), 2.03–2.00 (m, 1H), 1.88–1.84 (m, 1H), 1.66 (dd, *J* = 13.7, 3.5 Hz, 1H), 1.61 (d, *J* = 4.2 Hz, 1H), 1.58–1.53 (m, 2H), 1.42 (s, 3H), 1.24–1.16 (m, 3H), 0.93 (d, *J* = 6.2 Hz, 3H), 0.88–0.85 (m, 1H), 0.75 (d, *J* = 7.3 Hz, 3H); ^13^C NMR (150 MHz, CDCl_3_) *δ* (ppm): 158.88, 150.89, 146.89, 145.51, 133.70, 132.73, 122.29, 120.86, 116.31, 115.69, 114.22, 103.09, 100.87, 86.86, 79.98, 76.20, 75.99, 75.78, 65.94, 51.45, 49.06, 43.22, 36.37, 35.35, 33.54, 29.73, 25.57, 25.14, 23.65, 23.31, 20.03, 19.34, 11.86; ESI-HRMS [M + Na]^+^: (*m*/*z*) Calcd. for C_30_H_37_N_3_O_7_Na: 574.2529. Found: 574.2558.

#### 4.1.3. General Procedure for the Synthesis of Compounds **5j**,**5l**–**5o**, and **6j**–**6p**

Substituted coumarin (**j**–**p**) (10 mmol), substituted DHA (**I** or **II**) (10 mmol) and triethylamine (10 mmol) were dissolved in 20 mL CH_2_Cl_2_. In addition, CuI (100 mg) was added and the reaction mixture was stirred at room temperature for 8h under the protection of nitrogen. The mixture was filtered, washed with water, dried over Na_2_SO_4_ and evaporated to dryness. The crude product was purified through column chromatography.

*4-((4-(2-((10S)-dihydroartemisininoxy)ethyl)-1H-1,2,3-triazol-1-yl)-methoxy)-2H-1-chromen-2-one* (**5j**) White solid; m.p. 91–93 °C; yield 51%; ^1^H NMR (600 MHz, CDCl_3_) δ (ppm): 7.79–7.75 (m, 2H), 7.55 (ddd, *J* = 8.7, 7.3, 1.6 Hz, 1H), 7.33 (d, *J* = 8.3 Hz, 1H), 7.25–7.22 (m, 1H), 5.87 (s, 1H), 5.34 (d, *J* = 2.7 Hz, 2H), 5.16 (s, 1H), 4.77 (d, *J* = 3.6 Hz, 1H), 4.72–4.68 (m, 1H), 4.58–4.54 (m, 1H), 4.32–4.29 (m, 1H), 3.87–3.83 (m, 1H), 2.63–2.60 (m, 1H), 2.33 (td, *J* = 14.0, 4.0 Hz, 1H), 2.01–1.97 (m, 1H), 1.87–1.81 (m, 1H), 1.68–1.64 (m, 1H), 1.58–1.55 (m, 1H), 1.53–1.51 (m, 1H), 1.41 (s, 3H), 1.38 (d, *J* = 11.2 Hz, 1H), 1.26–1.18 (m, 3H), 0.91 (d, *J* = 5.9 Hz, 3H), 0.88–0.83 (m, 1H), 0.78 (d, *J* = 7.4 Hz, 3H); ^13^C NMR (150 MHz, CDCl_3_) *δ* (ppm): 163.90, 161.51, 152.34, 140.45, 131.58, 122.86, 122.85, 122.04, 115.80, 114.40, 103.21, 101.15, 90.15, 86.86, 79.73, 76.20, 75.99, 75.78, 65.36, 61.67, 51.32, 49.57, 42.97, 36.35, 35.25, 33.41, 29.53, 25.06, 23.58, 23.32, 19.29, 11.80; ESI-HRMS [M + Na]^+^: (*m*/*z*) Calcd. for C_29_H_35_N_3_O_8_Na: 576.2322. Found: 576.2336.

*5,7-dimethyl-4-((4-(2-((10S)-dihydroartemisininoxy)ethyl)-1H-1,2,3-triazol-1-yl)-methoxy)-2H-1-chromen-2-one* (**5l**) White solid; m.p. 111–113 °C; yield 49%; ^1^H NMR (600 MHz, CDCl_3_) δ (ppm): 7.74 (s, 1H), 6.98 (s, 1H), 6.83 (s, 1H), 5.75 (s, 1H), 5.28 (s, 2H), 5.14 (s, 1H), 4.76 (d, J = 3.6 Hz, 1H), 4.70–4.66 (m, 1H), 4.58–4.54 (m, 1H), 4.31–4.27 (m, 1H), 3.87–3.84 (m, 1H), 2.84 (s, 1H), 2.61–2.59 (m, 1H), 2.53 (s, 3H), 2.36 (s, 3H), 2.35–2.30 (m, 1H), 2.04–1.97 (m, 2H), 1.87–1.82 (m, 1H), 1.58–1.54 (m, 1H), 1.52–1.50 (m, 1H), 1.41 (s, 3H), 1.25–1.17 (m, 3H), 0.91 (d, *J* = 5.9 Hz, 3H), 0.88–0.85 (m, 1H), 0.76 (d, *J* = 7.4 Hz, 3H); ^13^C NMR (150 MHz, CDCl_3_) *δ* (ppm): 166.97, 161.69, 153.91, 141.76, 140.42, 135.57, 127.92, 122.68, 114.43, 110.62, 103.21, 101.15, 89.15, 86.85, 79.74, 76.21, 76.00, 75.79, 65.38, 61.48, 51.33, 49.59, 42.97, 36.36, 35.25, 33.42, 29.53, 25.05, 23.58, 23.30, 22.49, 20.35, 19.30, 11.80; ESI-HRMS [M + Na]^+^: (*m*/*z*) Calcd. for C_31_H_39_N_3_O_8_Na: 604.2635. Found: 604.2645.

*6,8-dimethyl-4-((4-(2-((10S)-dihydroartemisininoxy)ethyl)-1H-1,2,3-triazol-1-yl)-methoxy)-2H-1-chromen-2-one* (**5m**) White solid; m.p. 111–113 °C; yield 49%; ^1^H NMR (600 MHz, CDCl_3_) δ (ppm): 7.77 (s, 1H), 7.39 (s, 1H), 7.22 (s, 1H), 5.83 (s, 1H), 5.32 (d, *J* = 2.5 Hz, 2H), 5.17 (s, 1H), 4.78 (d, *J* = 3.0 Hz, 1H), 4.72–4.68 (m, 1H), 4.59–4.55 (m, 1H), 4.33–4.29 (m, 1H), 3.87–3.83 (m, 1H), 2.64–2.58 (m, 1H), 2.41 (s, 3H), 2.37–2.33 (m, 1H), 2.33 (s, 3H), 2.02–1.98 (m, 1H), 1.88–1.82 (m, 1H), 1.68–1.63 (m, 1H), 1.58–1.53 (m, 1H), 1.53–1.48 (m, 1H), 1.41 (s, 3H), 1.41–1.38 (m, 1H), 1.28–1.16 (m, 3H), 0.91 (d, *J* = 5.9 Hz, 3H), 0.88–0.84 (m, 1H), 0.78 (d, *J* = 7.4 Hz, 3H); ^13^C NMR (150 MHz, CDCl_3_) *δ* (ppm): 165.31, 162.94, 149.91, 141.55, 134.98, 133.03, 125.90, 123.94, 120.25, 114.82, 104.23, 102.18, 90.77, 87.87, 80.76, 77.24, 77.02, 76.81, 66.39, 62.54, 52.35, 50.58, 44.01, 37.37, 36.28, 34.44, 30.57, 26.07, 24.61, 24.35, 20.82, 20.31, 15.65, 12.84; ESI-HRMS [M + Na]^+^: (*m*/*z*) Calcd. for C_31_H_39_N_3_O_8_Na: 604.2635. Found: 604.2639.

*7,8-dimethyl-4-((4-(2-((10S)-dihydroartemisininoxy)ethyl)-1H-1,2,3-triazol-1-yl)-methoxy)-2H-1-chromen-2-one* (**5n**) White solid; m.p. 91–93 °C; yield 51%; ^1^H NMR (600 MHz, CDCl_3_) δ (ppm): 7.76 (s, 1H), 7.50 (d, *J* = 8.1 Hz, 1H), 7.03 (d, *J* = 8.1 Hz, 1H), 5.79 (s, 1H), 5.32 (d, *J* = 3.0 Hz, 2H), 5.15 (s, 1H), 4.77 (d, *J* = 3.5 Hz, 1H), 4.71–4.67 (m, 1H), 4.58–4.53 (m, 1H), 4.33–4.27 (m, 1H), 3.86–3.82 (m, 1H), 2.63–2.58 (m, 1H), 2.37 (s, 3H), 2.36 (s, 3H), 2.34–2.30 (m, 1H), 2.01–1.97 (m, 1H), 1.86–1.81 (m, 1H), 1.68–1.62 (m, 1H), 1.58–1.53 (m, 1H), 1.51 (dd, *J* = 13.4, 3.5 Hz, 1H), 1.41 (s, 3H), 1.39–1.36 (m, 1H), 1.27–1.15 (m, 3H), 0.91 (d, *J* = 5.8 Hz, 3H), 0.88–0.82 (m, 1H), 0.77 (d, *J* = 7.3 Hz, 3H); ^13^C NMR (150 MHz, CDCl_3_) *δ* (ppm): 165.56, 163.09, 151.58, 142.30, 141.69, 125.34, 124.53, 123.85, 119.71, 113.06, 104.24, 102.18, 89.91, 87.88, 80.78, 77.25, 77.04, 76.82, 66.39, 62.61, 52.37, 50.60, 44.02, 37.37, 36.29, 34.45, 30.58, 26.09, 24.61, 24.35, 20.46, 20.33, 12.85, 11.67; ESI-HRMS [M + Na]^+^: (*m*/*z*) Calcd. for C_31_H_39_N_3_O_8_Na: 604.2635. Found: 604.2646.

*8-methyl-4-((4-(2-((10S)-dihydroartemisininoxy)ethyl)-1H-1,2,3-triazol-1-yl)-methoxy)-2H-1-chromen-2-one* (**5o**) White solid; m.p. 84–86 °C; yield 43%; ^1^H NMR (600 MHz, CDCl_3_) δ (ppm): 7.77 (s, 1H), 7.61 (d, *J* = 7.9 Hz, 1H), 7.39 (d, *J* = 7.4 Hz, 1H), 7.13 (t, *J* = 7.7 Hz, 1H), 5.85 (s, 1H), 5.33 (d, *J* = 2.8 Hz, 2H), 5.15 (s, 1H), 4.77 (d, *J* = 3.5 Hz, 1H), 4.71–4.67 (m, 1H), 4.58–4.54 (m, 1H), 4.33–4.27 (m, 1H), 3.86–3.82 (m, 1H), 2.62–2.59 (m, 1H), 2.45 (s, 3H), 2.33 (td, *J* = 14.1, 4.0 Hz, 1H), 2.01–1.97 (m, 1H), 1.86–1.81 (m, 1H), 1.65 (dd, *J* = 14.0, 3.6 Hz, 1H), 1.58–1.54 (m, 1H), 1.51 (dd, *J* = 13.4, 3.5 Hz, 1H), 1.41 (s, 3H), 1.40–1.35 (m, 1H), 1.27–1.17 (m, 3H), 0.90 (d, *J* = 5.8 Hz, 3H), 0.88–0.84 (m, 1H), 0.77 (d, *J* = 7.3 Hz, 3H); ^13^C NMR (150 MHz, CDCl_3_) *δ* (ppm): 165.30, 162.70, 151.74, 141.60, 133.84, 126.28, 123.84, 123.42, 120.66, 115.15, 104.24, 102.18, 90.88, 87.89, 80.77, 77.25, 77.03, 76.82, 66.39, 62.71, 52.36, 50.60, 44.01, 37.38, 36.28, 34.45, 30.57, 26.09, 24.61, 24.35, 20.33, 15.76, 12.84; ESI-HRMS [M + Na]^+^: (*m*/*z*) Calcd. for C_30_H_37_N_3_O_8_Na: 590.2478. Found: 590.2477.

*4-((4-(3-((10S)-dihydroartemisininoxy)propyl)-1H-1,2,3-triazol-1-yl)-methoxy)-2H-1-chromen-2-one* (**6j**) White solid; m.p. 74–76 °C; yield 47%; ^1^H NMR (600 MHz, CDCl_3_) δ (ppm): 7.79 (dd, *J* = 8.0, 1.6 Hz, 1H), 7.72 (s, 1H), 7.55 (ddd, *J* = 8.7, 7.4, 1.6 Hz, 1H), 7.32 (dd, *J* = 8.4, 1.1 Hz, 1H), 7.26–7.23 (m, 1H), 5.86 (s, 1H), 5.41 (s, 1H), 5.34 (s, 2H), 4.79 (d, *J* = 3.6 Hz, 1H), 4.56–4.46 (m, 2H), 3.93–3.89 (m, 1H), 3.44–3.41 (m, 1H), 2.69–2.63 (m, 1H), 2.39–2.34 (m, 1H), 2.27–2.20 (m, 2H), 2.05–2.01 (m, 1H), 1.91–1.86 (m, 1H), 1.79–1.75 (m, 1H), 1.67–1.64 (m, 1H), 1.51–1.46 (m, 2H), 1.42 (s, 3H), 1.39–1.33 (m, 1H), 1.28–1.22 (m, 2H), 0.96 (d, *J* = 6.4 Hz, 3H), 0.94 (d, *J* = 7.4 Hz, 3H); ^13^C NMR (150 MHz, CDCl_3_) *δ* (ppm): 163.96, 161.59, 152.33, 140.41, 131.55, 122.89, 122.40, 122.13, 115.78, 114.42, 103.18, 101.19, 90.16, 86.93, 79.93, 76.21, 76.00, 75.79, 63.62, 61.66, 51.47, 46.73, 43.23, 36.45, 35.33, 33.53, 29.78, 29.43, 25.12, 23.64, 23.54, 19.33, 12.07; ESI-HRMS [M + Na]^+^: (*m*/*z*) Calcd. for C_30_H_37_N_3_O_8_Na: 590.2478. Found: 590.2463.

*7-methyl-4-((4-(3-((10S)-dihydroartemisininoxy)propyl)-1H-1,2,3-triazol-1-yl)-methoxy)-2H-1-chromen-2-one* (**6k**) White solid; m.p. 80–82 °C; yield 51%; ^1^H NMR (600 MHz, CDCl_3_) δ (ppm): 7.71 (s, 1H), 7.65 (d, *J* = 8.1 Hz, 1H), 7.12 (s, 1H), 7.05 (d, *J* = 8.1 Hz, 1H), 5.80 (s, 1H), 5.41 (s, 1H), 5.32 (s, 2H), 4.79 (d, *J* = 3.6 Hz, 1H), 4.53–4.46 (m, 2H), 3.93–3.89 (m, 1H), 3.44–3.40 (m, 1H), 2.69–2.64 (m, 1H), 2.44 (s, 3H), 2.37 (td, *J* = 14.0, 4.0 Hz, 1H), 2.27–2.21 (m, 2H), 2.05–2.01 (m, 1H), 1.91–1.86 (m, 1H), 1.80–1.75 (m, 2H), 1.68–1.64 (m, 1H), 1.51–1.46 (m, 2H), 1.42 (s, 3H), 1.28–1.24 (m, 3H), 0.96 (d, *J* = 6.4 Hz, 3H), 0.94 (d, *J* = 7.3 Hz, 3H); ^13^C NMR (150 MHz, CDCl_3_) *δ* (ppm): δ 164.21, 161.95, 152.44, 142.81, 140.51, 124.11, 122.39, 121.82, 115.88, 111.91, 103.19, 101.18, 89.25, 86.93, 79.94, 76.20, 75.99, 75.78, 63.62, 61.55, 51.47, 46.73, 43.24, 36.44, 35.33, 33.53, 29.78, 29.42, 25.12, 23.63, 23.53, 20.72, 19.33, 12.07; ESI-HRMS [M + Na]^+^: (*m*/*z*) Calcd. for C_31_H_39_N_3_O_8_Na: 604.2635. Found: 604.2617.

*5,7-dimethyl-4-((4-(3-((10S)-dihydroartemisininoxy)propyl)-1H-1,2,3-triazol-1-yl)-methoxy)-2H-1-chromen-2-one* (**6l**) White solid; m.p. 86–88 °C; yield 42%; ^1^H NMR (600 MHz, CDCl_3_) δ (ppm): 7.68 (s, 1H), 6.98 (s, 1H), 6.83 (s, 1H), 5.74 (s, 1H), 5.40 (s, 1H), 5.28 (s, 2H), 4.78 (d, *J* = 3.7 Hz, 1H), 4.53–4.48 (m, 2H), 3.91–3.87 (m, 1H), 3.42–3.38 (m, 1H), 2.69–2.64 (m, 1H), 2.53 (s, 3H), 2.40–2.33 (m, 4H), 2.25–2.19 (m, 2H), 2.05–2.01 (m, 1H), 1.91–1.86 (m, 1H), 1.80–1.77 (m, 1H), 1.76–1.73 (m, 1H), 1.67–1.64 (m, 1H), 1.51–1.47 (m, 2H), 1.42 (s, 3H), 1.30–1.21 (m, 3H), 0.96 (d, *J* = 6.4 Hz, 3H), 0.94 (d, *J* = 7.4 Hz, 3H); ^13^C NMR (150 MHz, CDCl_3_) *δ* (ppm): 166.99, 161.73, 153.90, 141.72, 140.46, 135.57, 127.92, 122.26, 114.43, 110.63, 103.18, 101.15, 89.14, 86.91, 79.93, 76.21, 76.00, 75.78, 63.49, 61.58, 51.46, 46.66, 43.23, 36.46, 35.33, 33.53, 29.78, 29.42, 25.11, 23.64, 23.54, 22.46, 20.35, 19.33, 12.08; ESI-HRMS [M + Na]^+^: (*m*/*z*) Calcd. for C_32_H_41_N_3_O_8_Na: 618.2791. Found: 618.2827.

*6,8-dimethyl-4-((4-(3-((10S)-dihydroartemisininoxy)propyl)-1H-1,2,3-triazol-1-yl)-methoxy)-2H-1-chromen-2-one* (**6m**) White solid; m.p. 90–92 °C; yield 55%; ^1^H NMR (600 MHz, CDCl_3_) δ (ppm): 7.71 (s, 1H), 7.40 (s, 1H), 7.21 (s, 1H), 5.82 (s, 1H), 5.41 (s, 1H), 5.32 (s, 2H), 4.79 (d, *J* = 3.6 Hz, 1H), 4.55–4.46 (m, 2H), 3.94–3.90 (m, 1H), 3.44–3.40 (m, 1H), 2.69–2.63 (m, 1H), 2.40 (s, 3H), 2.39–2.34 (m, 1H), 2.33 (s, 3H), 2.27–2.21 (m, 2H), 2.06–2.01 (m, 1H), 1.91–1.86 (m, 1H), 1.79–1.77 (m, 1H), 1.76–1.73 (m, 1H), 1.67–1.63 (m, 1H), 1.51–1.45 (m, 2H), 1.42 (s, 3H), 1.37–1.35 (m, 1H), 1.28–1.22 (m, 2H), 0.95 (d, *J* = 6.4 Hz, 3H), 0.94 (d, *J* = 7.4 Hz, 3H); ^13^C NMR (150 MHz, CDCl_3_) *δ* (ppm): 165.37, 163.02, 149.90, 141.55, 134.97, 133.07, 125.88, 123.45, 120.35, 114.83, 104.21, 102.21, 90.79, 87.96, 80.97, 77.25, 77.04, 76.83, 64.68, 62.56, 52.51, 47.77, 44.27, 37.47, 36.37, 34.56, 30.82, 30.47, 26.15, 24.67, 24.57, 20.83, 20.36, 15.66, 13.11; ESI-HRMS [M + Na]^+^: (*m*/*z*) Calcd. for C_32_H_41_N_3_O_8_Na: 618.2791. Found: 618.2808.

*7,8-dimethyl-4-((4-(3-((10S)-dihydroartemisininoxy)propyl)-1H-1,2,3-triazol-1-yl)-methoxy)-2H-1-chromen-2-one* (**6n**) White solid; m.p. 115–117 °C; yield 44%; ^1^H NMR (600 MHz, CDCl_3_) δ (ppm): 7.71 (s, 1H), 7.52 (d, *J* = 8.1 Hz, 1H), 7.04 (d, *J* = 8.1 Hz, 1H), 5.80 (s, 1H), 5.40 (s, 1H), 5.32 (s, 2H), 4.79 (d, *J* = 3.5 Hz, 1H), 4.56–4.45 (m, 2H), 3.93–3.89 (m, 1H), 3.44–3.40 (m, 1H), 2.69–2.64 (m, 1H), 2.39–2.34 (m, 7H), 2.27–2.19 (m, 2H), 2.06–2.01 (m, 1H), 1.92–1.86 (m, 1H), 1.81–1.77 (m, 1H), 1.74 (dd, *J* = 13.4, 3.5 Hz, 1H), 1.67–1.63 (m, 1H), 1.52–1.45 (m, 2H), 1.42 (s, 3H), 1.39–1.34 (m, 1H), 1.27–1.24 (m, 2H), 0.96 (d, *J* = 6.4 Hz, 3H), 0.94 (d, *J* = 7.4 Hz, 3H); ^13^C NMR (150 MHz, CDCl_3_) *δ* (ppm): 165.61, 163.15, 151.58, 142.28, 141.66, 125.37, 124.51, 123.38, 119.78, 113.07, 104.21, 102.21, 89.92, 87.96, 80.97, 77.25, 77.04, 76.82, 64.66, 62.60, 52.51, 47.76, 44.27, 37.47, 36.37, 34.56, 30.82, 30.46, 26.15, 24.67, 24.57, 20.46, 20.37, 13.11, 11.67; ESI-HRMS [M + Na]^+^: (*m*/*z*) Calcd. for C_32_H_41_N_3_O_8_Na: 618.2791. Found: 618.2793.

*8-methyl-4-((4-(3-((10S)-dihydroartemisininoxy)propyl)-1H-1,2,3-triazol-1-yl)-methoxy)-2H-1-chromen-2-one* (**6o**) White solid; m.p. 143–145°C; yield 38%; ^1^H NMR (600 MHz, CDCl_3_) δ (ppm): 7.71 (s, 1H), 7.63 (d, *J* = 8.0 Hz, 1H), 7.39 (d, *J* = 7.3 Hz, 1H), 7.14 (t, *J* = 7.7 Hz, 1H), 5.85 (s, 1H), 5.40 (s, 1H), 5.33 (s, 2H), 4.79 (d, *J* = 3.7 Hz, 1H), 4.55–4.46 (m, 2H), 3.93–3.89 (m, 1H), 3.44–3.40 (m, 1H), 2.70–2.63 (m, 1H), 2.45 (s, 3H), 2.37 (td, *J* = 14.0, 3.9 Hz, 1H), 2.28–2.19 (m, 2H), 2.05–2.01 (m, 1H), 1.92–1.86 (m, 1H), 1.81–1.72 (m, 2H), 1.67–1.64 (m, 1H), 1.53–1.46 (m, 2H), 1.42 (s, 3H), 1.39–1.33 (m, 1H), 1.30–1.22 (m, 2H), 0.96 (d, *J* = 6.4 Hz, 3H), 0.94 (d, *J* = 7.4 Hz, 3H); ^13^C NMR (150 MHz, CDCl_3_) *δ* (ppm): 165.35, 162.77, 151.73, 141.56, 133.82, 126.25, 123.45, 123.40, 120.73, 115.17, 104.22, 102.21, 90.89, 87.96, 80.97, 77.24, 77.03, 76.82, 64.65, 62.69, 52.51, 47.76, 44.27, 37.48, 36.37, 34.56, 30.82, 30.46, 26.15, 24.67, 24.57, 20.37, 15.76, 13.11; ESI-HRMS [M + Na]^+^: (*m*/*z*) Calcd. for C_31_H_39_N_3_O_8_Na: 604.2635. Found: 604.2646.

*6-methyl-4-((4-(3-((10S)-dihydroartemisininoxy)propyl)-1H-1,2,3-triazol-1-yl)-methoxy)-2H-1-chromen-2-one* (**6p**) White solid; m.p. 80–82 °C; yield 45%; ^1^H NMR (600 MHz, CDCl_3_) δ (ppm): 7.72 (s, 1H), 7.56 (s, 1H), 7.35 (dd, *J* = 8.5, 2.0 Hz, 1H), 7.21 (d, *J* = 8.4 Hz, 1H), 5.84 (s, 1H), 5.41 (s, 1H), 5.33 (s, 2H), 4.80 (d, *J* = 3.6 Hz, 1H), 4.55–4.47 (m, 2H), 3.94–3.90 (m, 1H), 3.45–3.41 (m, 1H), 2.70–2.64 (m, 1H), 2.40–2.33 (m, 4H), 2.27–2.21 (m, 2H), 2.06–2.01 (m, 1H), 1.91–1.87 (m, 1H), 1.80–1.73 (m, 2H), 1.65 (dd, *J* = 13.3, 3.4 Hz, 1H), 1.53–1.46 (m, 2H), 1.42 (s, 3H), 1.38–1.34 (m, 1H), 1.28–1.22 (m, 2H), 0.96 (d, *J* = 6.4 Hz, 3H), 0.94 (d, *J* = 7.3 Hz, 3H); ^13^C NMR (150 MHz, CDCl_3_) *δ* (ppm): 165.02, 162.88, 151.52, 141.46, 133.69, 133.59, 123.52, 122.80, 116.56, 115.08, 104.22, 102.22, 91.11, 87.97, 80.97, 77.24, 77.03, 76.82, 64.68, 62.60, 52.51, 47.80, 44.27, 37.48, 36.37, 34.57, 30.82, 30.48, 26.15, 24.67, 24.58, 20.90, 20.37, 13.12; ESI-HRMS [M + Na]^+^: (*m*/*z*) Calcd. for C_31_H_39_N_3_O_8_Na: 604.2635. Found: 604.2621.

### 4.2. Biology

#### 4.2.1. Storage and Preparation of Samples

All the target compounds were dissolved in dimethylsulfoxide and storage under −20 °C. They get to be diluted to different concentration when they will be used.

#### 4.2.2. Cell Culture

After recovery, the cells were cultured in 96-well plates for 24 h at 37 °C in a humidified atmosphere with 95% air and 5% CO_2_. After treated with different tested compounds, the hypoxia groups were placed in a sealed hypoxia incubator chamber (Stemcell Technologies, Inc., Vancouver, BC, Canada) filled with 5% CO_2_ and 95% N_2_ for 24 h, and then transferred to the incubator chamber filled with air for 72 h. On the contrary, the normoxia groups were cultured under air condition for 96h.

Cancer cells HT-29 (Human Colorectal Adenocarcinoma cell line), MDA-MB-231 (Human Breast Cancer cell line), HCT-116 (Human Colorectal Carcinoma cell line), and A549 (Human Lung Adenocarcinomic cell line) were from ATCC.

#### 4.2.3. MTT Assay

When the adherent cells reached 80% confluence, the culture medium was discarded. The cells were digested by trypsin and collected after centrifugation. The fresh culture medium was gently blown into it to form single-cell suspension. Cell suspension will be diluted to 1.5–3 × 10^4^ cells/mL. Each hole of 96-well plates was added 100 μL cell suspension and incubated for 24 h (5% CO_2_; 37 °C). Then the holes were added different concentrations of tested compounds. The early screening concentration is set to 100 μM, 50 μM, 25 μM, 12.5 μM, and 6.25 μM. Three replicates were made for each concentration of the tested compounds. After the cells were grown for 96h, 20 μL MTT (5 mg/mL) was added to each well and incubated for 4 h. The medium was discarded and each well was added 100 μL DMSO to dissolve the formazan blue. Absorbance was measured at 570 nm with a microplate reader (Synergy-HT, BioTek Instruments, Winooski, VT, USA).

Hypoxia condition: The cells were treated with different concentration of tested compounds, placed in hypoxia chamber (Catalog Number 27310, Stemcell Technologies, Inc., Vancouver, BC, Canada), and incubated for 24 h (5% air; 95% N_2_; 37 °C). Then the cells were placed in normoxia condition (5% CO_2_; 37 °C) and incubated for 72 h.

#### 4.2.4. Preparation of N9 Cells

The murine microglial cell line N9 was a kind gift from Dr. P. Ricciardi-Castagnoli (Universita Degli Studi di Milano-Bicocca, Milan, Italy).

N9 cells, which were in the logarithmic phase, were incubated for 24 h by adherent culture. Subsequently, the medium was changed to new one without serum. The cells were treated with different concentration of tested compounds. After specific time in the LPS condition, the supernatants were collected and analyzed.

#### 4.2.5. Nitrite Measurement

The nitrite levels in medium were determined by Griess reaction. The absorbance was measured at 540 nm using a microplate reader.

#### 4.2.6. NO Capture Analysis

SNP was dissolved in PBS to prepare the 100 mM stock solution. In this experiment, SNP solution of 25 μL was added to 975 μL PBS solution which included different concentration of tested compounds. After 60 min under r.t. condition, the concentration of NO_2_^−^ was tested using Griess assay.

#### 4.2.7. Evaluation of Inflammatory Mediator

The secreted level of TNF-α and IL-6 was measured using mouse Th1/Th2/Th17 Cytokine Kit, purchased from BD Pharningen. The data was analyzed by FCAP Array v3.0. IL-2, IL-4, IL-10, IL-17A, and IFN-γ were not detected in the experiment.

#### 4.2.8. Molecular Docking Simulations

The crystallographic structure of TLR4/MD-2 complex was retrieved from the Protein DataBank (PDB ID: 3VQ2), which was optimized by removing water molecules, cofactors and heteroatoms. Receptor grid was generally around the MD-2 active site. Docking calculations were accomplished using AutoDock4. The docking results were further analyzed and visually optimized by Discovery Studio 4.0 and PyMOL.

## Figures and Tables

**Table 1:** IC_50_ (μM) of tested samples in MDA-MB-231, HT-29, HCT-116, and A549 cell lines.

	HT-29		MDA-MB-231		HCT-116		A549	
	Hypoxia	Normoxia	Hypoxia	Normoxia	Hypoxia	Normoxia	Hypoxia	Normoxia
**DOX**	<0.1	0.11	0.69	1.37	>2	1.29	>2	>2
**DHA**	11.74	51.10	45.93	>100	25.89	10.33	>100	>100
**1a**	14.29	18.40	30.47	64.97	49.79	48.90	53.87	83.48
**1b**	13.14	21.35	>100	>100	>100	>100	>100	>100
**1c**	26.37	35.41	32.26	>100	40.38	60.30	48.70	73.49
**1d**	13.34	14.94	33.93	68.85	48.61	60.01	45.42	53.34
**1e**	26.95	15.32	45.46	77.89	>100	54.45	59.75	>100
**2a**	19.42	>100	>100	>100	>100	>100	>100	>100
**2b**	12.09	9.75	36.86	37.74	>100	55.99	60.16	>100
**2c**	19.61	17.46	32.47	43.98	64.37	49.48	66.87	87.15
**2d**	30.81	19.54	47.92	88.46	46.51	53.19	69.67	96.48
**2e**	13.92	38.52	44.14	>100	86.49	94.16	79.37	>100
**3f**	15.46	37.83	63.07	>100	67.74	>100	98.00	>100
**3g**	15.86	23.89	>100	>100	>100	>100	>100	>100
**3h**	32.04	>100	>100	>100	>100	>100	>100	>100
**3i**	8.57	16.36	22.81	>100	>100	64.99	62.27	>100
**4f**	11.30	32.30	78.79	>100	>100	>100	>100	>100
**4g**	9.33	13.85	22.71	>100	25.65	34.50	>100	>100
**4h**	56.35	>100	>100	>100	>100	>100	>100	>100
**4i**	39.02	>100	>100	>100	>100	>100	>100	>100
**5j**	12.25	30.03	69.41	100.00	>100	77.20	>100	>100
**5l**	3.76	2.78	>100	>100	>100	>100	>100	>100
**5m**	5.89	12.88	13.04	14.17	44.81	62.82	1.282	2.670
**5n**	8.90	10.19	11.68	11.04	32.92	25.64	2.443	2.107
**5o**	21.25	16.09	16.54	37.41	38.49	29.69	45.06	50.37
**6j**	7.42	10.49	33.40	67.42	32.84	25.90	49.03	53.37
**6k**	5.32	6.85	14.46	38.56	33.70	28.13	9.49	14.92
**6l**	3.40	8.41	6.85	1.17	27.92	26.90	9.78	6.88
**6m**	5.90	22.43	18.74	24.64	93.53	42.05	20.14	27.87
**6n**	4.19	9.16	10.84	13.35	33.74	22.51	0.039	30.40
**6o**	29.02	81.57	18.57	47.65	42.35	12.09	39.92	78.07
**6p**	12.48	7.49	10.33	22.08	38.05	35.33	18.82	18.85

**Table 2:** IC_50_ of tested samples in LPS-induced N9 cells for the inhibition of NO; IC_20_ of tested samples in N9 cells for cytotoxic activity.

Compound.	NO-IC_50_ (μM)	MTT-IC_20_ (μM)
**1a**	1.46	>10
**1b**	>10	>10
**1c**	5.33	>10
**1d**	1.60	>10
**1e**	0.48	>10
**2a**	0.97	>10
**2b**	0.64	>10
**2c**	2.66	>10
**2d**	1.35	>10
**2e**	0.94	>10
**3f**	0.37	>10
**3g**	>10	>10
**3h**	>10	>10
**3i**	0.72	>10
**4f**	2.03	>10
**4g**	1.36	>10
**4h**	5.69	>10
**4i**	>10	>10
**5j**	1.59	>10
**5l**	3.36	7.13
**5m**	1.08	1.04
**5n**	0.22	2.44
**5o**	0.57	>10
**6j**	0.29	>10
**6k**	1.91	>10
**6l**	0.52	>10
**6m**	5.80	>10
**6n**	1.13	6.53
**6o**	1.43	>10
**6p**	1.42	>10
**DHA**	1.58	>10
**Minocycline**	>10	>10

**Table 3:** The inhibition of compounds (1 μM) on cytokines release (48 h) of the murine microglial cell line N9 induced by lipopolysaccharide (LPS) (1 μg/mL).

Group	TNF-α (pg/mL)	Inhibition (%)	IL-6 (pg/mL)	Inhibition (%)
**Con**	155.54 ± 79.61	--	0	--
**LPS**	4455.87 ± 518.24 ^#^	--	1154.44 ± 148.48 ^#^	--
**2b**	3285.99 ± 1062.40	27.20	797.52 ± 140.74 *	30.92
**2c**	3077.03 ± 375.48	32.06	850.24 ± 112.47	26.35
**5n**	3508.08 ± 368.72	22.04	656.75 ± 79.49 **	43.11
**6p**	3451.29 ± 646.50	23.36	747.80 ± 125.56 *	35.22

^#^*p* < 0.001 compared with control group, * *p* < 0.05 ** *p* < 0.01 compared with LPS group.

## Supplementary Materials

The H and 13 C NMR spectra of synthesized compounds are available online.

## Abbreviations

DHA	Dihydroartemisinin
DCH	Dihydroartemisinin–Coumarin hydrids
CRF	Cancer-related fatigue
DOX	Doxorubicin
CNS	Central nervous system
SNP	Sodium nitroprusside
LPS	Lipopolysaccharide
TLR4	Toll-like receptor 4
