# Supplementary material for: Design, Synthesis, and Mechanism of Dihydroartemisinin–Coumarin Hybrids as Potential Anti-Neuroinflammatory Agents
Source: Molecules. 2019 Apr 28;24(9):1672. doi: 10.3390/molecules24091672 (PMC6539525; doi:10.3390/molecules24091672)

Design, synthesis and mechanism of novel dihydroartemisinin-coumarin hybrids as potential anti-cancer and anti-inflammatory agents

Haonan Yu[1#], Zhuang Hou[1#], Xiaoguang Yang[1], Yanhua Mou[2*] and Chun Guo[1*]

[1]School of Pharmaceutical Engineering, Shenyang Pharmaceutical University, Shenyang 110016, China;

[2] School of life sciences and biological pharmacy, Shenyang Pharmaceutical University, Shenyang 110016, China;

[#]Haonan Yu and Zhuang Hou contributed equally to this work.

***Correspondence to**

Chun Guo, Key Laboratory of Structure-Based Drug Design and Discovery of Ministry of Education, School of Pharmaceutical Engineering, Shenyang Pharmaceutical University, No. 103 Wenhua Road, Shenyang, 110016, Liaoning Province, China. Tel: +86-24-4352-0226, Email: chunguo@syphu.edu.cn; Yanhua Mou, School of life sciences and biological pharmacy, Shenyang Pharmaceutical University, No. 103 Wenhua Road, Shenyang 110016, Liaoning Province, China. Email: mu_hua_jj@sina.com

Authors' emails

yhna380@hotmail.com (HY)

houzhuang8@sina.com (ZH)

1012041601@qq.com (XY);

mu_hua_jj@sina.com (YM)

chunguo@syphu.edu.cn (CG)

^{1}H and ^{13}C NMR Spectrum of intermediates and new compounds

Intermediates

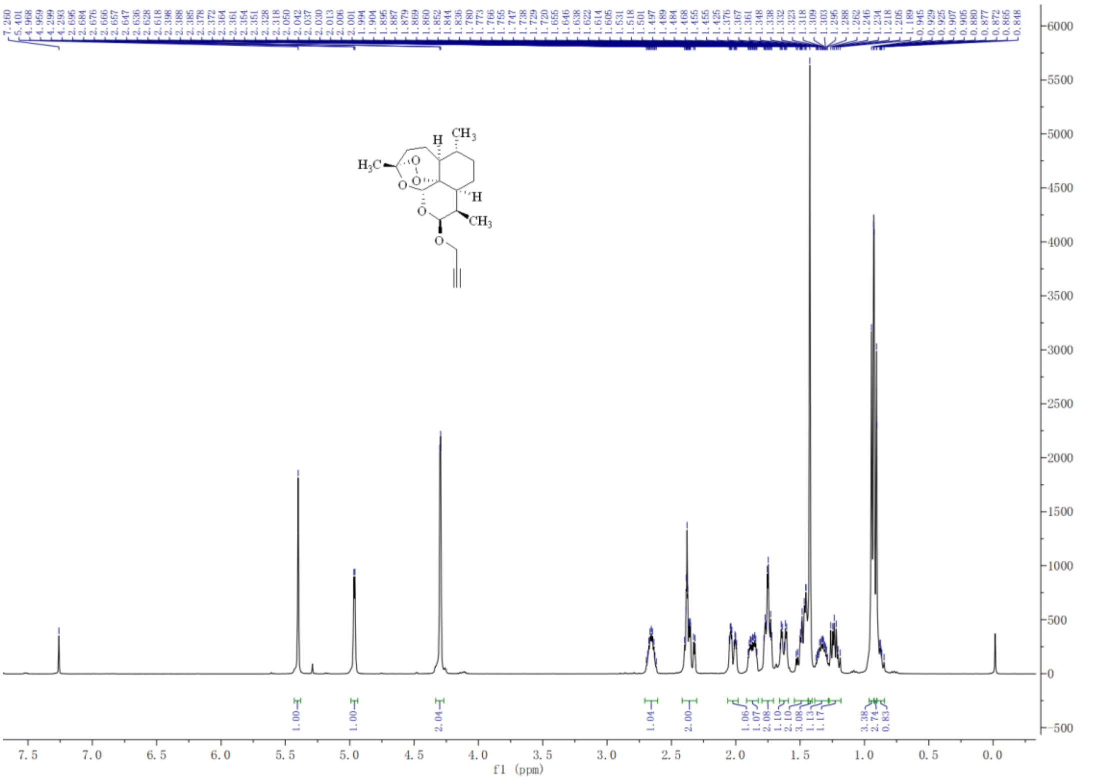

^{1}H NMR (600 MHz, Chloroform-d) δ 5.40 (s, 1H), 4.96 (d, J = 3.5 Hz, 1H), 4.30 (d, J = 2.3 Hz, 2H), 2.70-2.62 (m, 1H), 2.41-2.30 (m, 2H), 2.05-1.99 (m, 1H), 1.90-1.84 (m, 1H), 1.79-1.71 (m, 2H), 1.63 (dd, J = 13.2, 3.4 Hz, 1H), 1.53-1.46 (m, 2H), 1.42 (s, 3H), 1.38-1.29 (m, 1H), 1.26-1.19 (m, 1H), 0.94 (d, J = 6.4 Hz, 3H), 0.92 (d, J = 7.3 Hz, 3H), 0.90-0.83 (m, 1H).

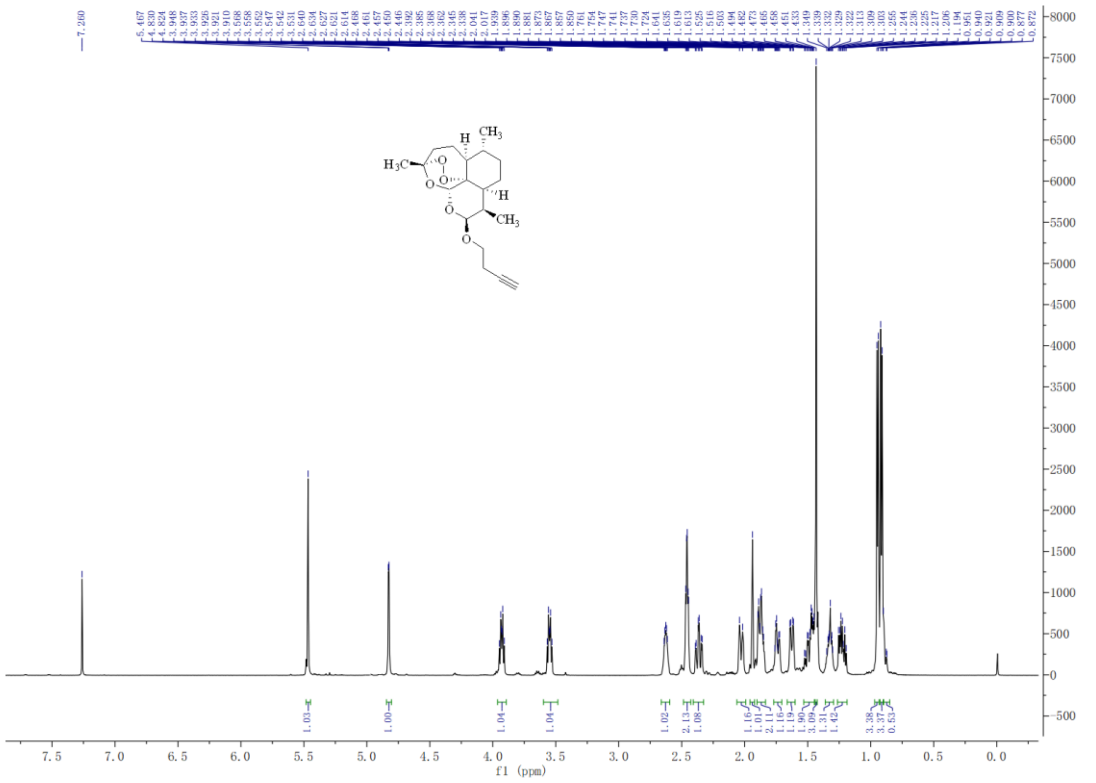

^{1}H NMR (600 MHz, Chloroform-d) δ 5.47 (s, 1H), 4.83 (d, J = 3.5 Hz, 1H), 3.95-3.91 (m, 1H), 3.57-3.53 (m, 1H), 2.64-2.61 (m, 1H), 2.47-2.45 (m, 2H), 2.37 (td, J = 14.1, 4.0 Hz, 1H), 2.03 (d, J = 14.6 Hz, 1H), 1.94 (s, 1H), 1.90-1.85 (m, 2H), 1.77-1.71 (m, 1H), 1.63 (dd, J = 13.3, 3.5 Hz, 1H), 1.53-1.45 (m, 2H), 1.43 (s, 3H), 1.35-1.30 (m, 1H), 1.26-1.19 (m, 1H), 0.95 (d, J = 6.3 Hz, 3H), 0.91 (d, J = 7.3 Hz, 3H), 0.90-0.87 (m, 1H).

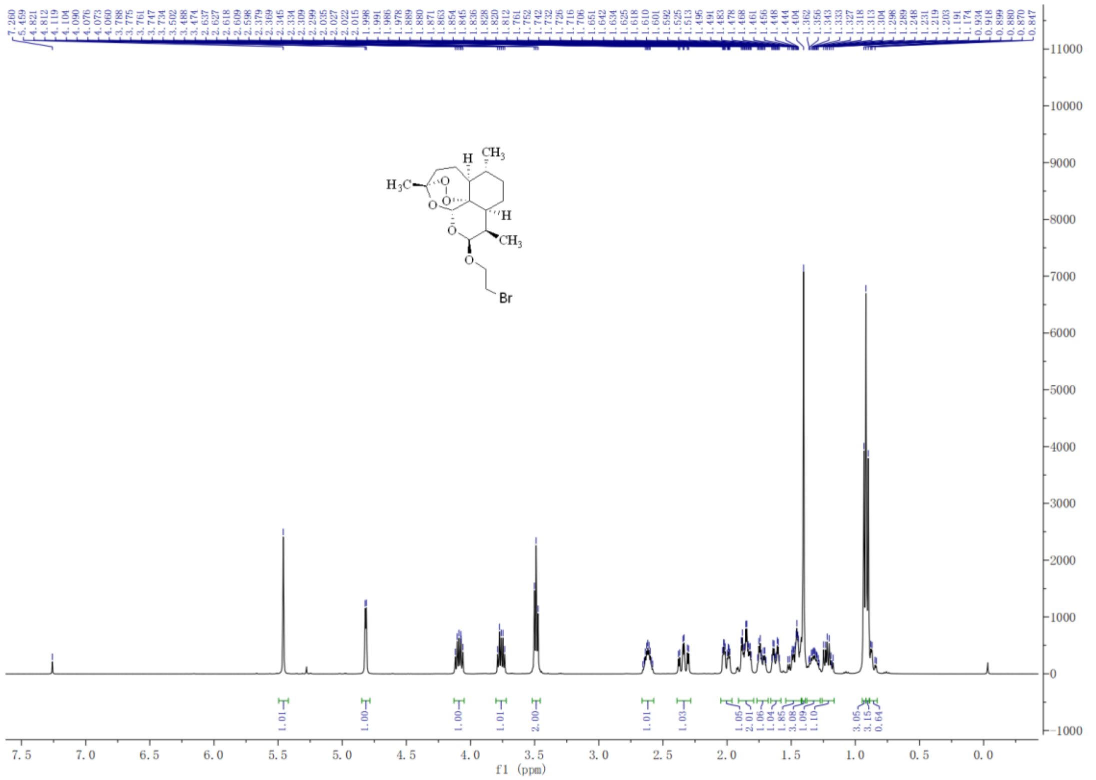

^{1}H NMR (600 MHz, Chloroform-d) δ 5.46 (s, 1H), 4.82 (d, J = 3.5 Hz, 1H), 4.12-4.06 (m, 1H), 3.79-3.73 (m, 1H), 3.49 (t, J = 5.6 Hz, 2H), 2.66-2.58 (m, 1H), 2.34 (td, J = 14.0, 3.9 Hz, 1H), 2.04-1.98 (m, 1H), 1.89-1.81 (m, 2H), 1.76-1.70 (m, 1H), 1.65-1.59 (m, 1H), 1.54-1.42 (m, 2H), 1.40 (s, 3H), 1.36-1.28 (m, 1H), 1.25-1.17 (mi, 1H), 0.92 (t, J = 6.9 Hz, 6H), 0.88-0.84 (m, 1H).

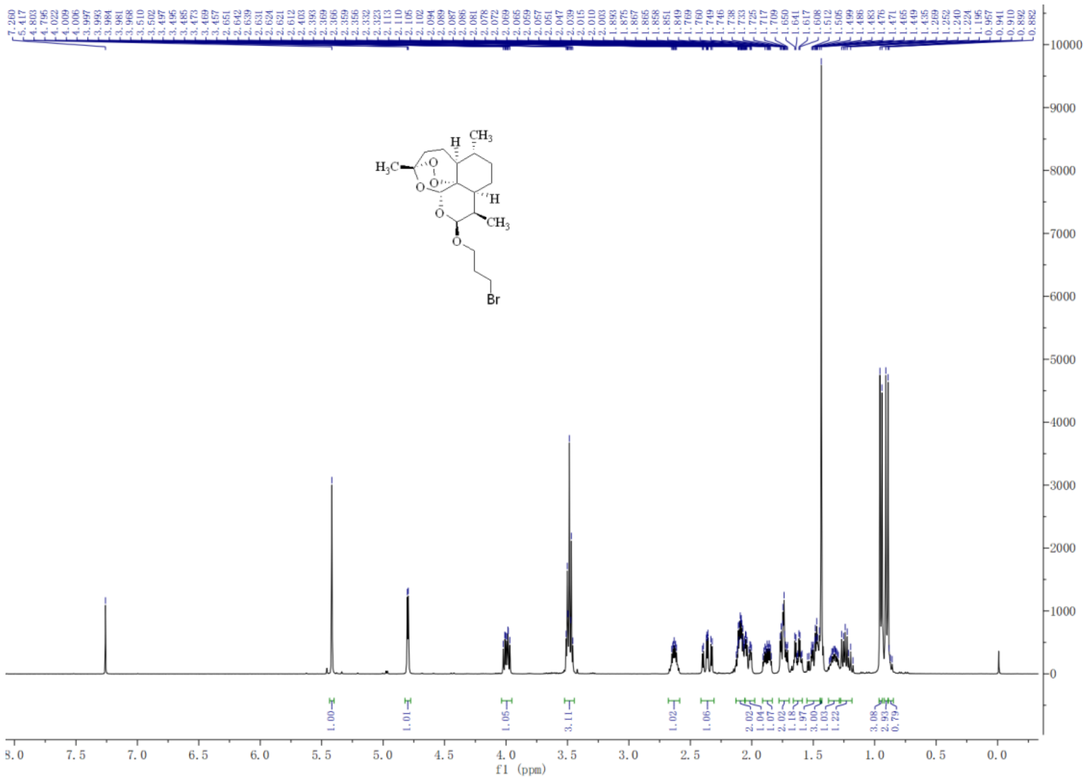

^{1}H NMR (600 MHz, Chloroform-d) δ 5.42 (s, 1H), 4.80 (d, J = 3.4 Hz, 1H), 4.02-3.97 (m, 1H), 3.52-3.45 (m, 3H), 2.65-2.61 (m, 1H), 2.40-2.32 (m, 4.0 Hz, 1H), 2.13-2.00 (m, 2H), 2.05-1.98 (m, 1H), 1.91-1.83 (m, 1H), 1.77-1.71 (m, 2H), 1.65-1.59 (m, 1H), 1.55-1.44 (m, 2H), 1.44 (s, 3H), 1.37-1.30 (m, 1H), 1.27-1.18 (m, 1H), 0.95 (d, J = 6.3 Hz, 3H), 0.90 (d, J = 7.4 Hz, 3H), 0.89-0.86 (m, 1H).

Compound **1a**

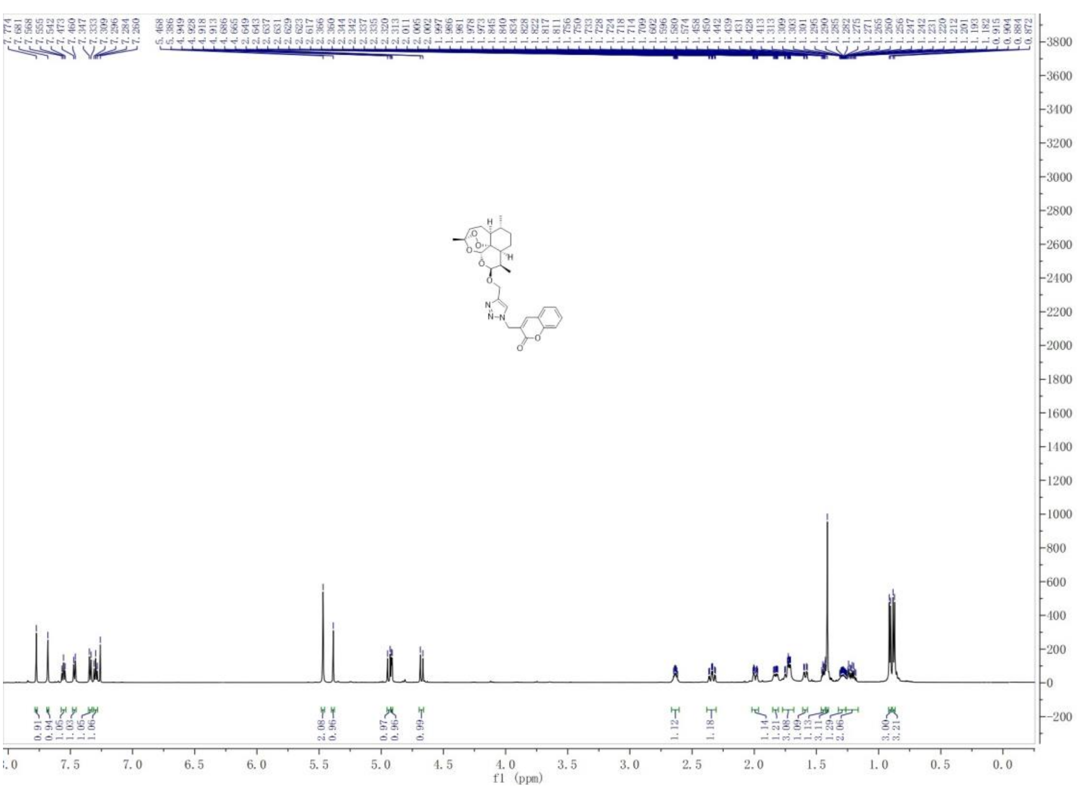
7.774 7.681 7.568 7.555 7.542 7.473 7.460 7.347 7.333 7.309 7.296 7.284 7.260
5.468 5.386 4.949 4.928 4.918 4.913 4.686 4.665 2.649 2.643 2.637 2.631 2.629 2.623 2.617 2.366 2.360 2.344 2.342 2.337 2.335 2.320 2.313 2.011 2.005 2.002 1.997 1.986 1.981 1.978 1.973 1.845 1.840 1.834 1.828 1.822 1.817 1.811 1.756 1.750 1.733 1.728 1.724 1.718 1.714 1.709 1.692 1.596 1.589 1.574 1.458 1.450 1.442 1.439 1.431 1.428 1.413 1.313 1.309 1.303 1.301 1.295 1.290 1.285 1.282 1.275 1.271 1.265 1.260 1.256 1.247 1.242 1.231 1.220 1.212 1.201 1.193 1.182 0.915 0.904 0.884 0.872
0.91 0.94 1.05 1.03 1.05 1.06 2.08 0.96 0.97 0.96 0.99 1.12 1.18 1.14 1.21 3.08 1.09 1.13 3.11 1.29 2.06 3.00 3.21
f1 (ppm)

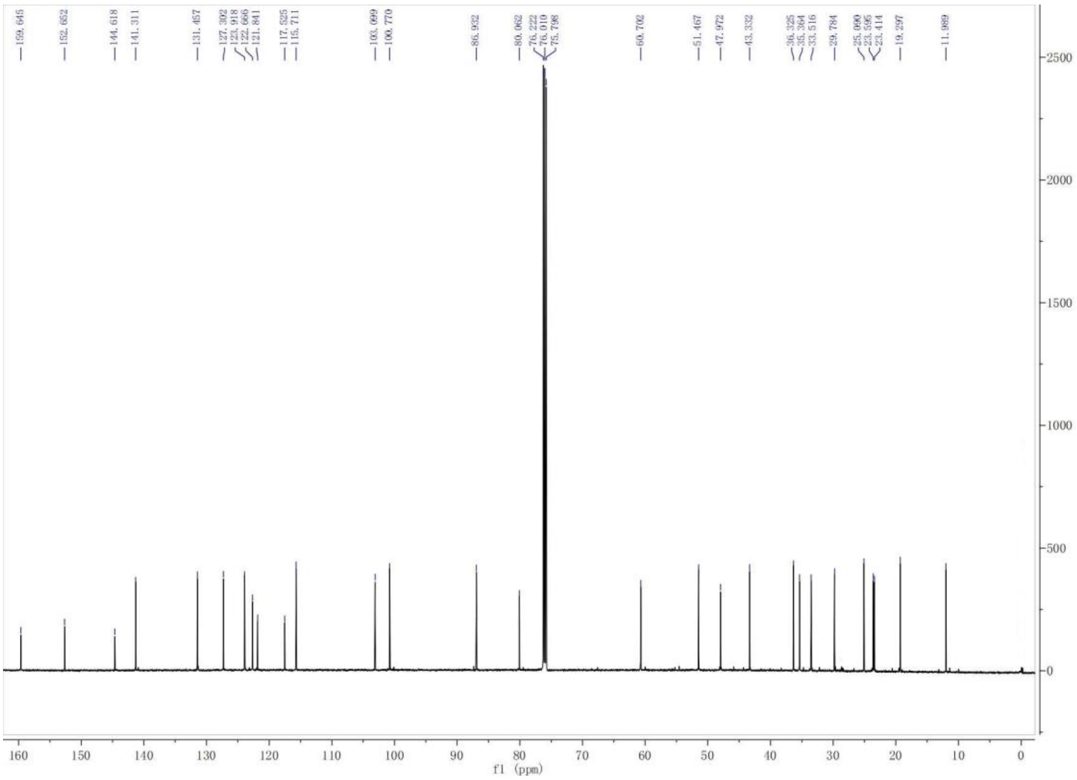
159.645 152.652 144.618 141.311 131.457 127.302 123.918 122.666 121.841 117.525 115.711 103.069 100.770 86.932 80.062 76.222 76.010 75.798 60.702 51.467 47.972 43.332 36.325 35.364 33.516 29.784 25.000 23.595 23.414 19.297 11.989
f1 (ppm)

Compound **1b**

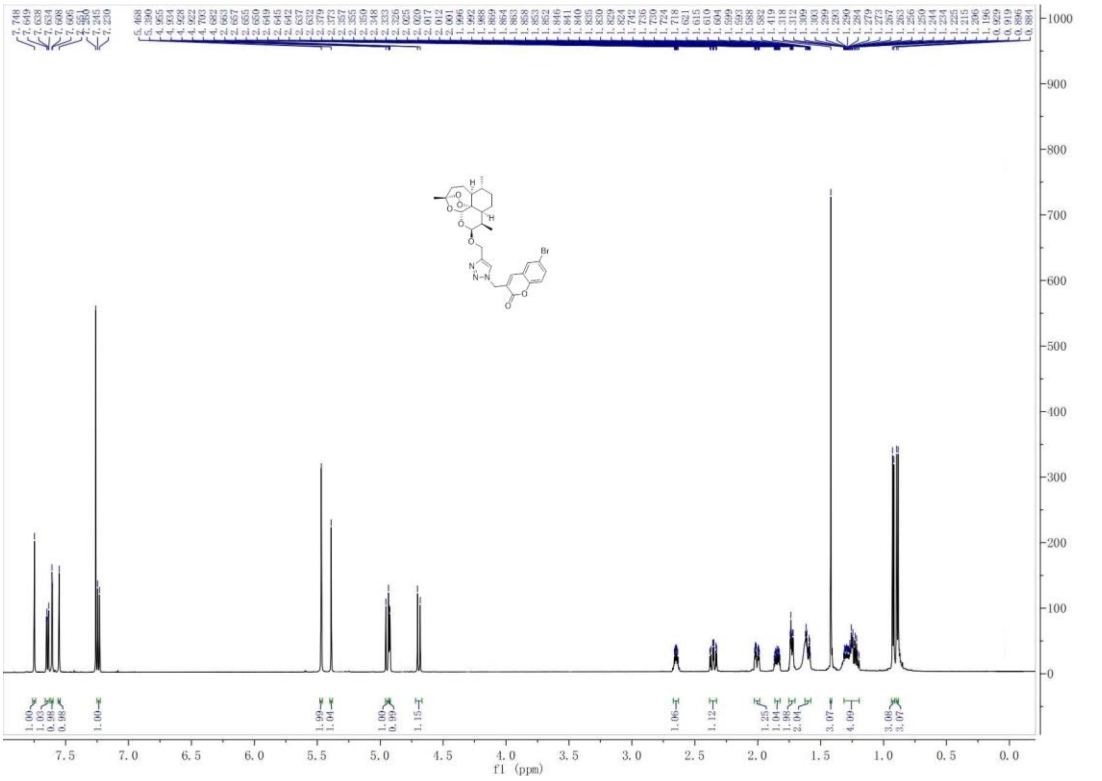

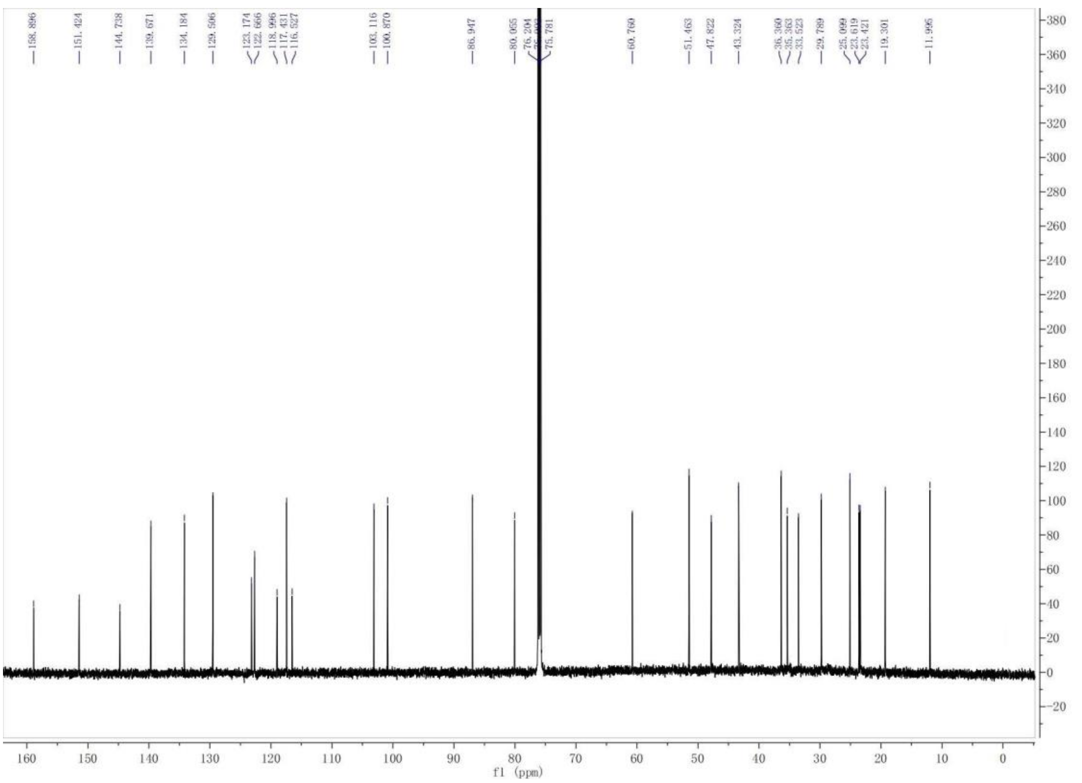

Compound **1c**

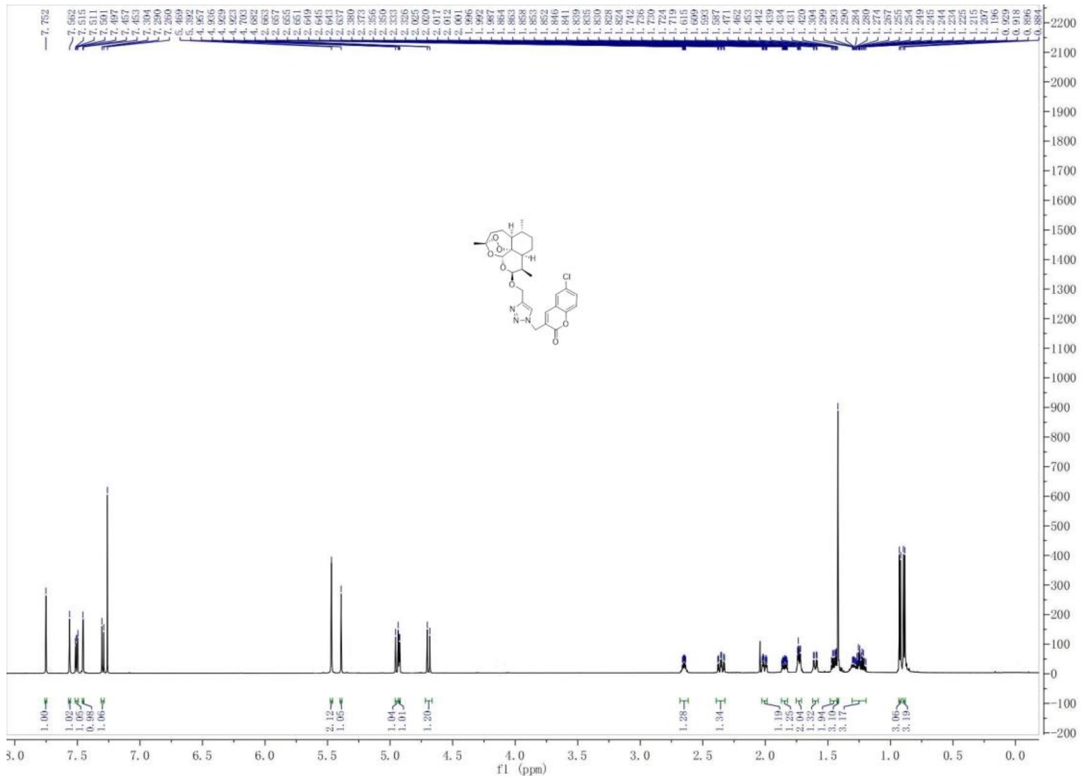

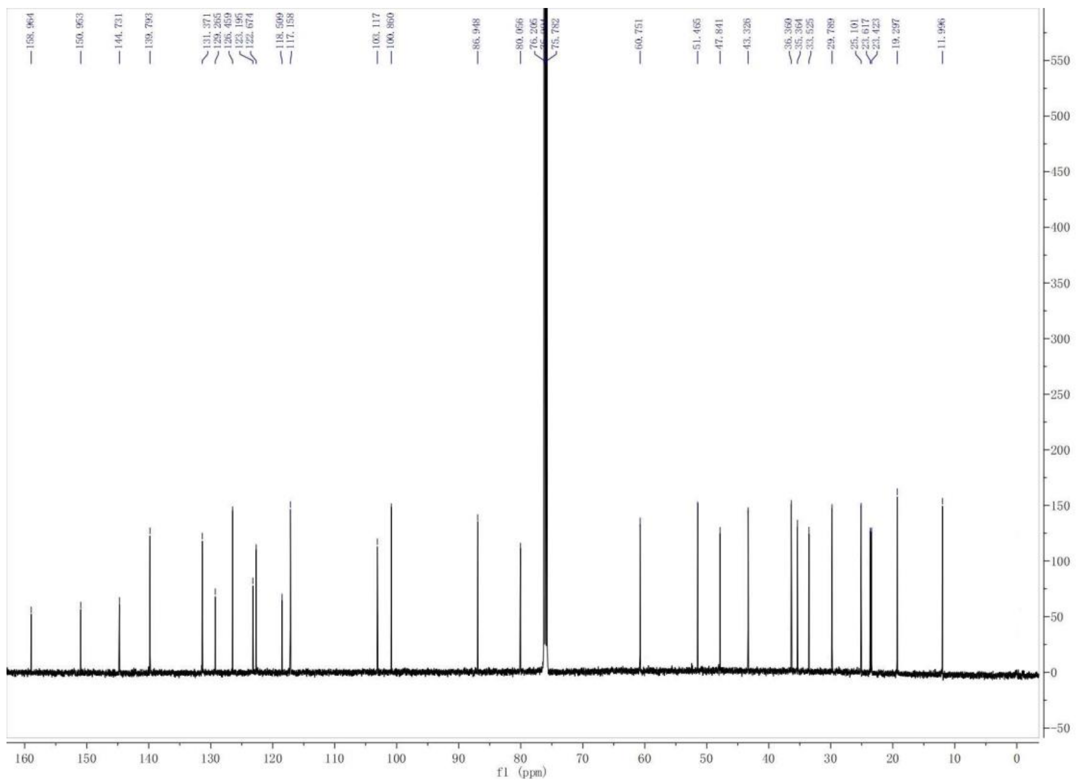

Compound **1d**

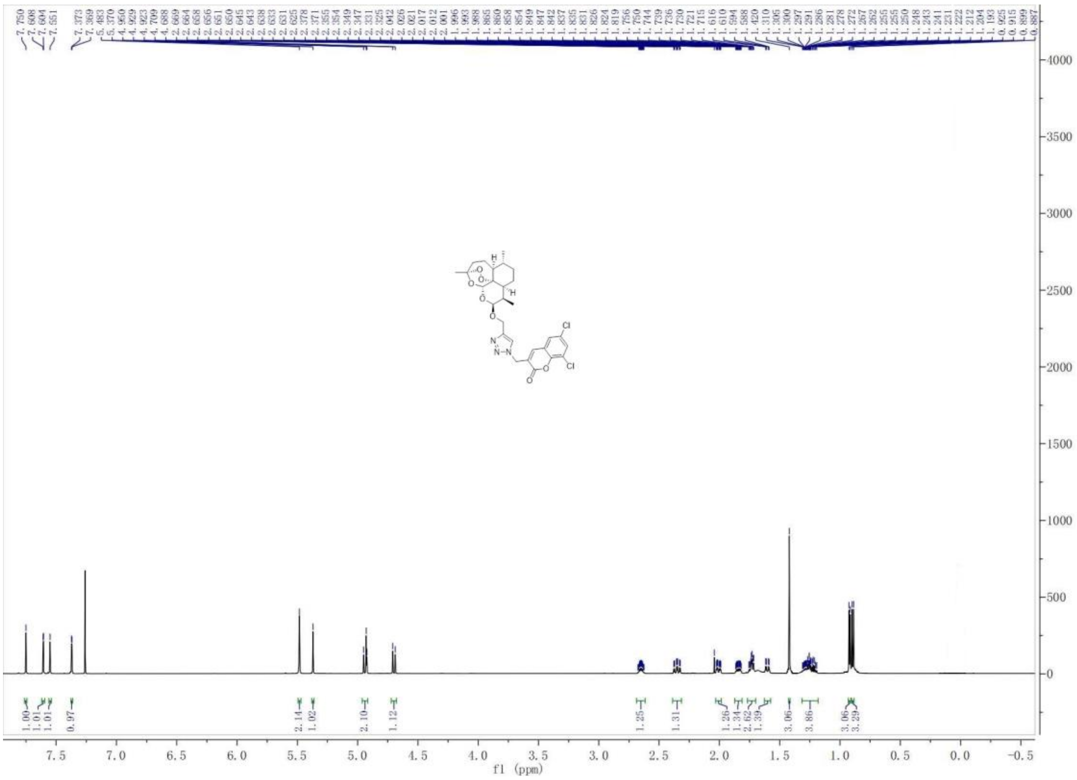

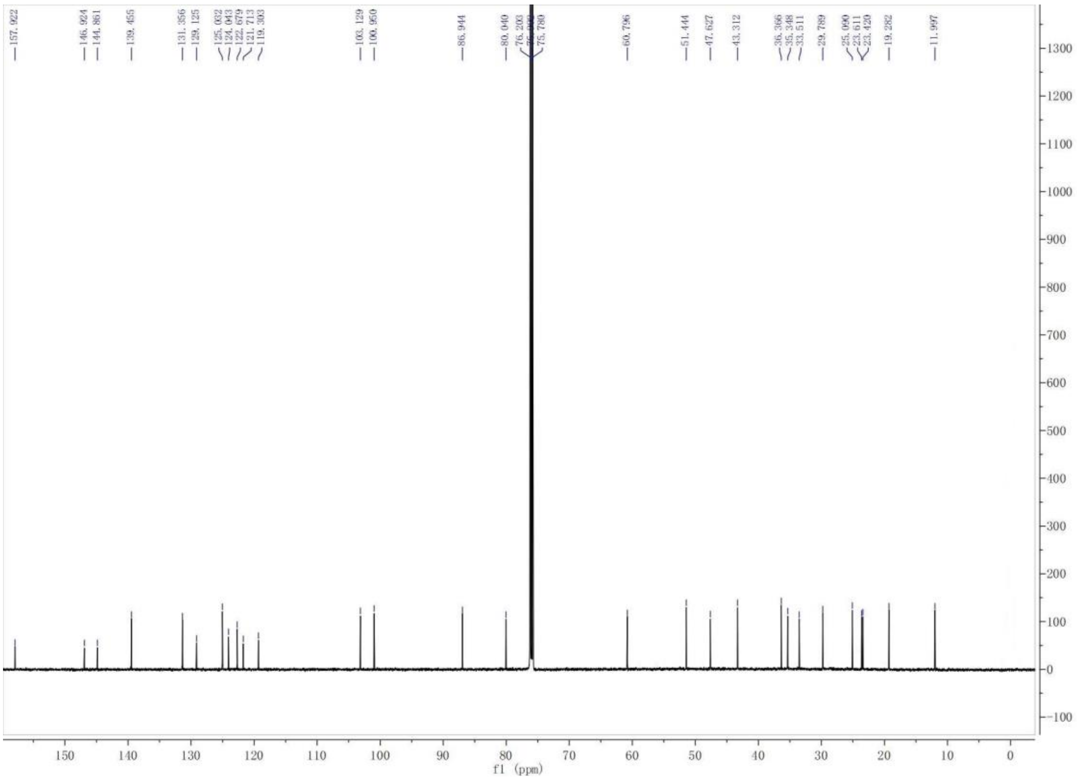

Compound **1e**

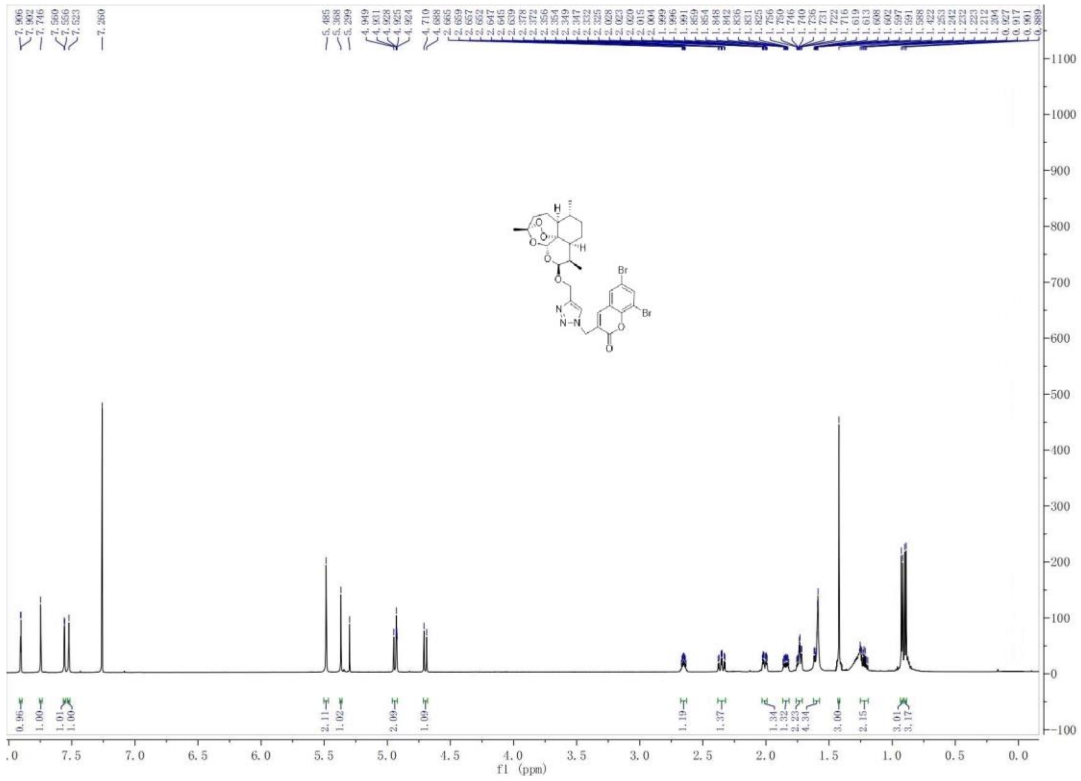

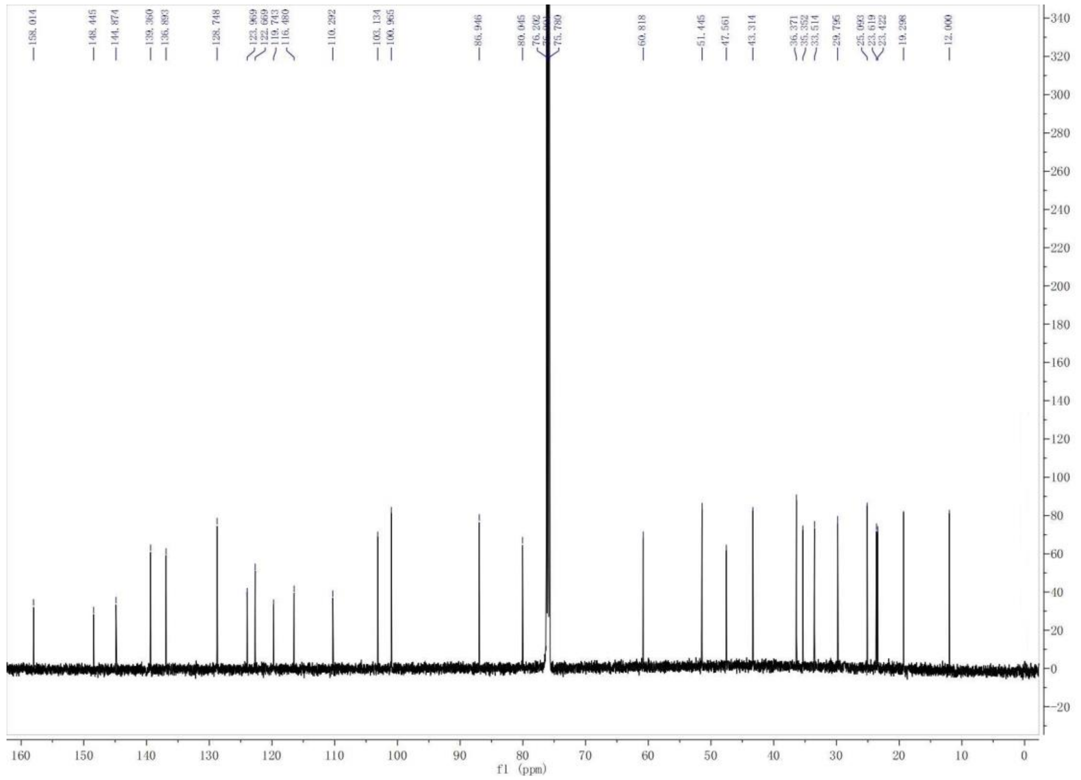

Compound **2a**

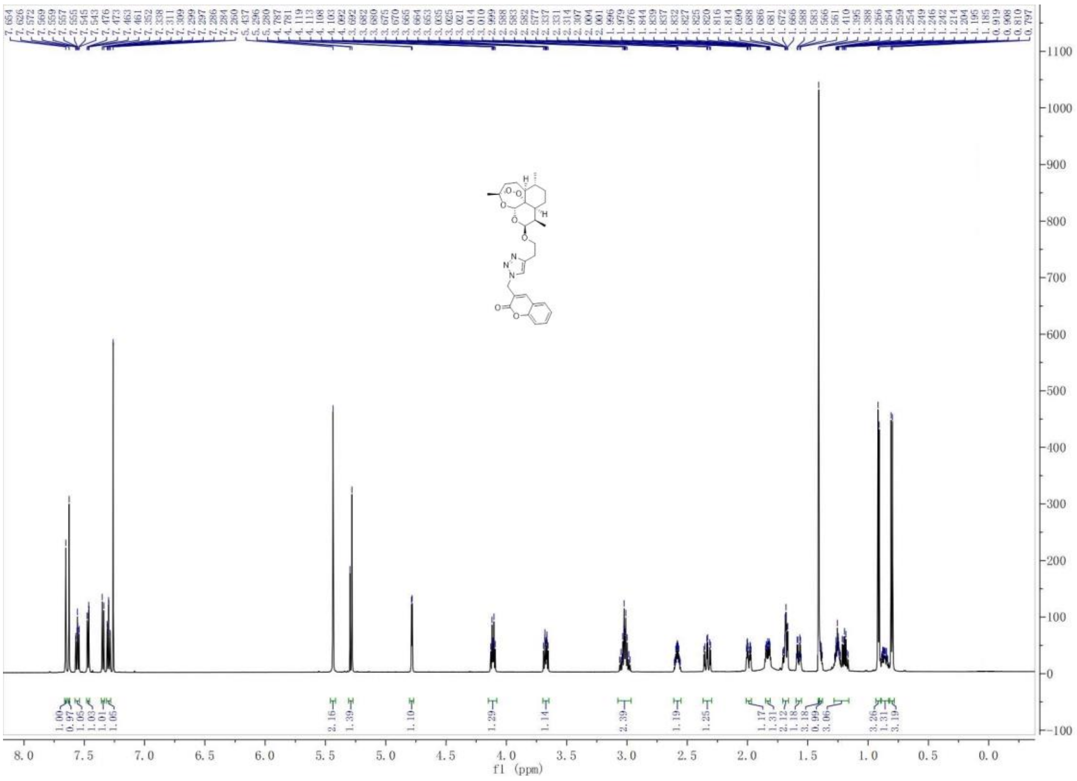

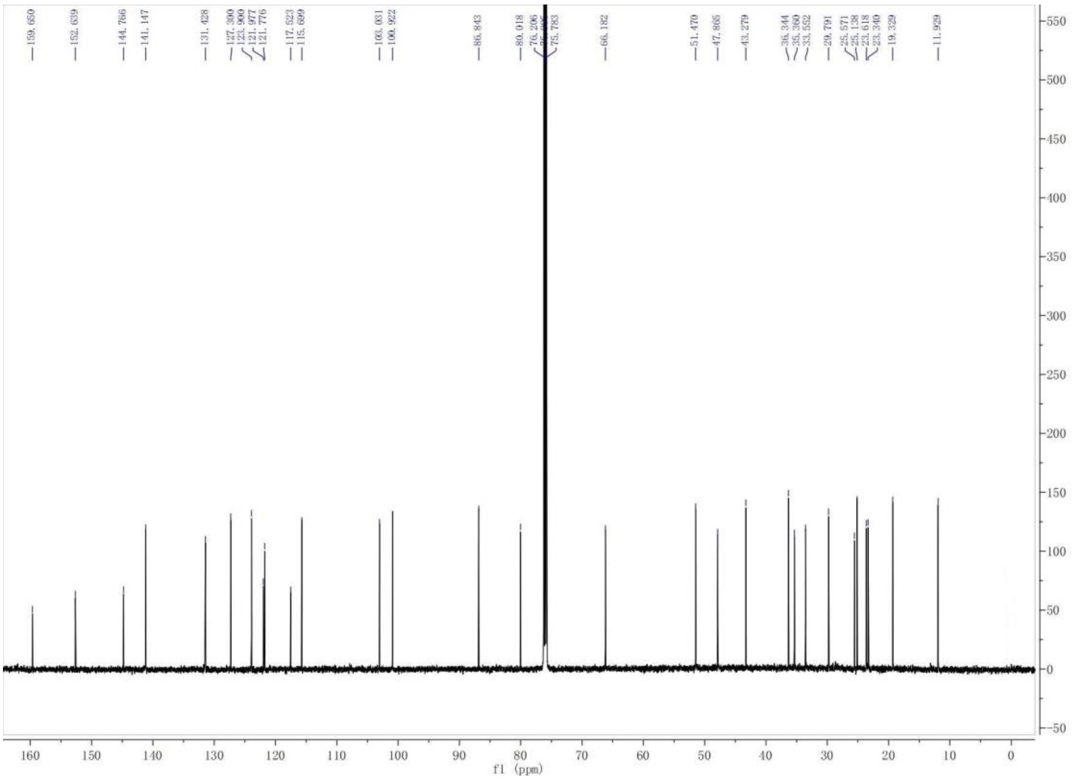

Compound **2b**

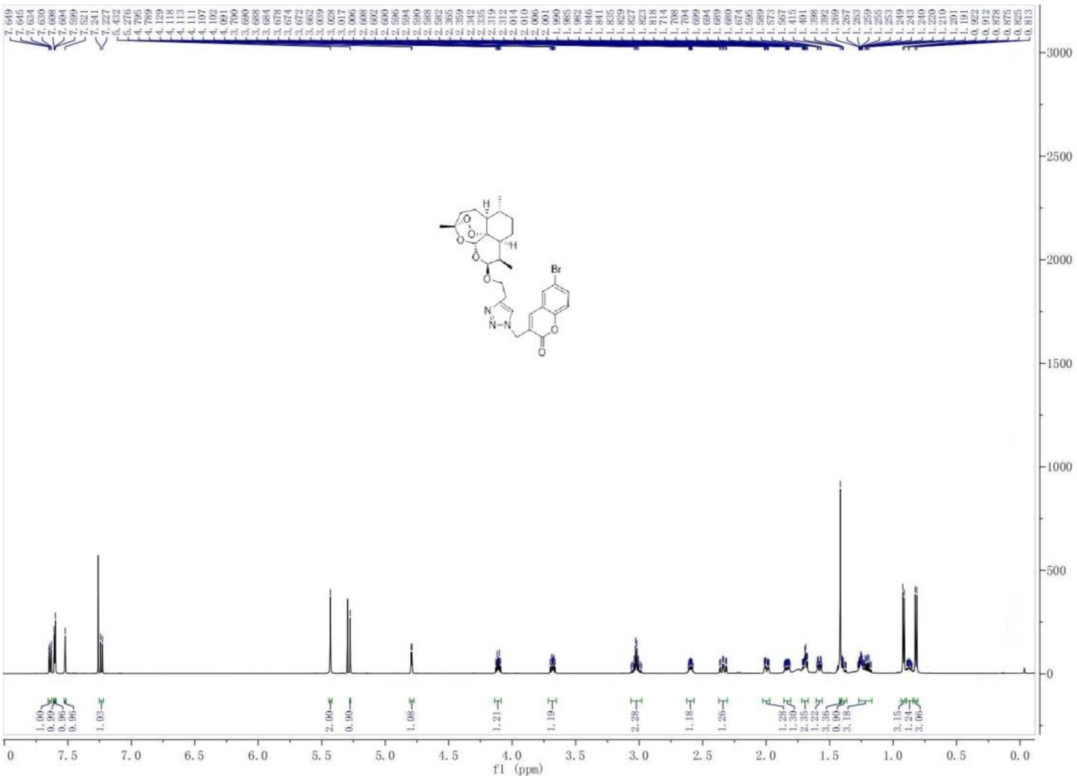

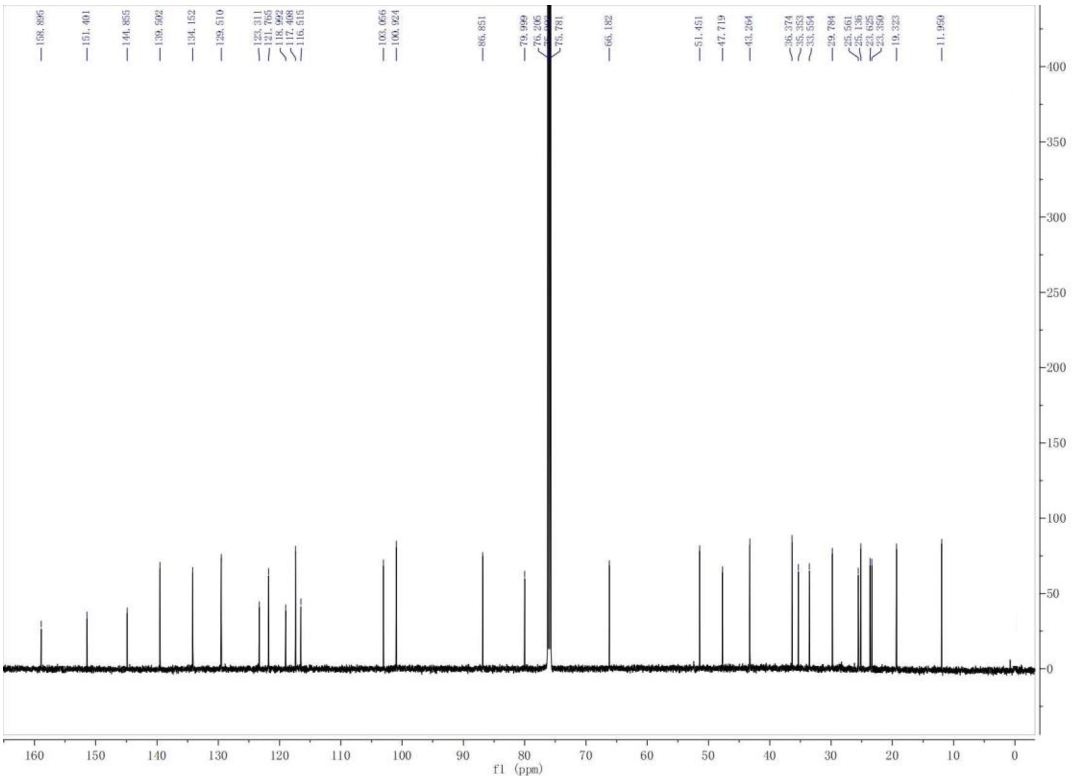

Compound **2c**

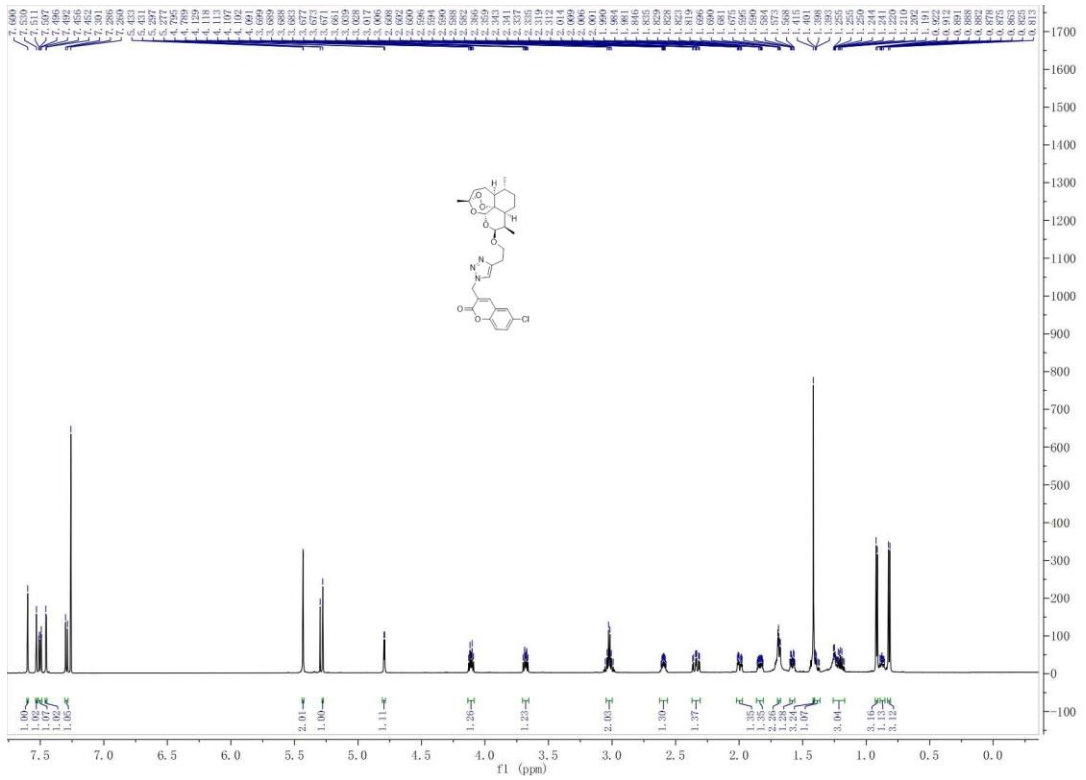

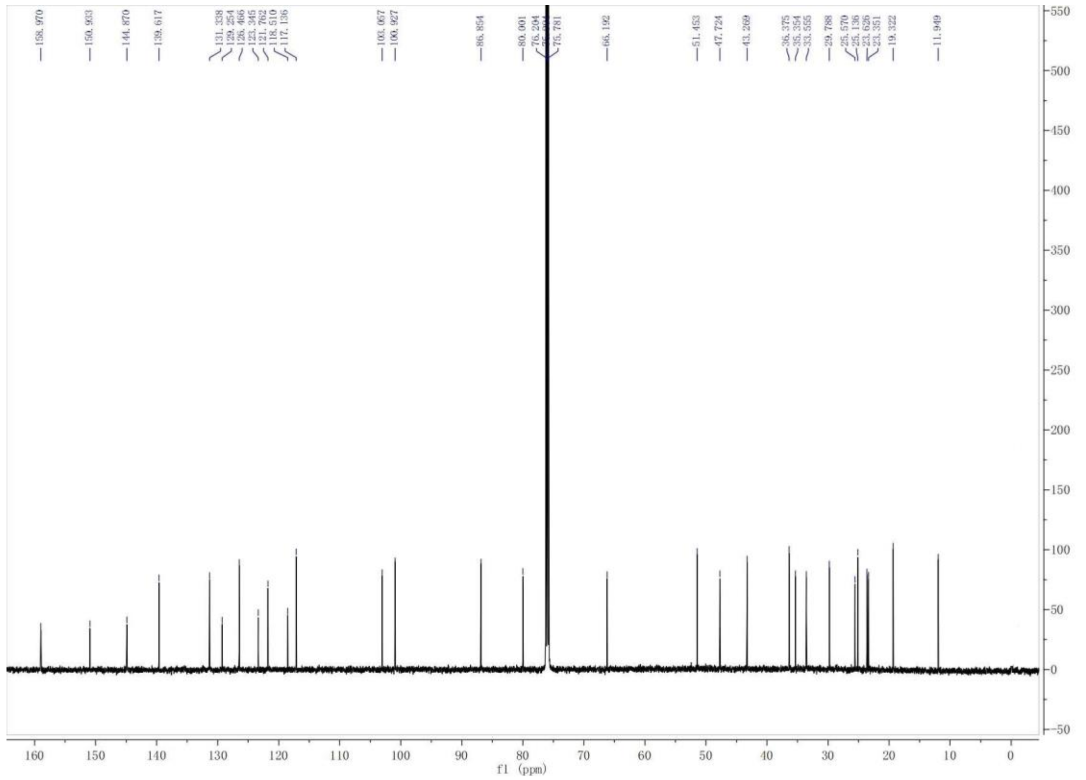

Compound **2d**

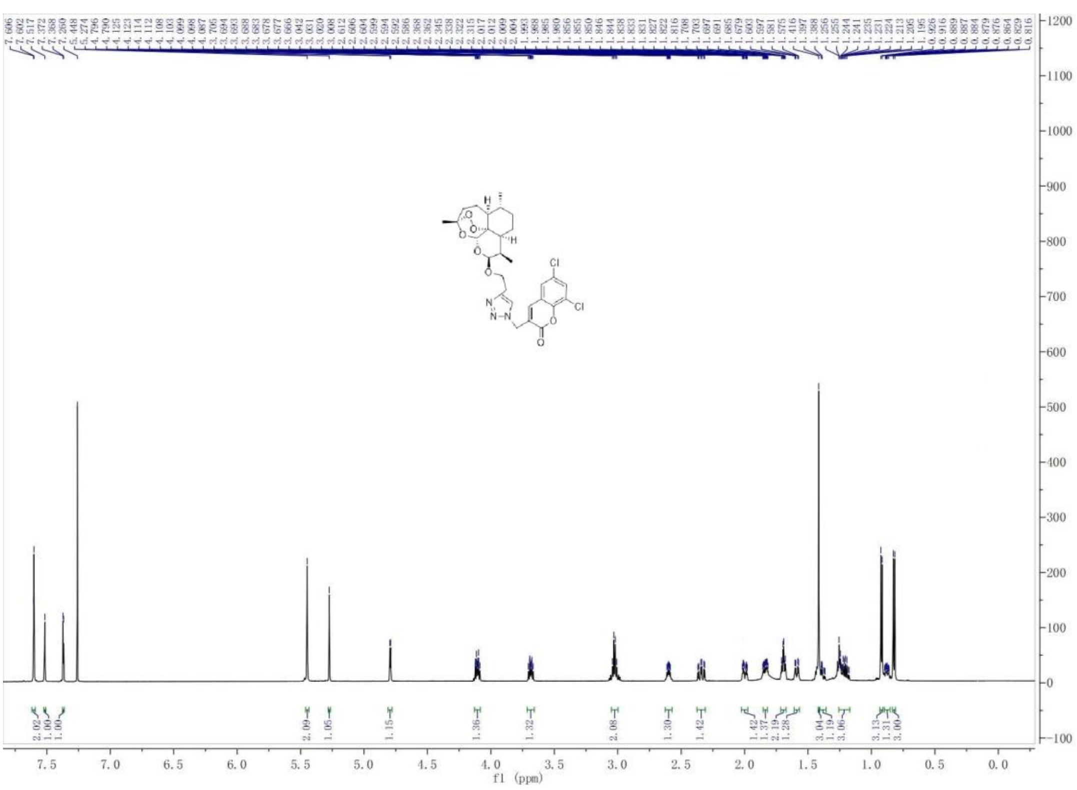

7.606 7.602 7.517 7.372 7.368 7.269 5.448 5.274 4.796 4.790
2.02 1.00 1.00 2.09 1.05 1.15 1.36 1.32 2.08 1.30 1.42 1.42 1.37 2.19 1.28 3.04 1.19 3.06 3.13 1.31 3.00
7.5 7.0 6.5 6.0 5.5 5.0 4.5 4.0 3.5 3.0 2.5 2.0 1.5 1.0 0.5 0.0
f1 (ppm)
Cl
Cl
N
N
N
O
O
O
O
O
H
H

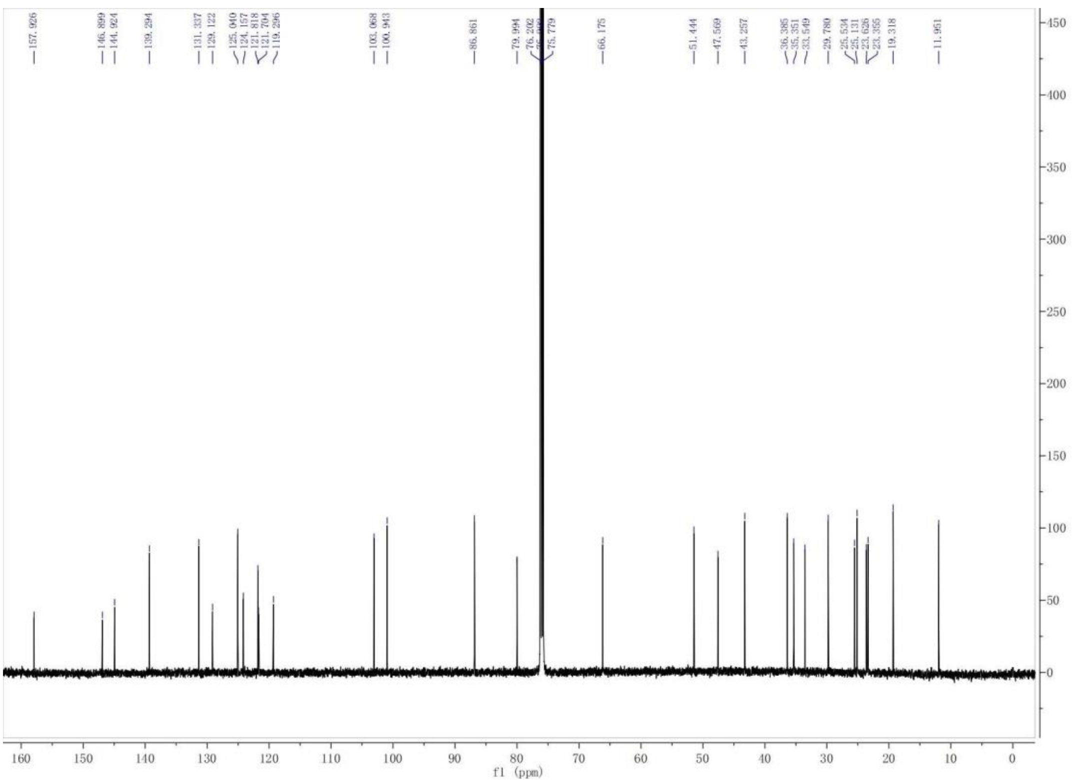

157.926 146.899 144.924 139.294 131.337 129.122 125.040 124.157 121.818 121.704 119.296 103.068 100.943 86.861 79.994 76.202 75.779 66.175 51.444 47.569 43.257 36.385 35.351 33.549 29.780 25.534 25.131 23.626 23.355 19.318 11.951
160 150 140 130 120 110 100 90 80 70 60 50 40 30 20 10 0
f1 (ppm)

Compound **2e**

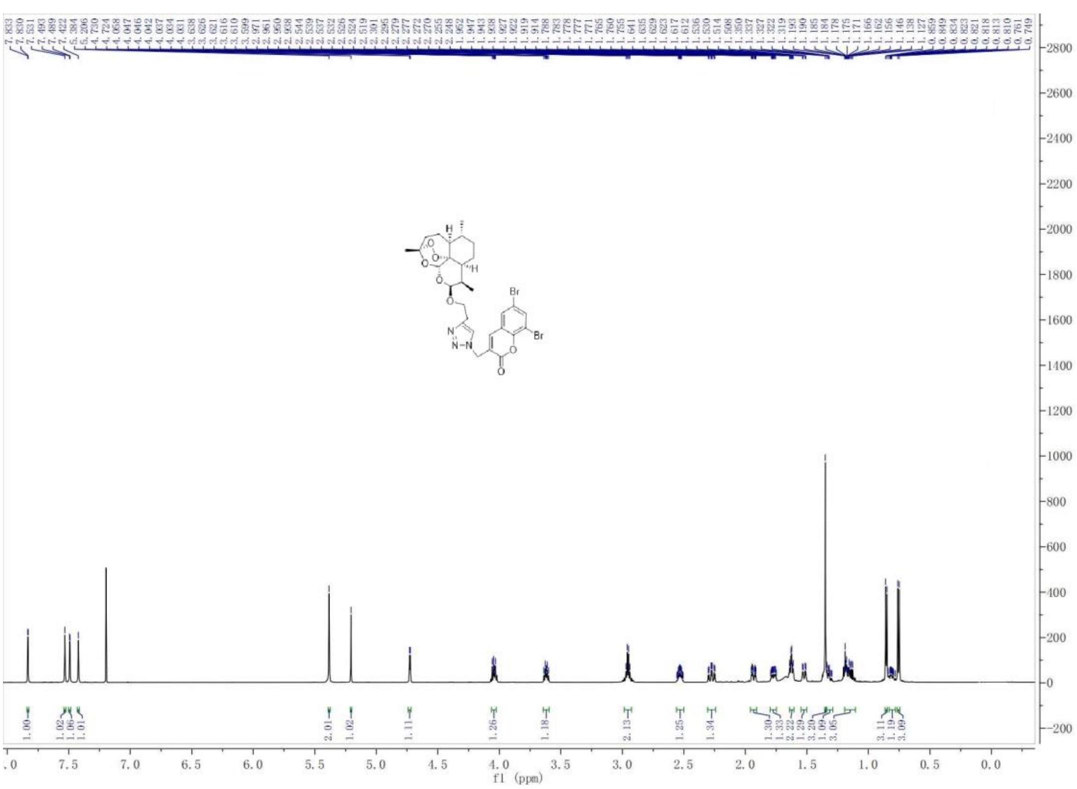

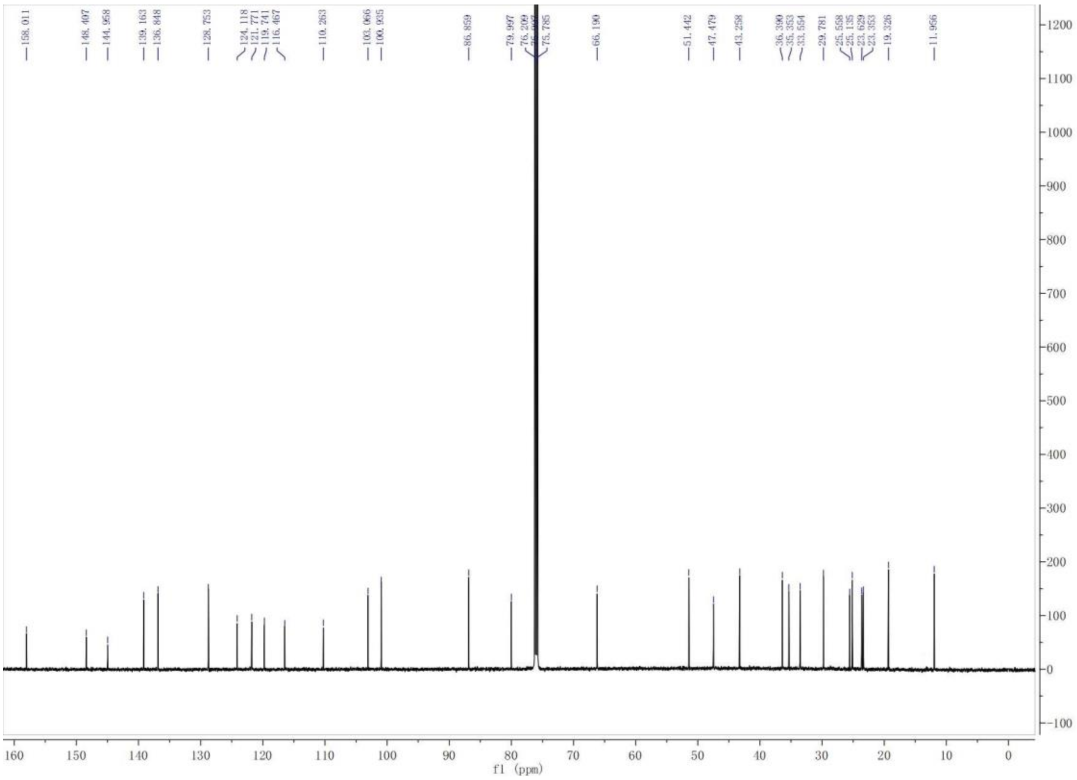

Compound **3f**

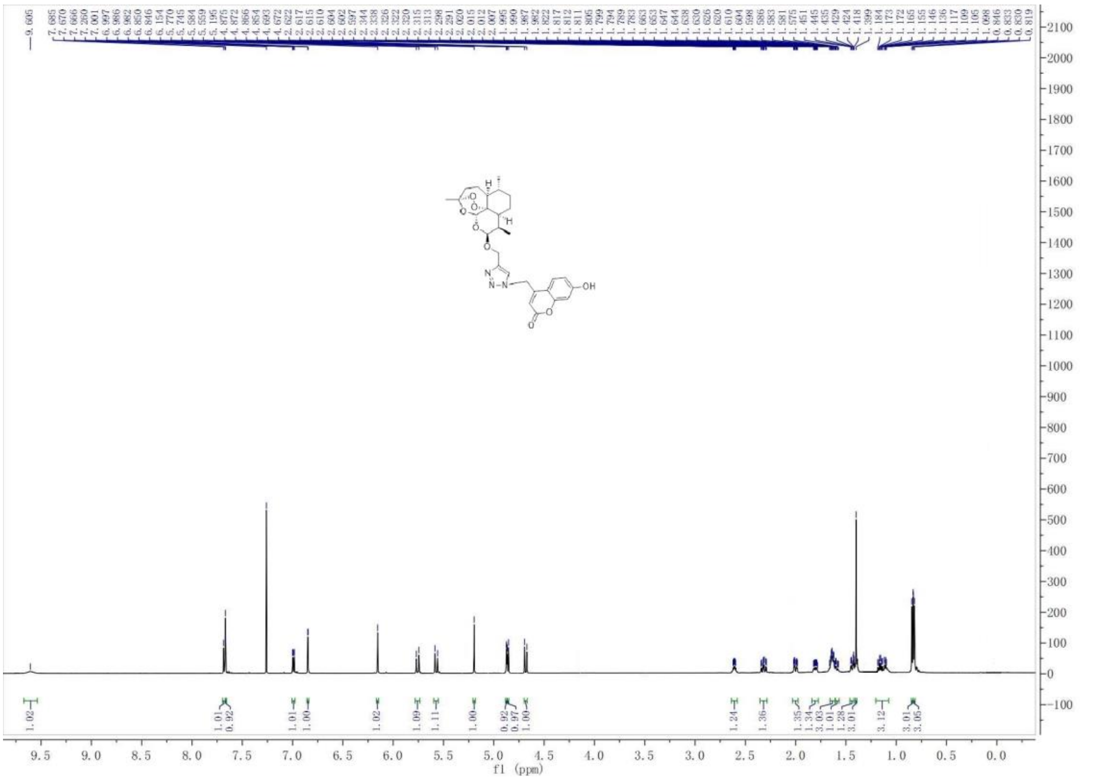

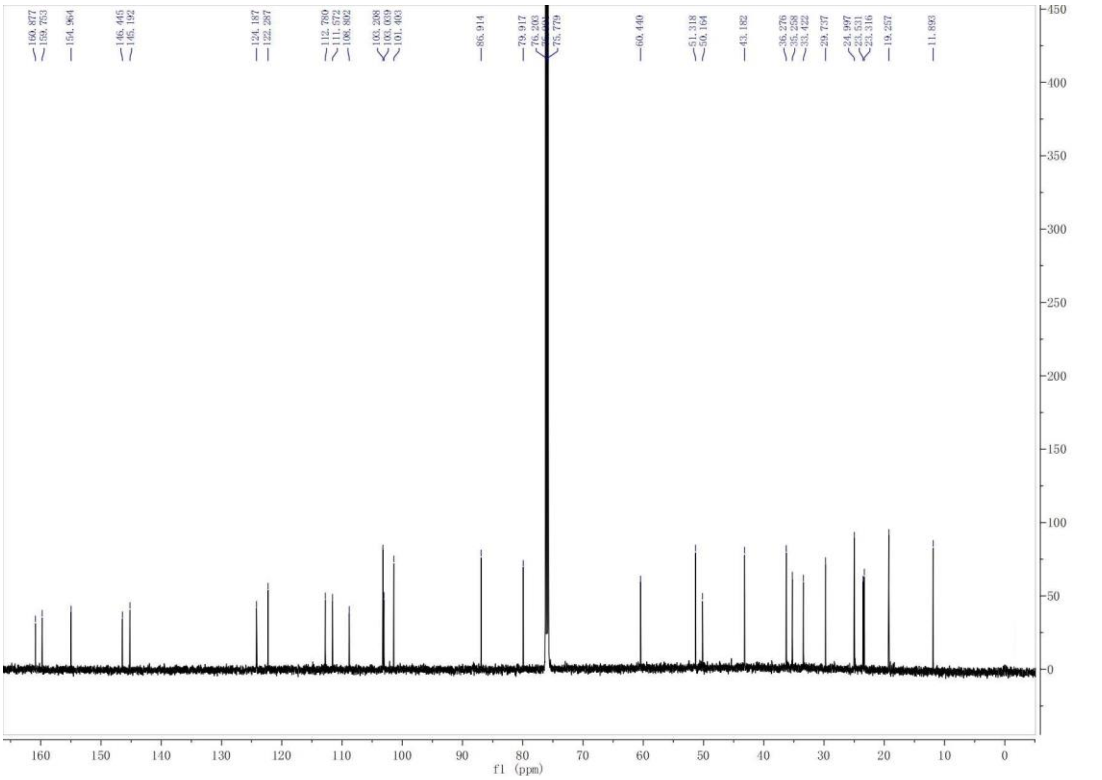

Compound **3g**

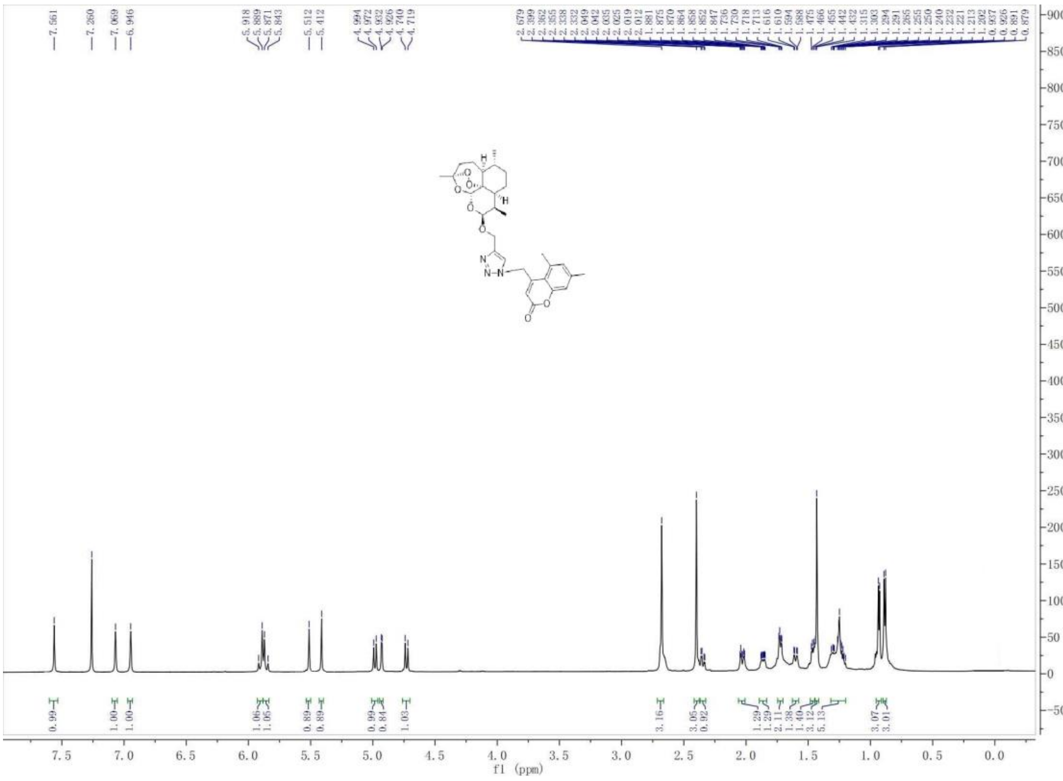

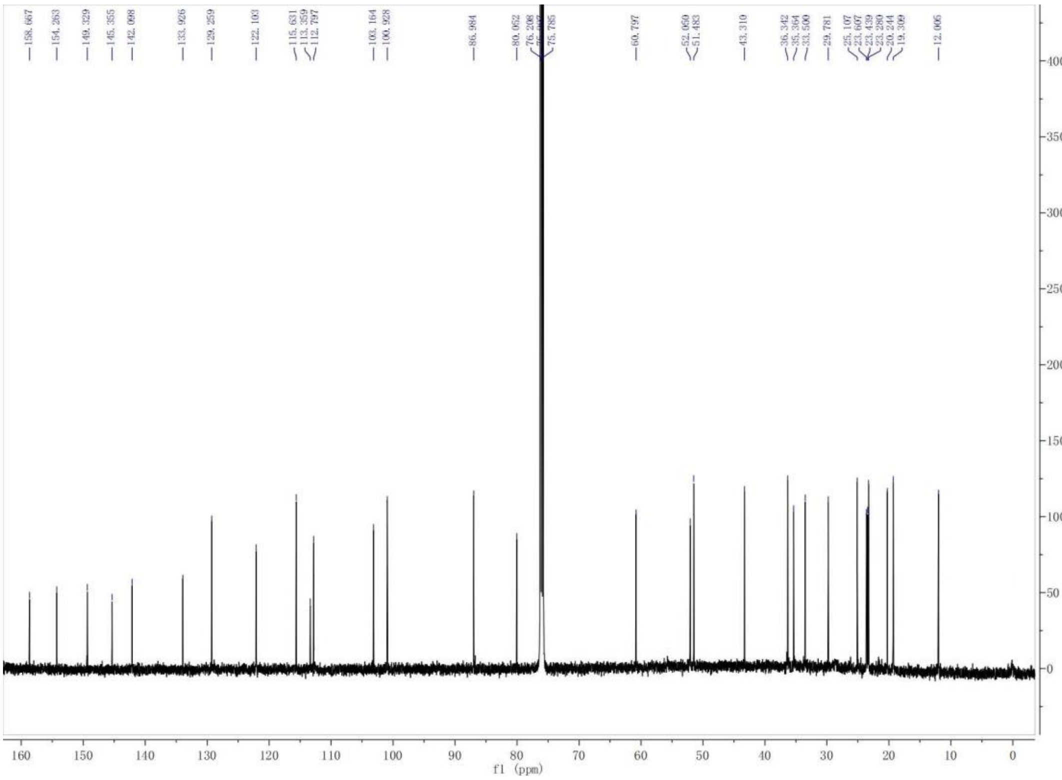

Compound **3h**

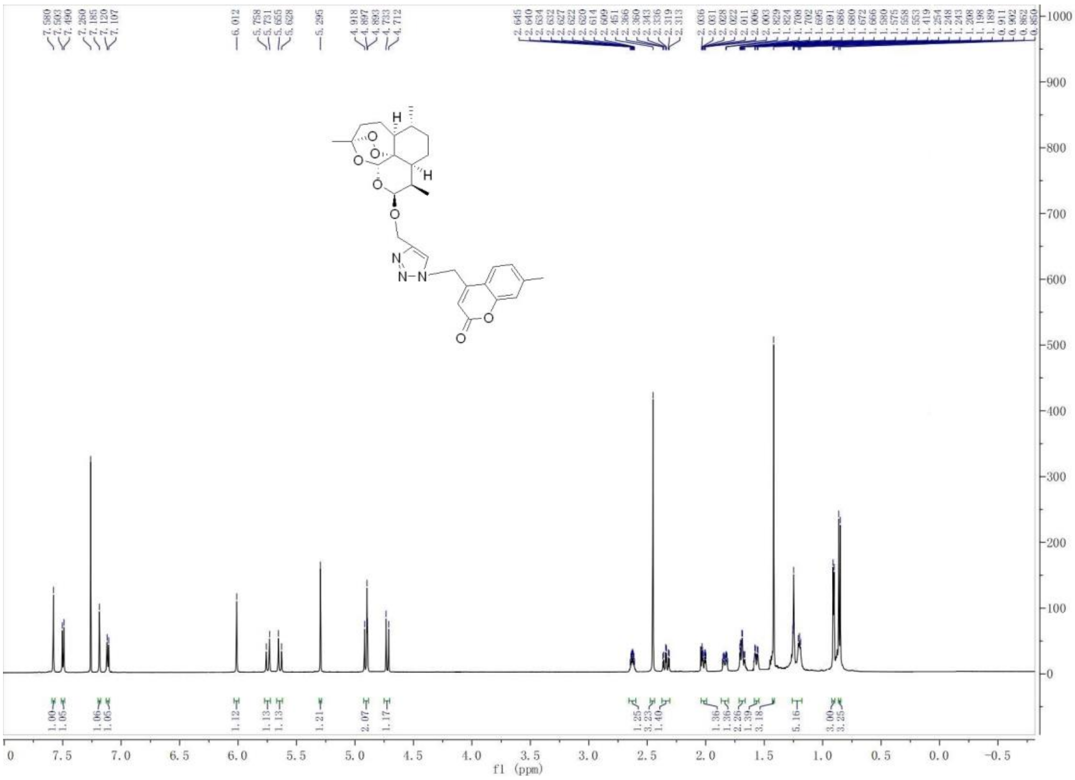

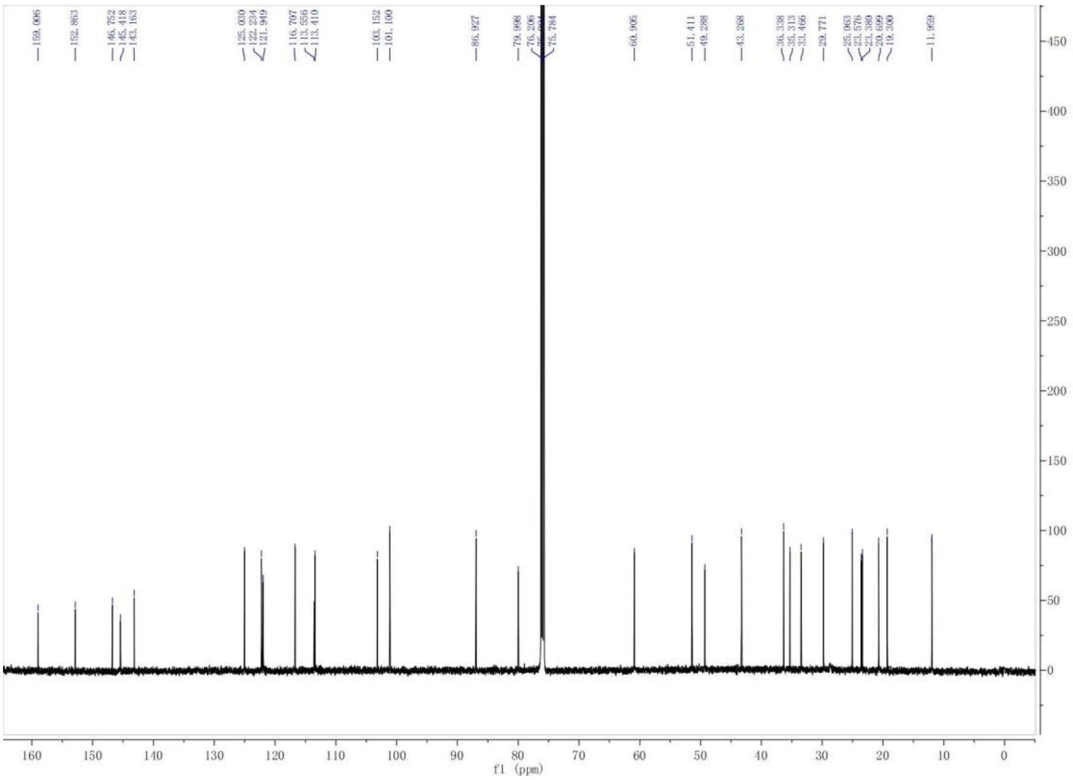

Compound **3i**

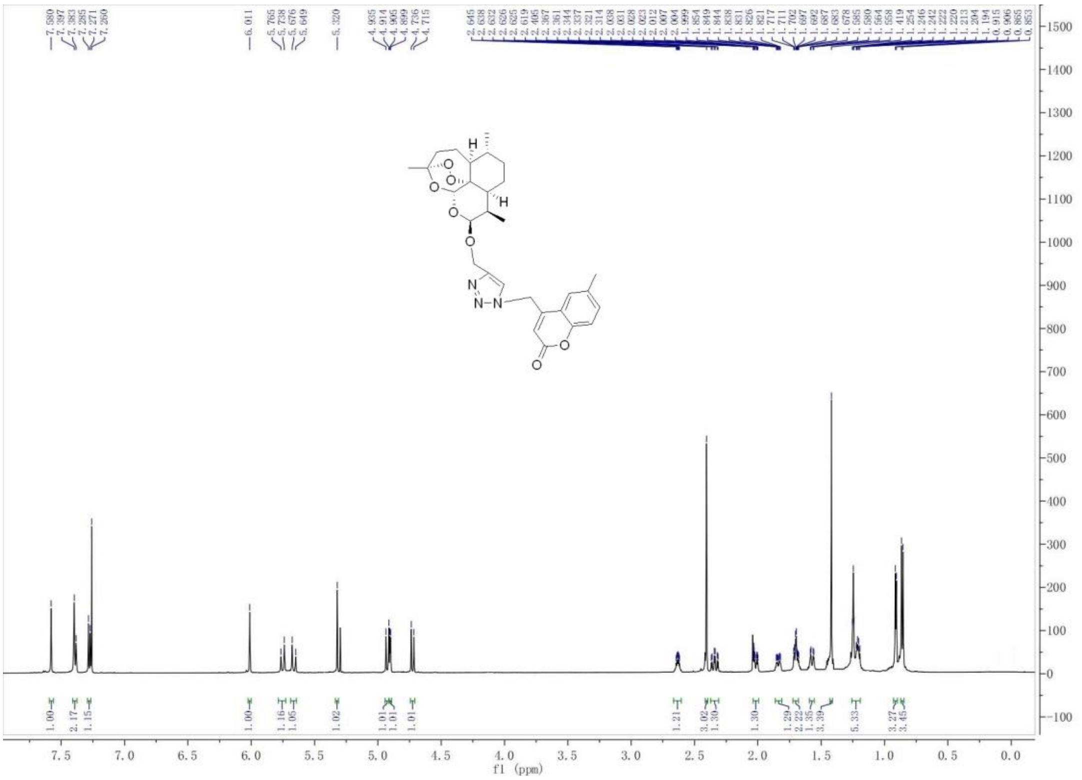

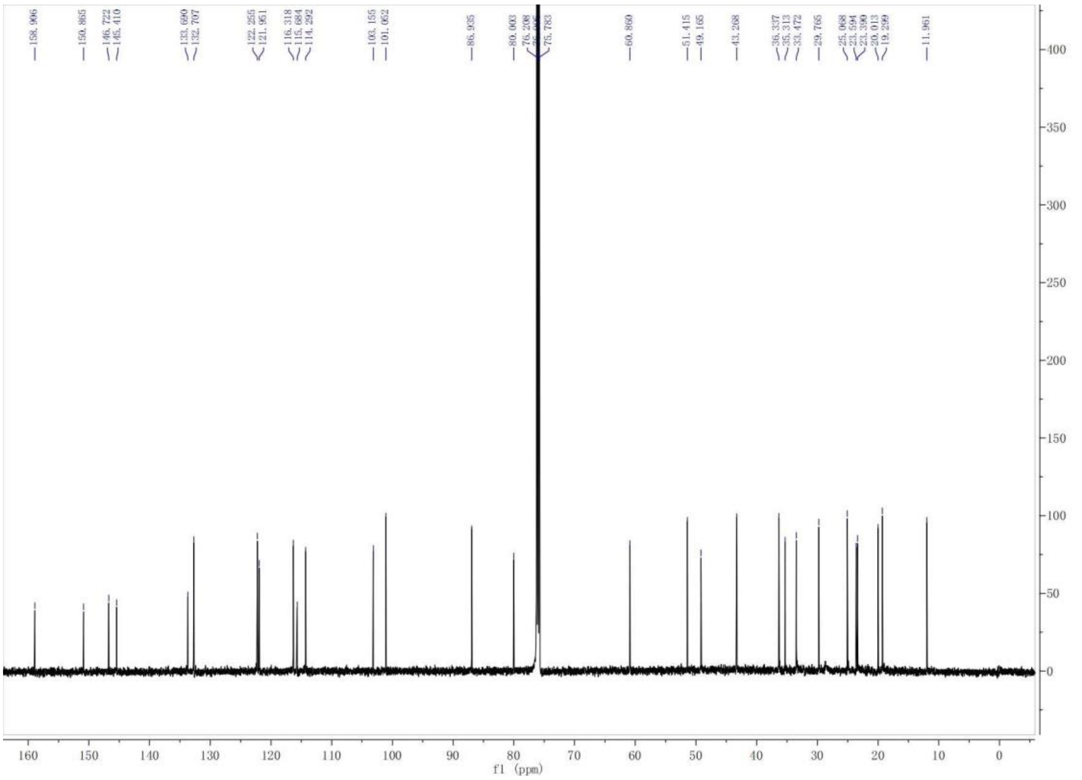

Compound **4f**

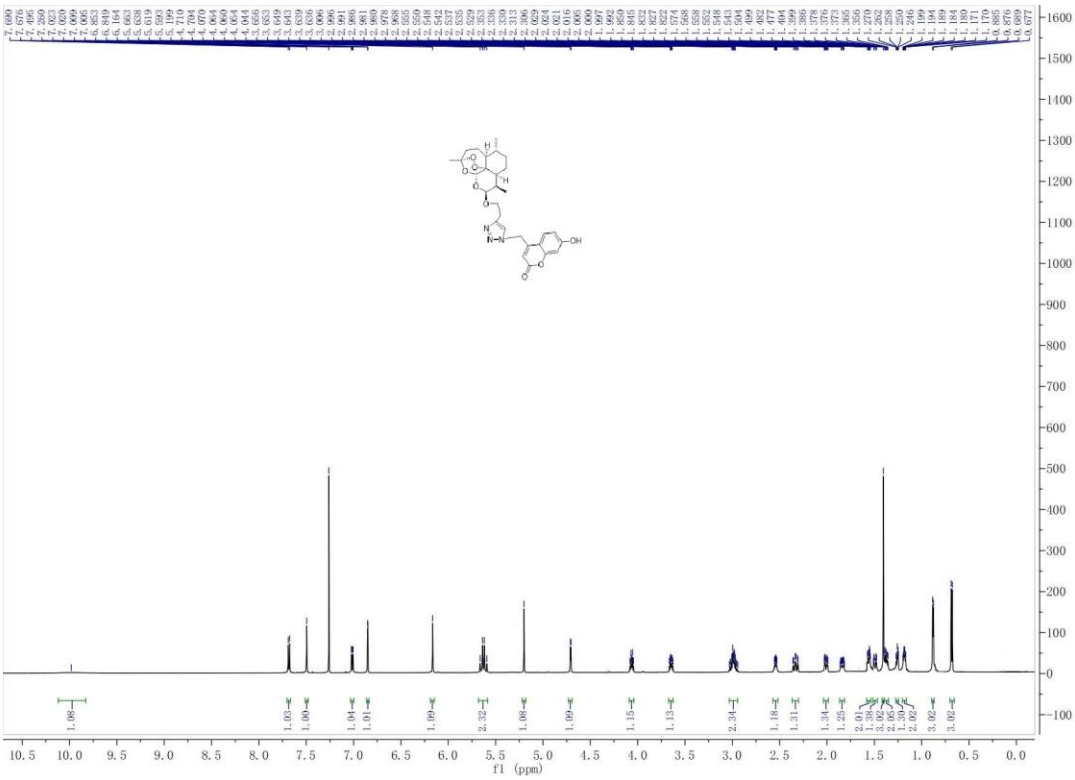

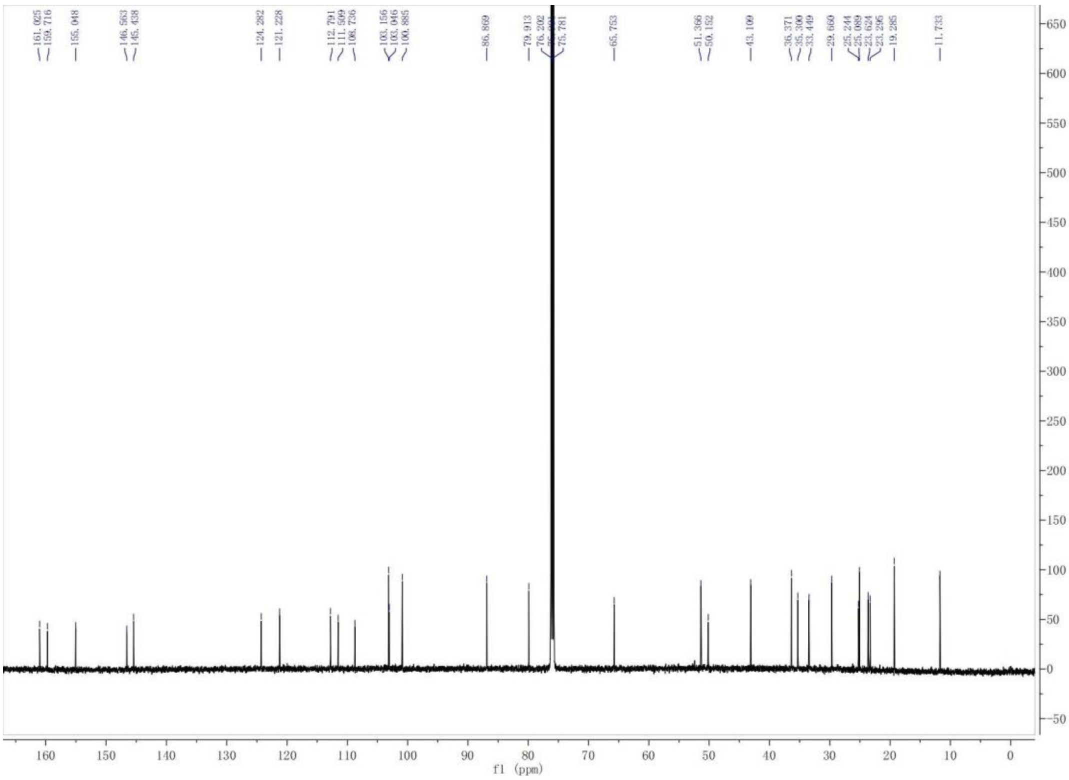

Compound **4g**

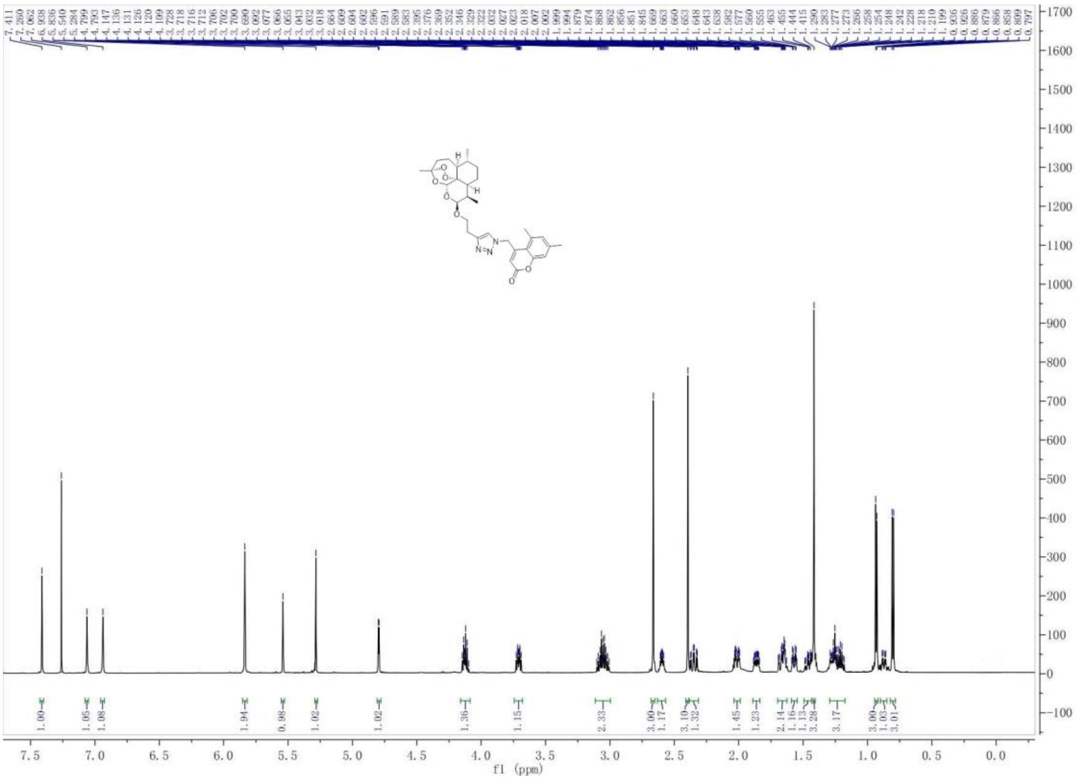

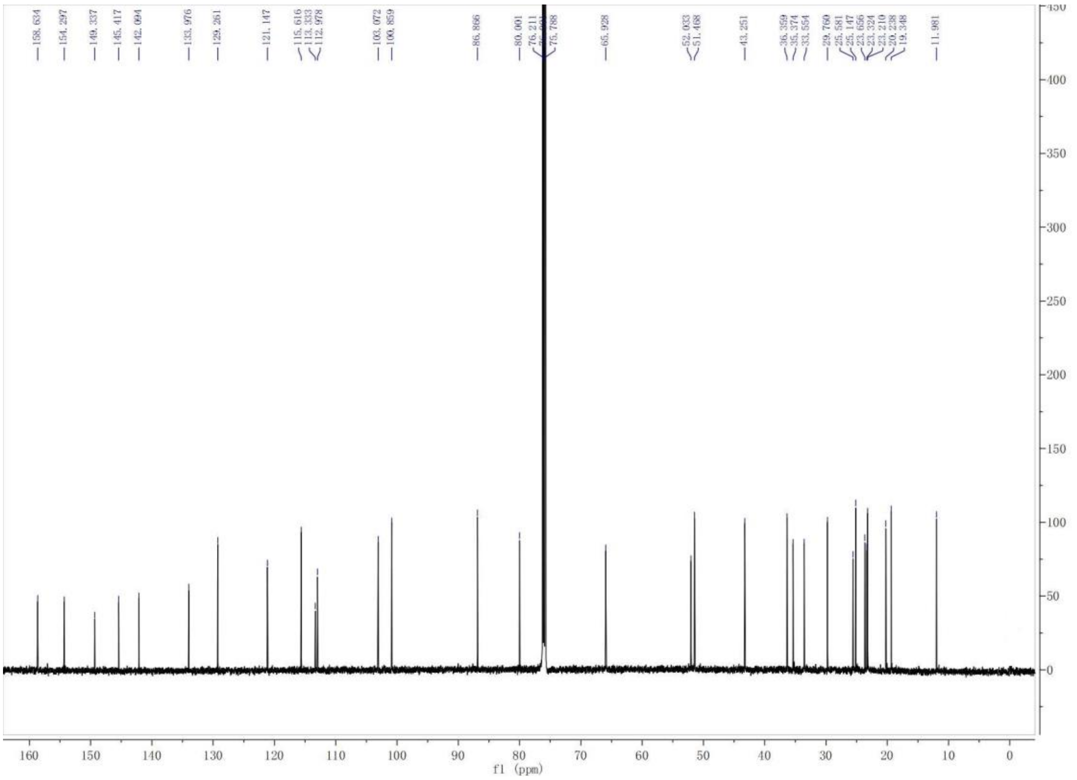

Compound **4h**

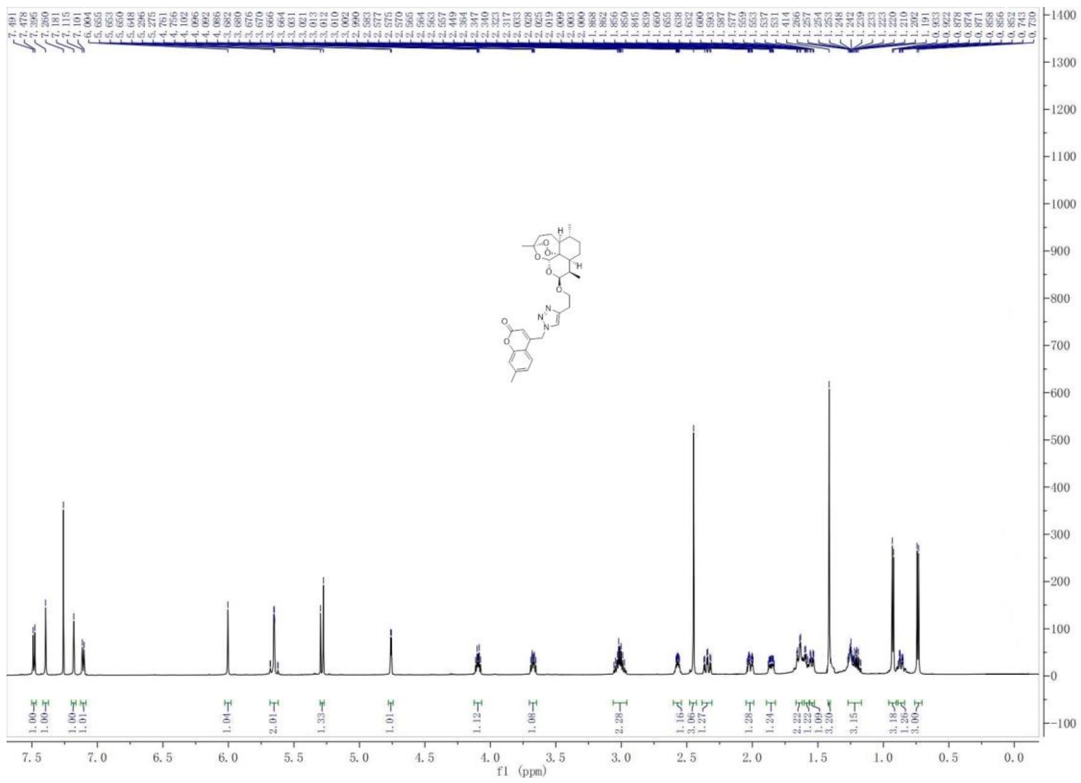

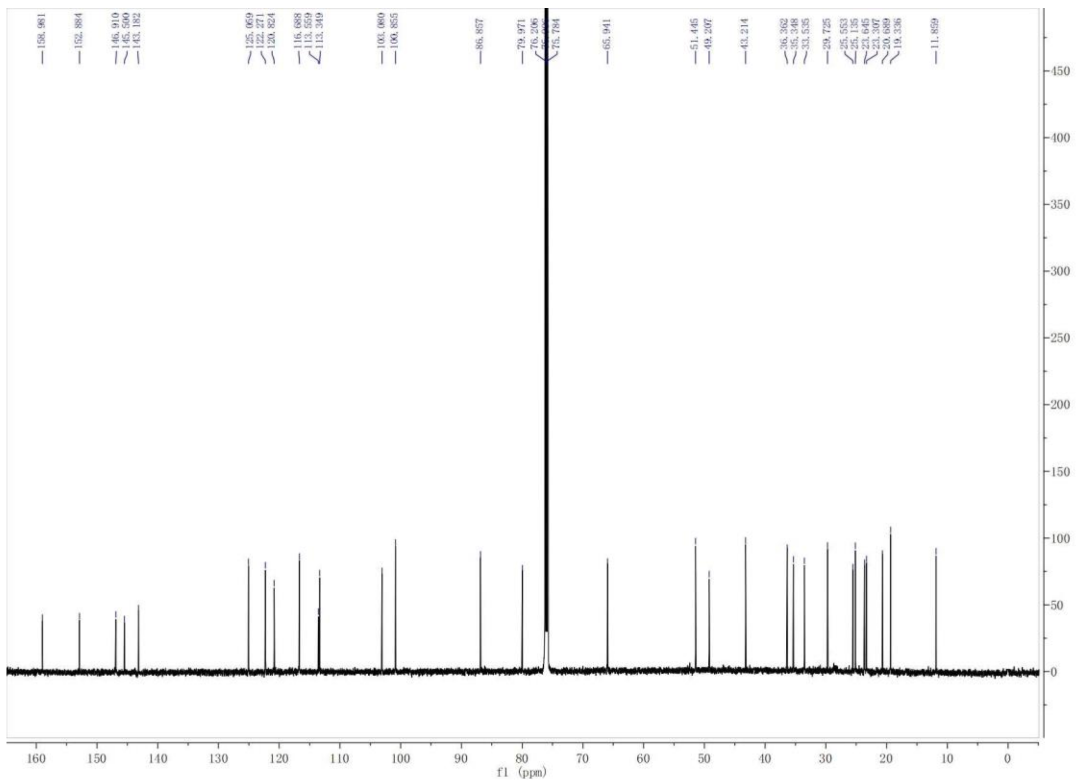

Compound **4i**

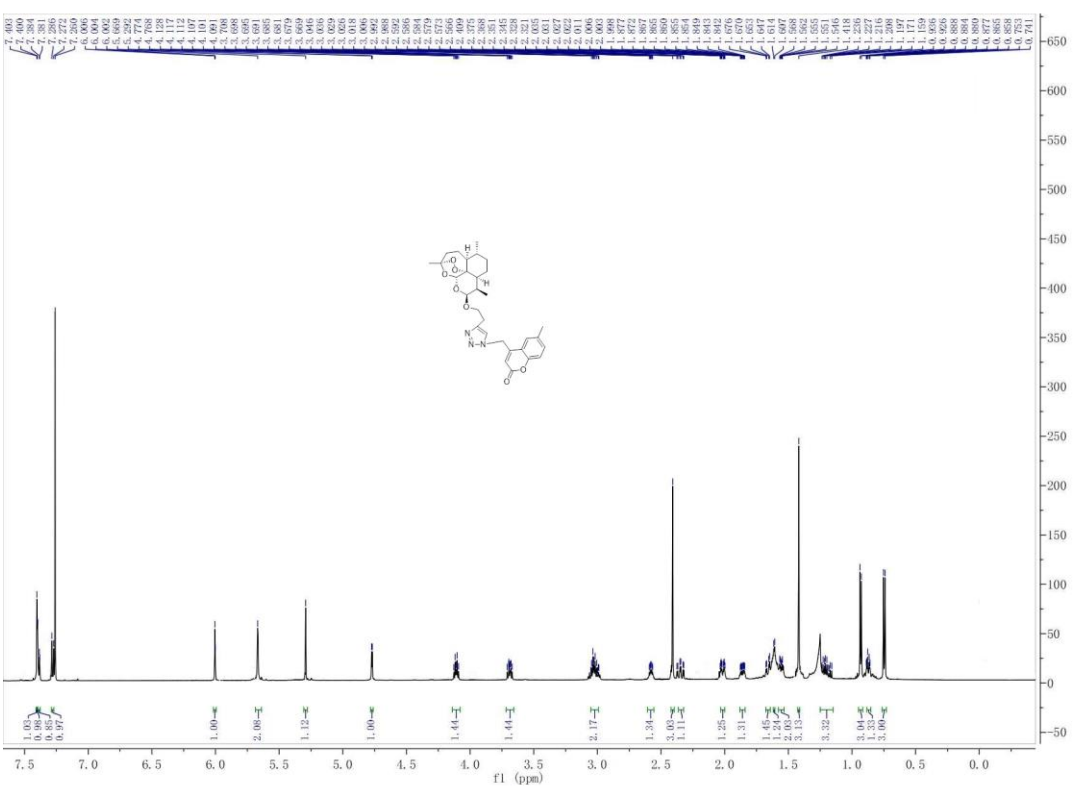

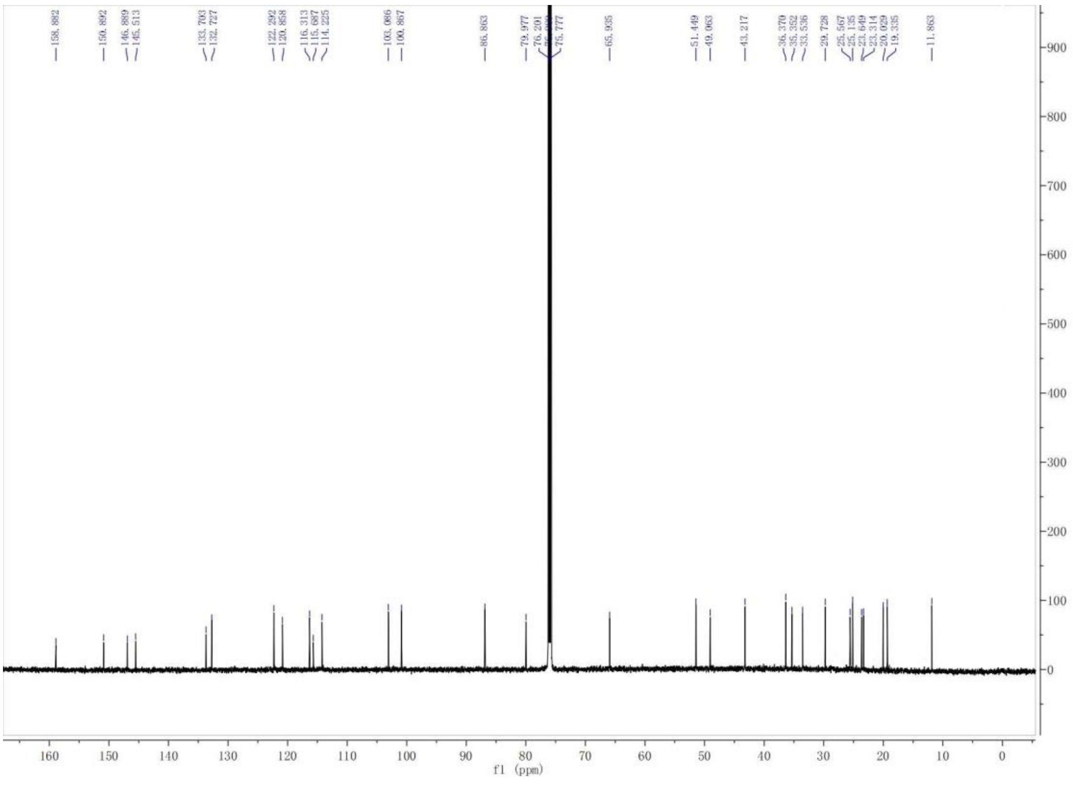

Compound **5j**

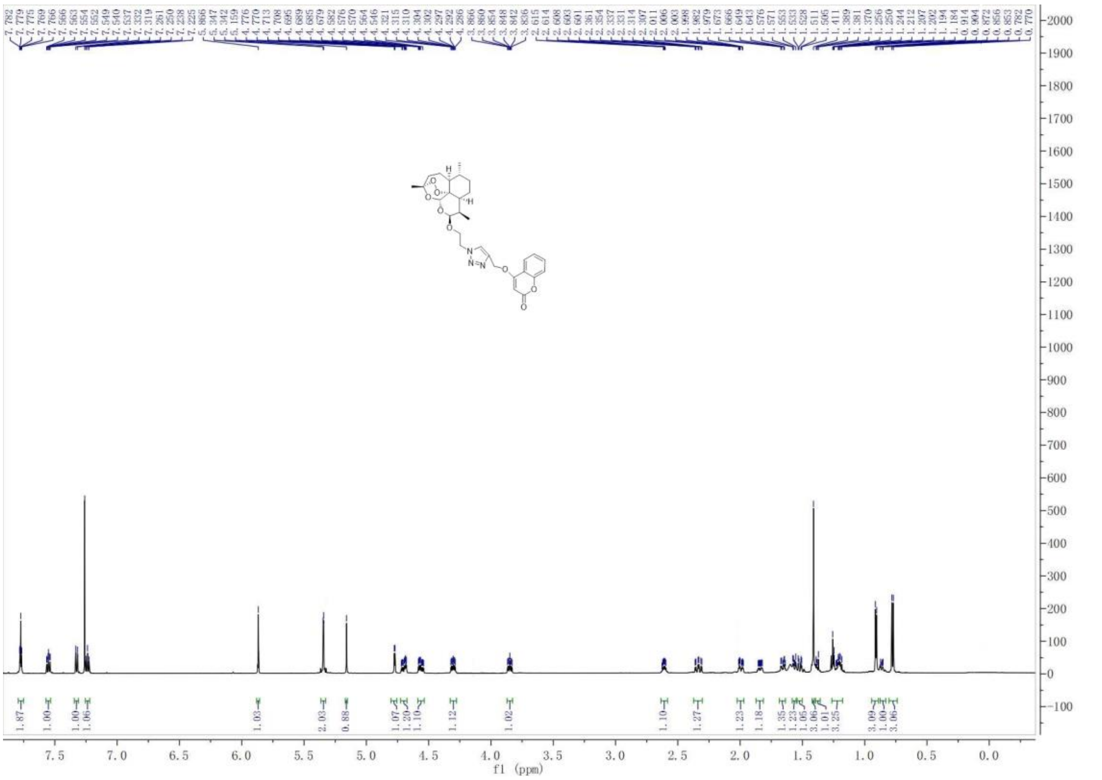

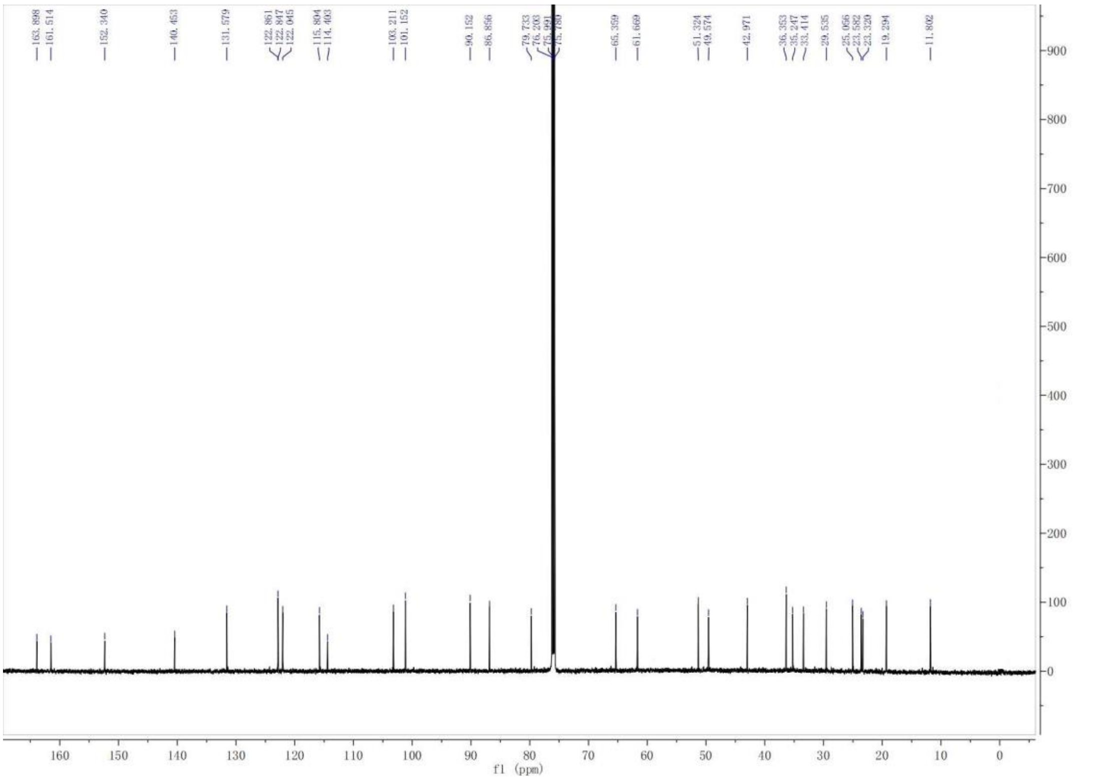

Compound **5l**

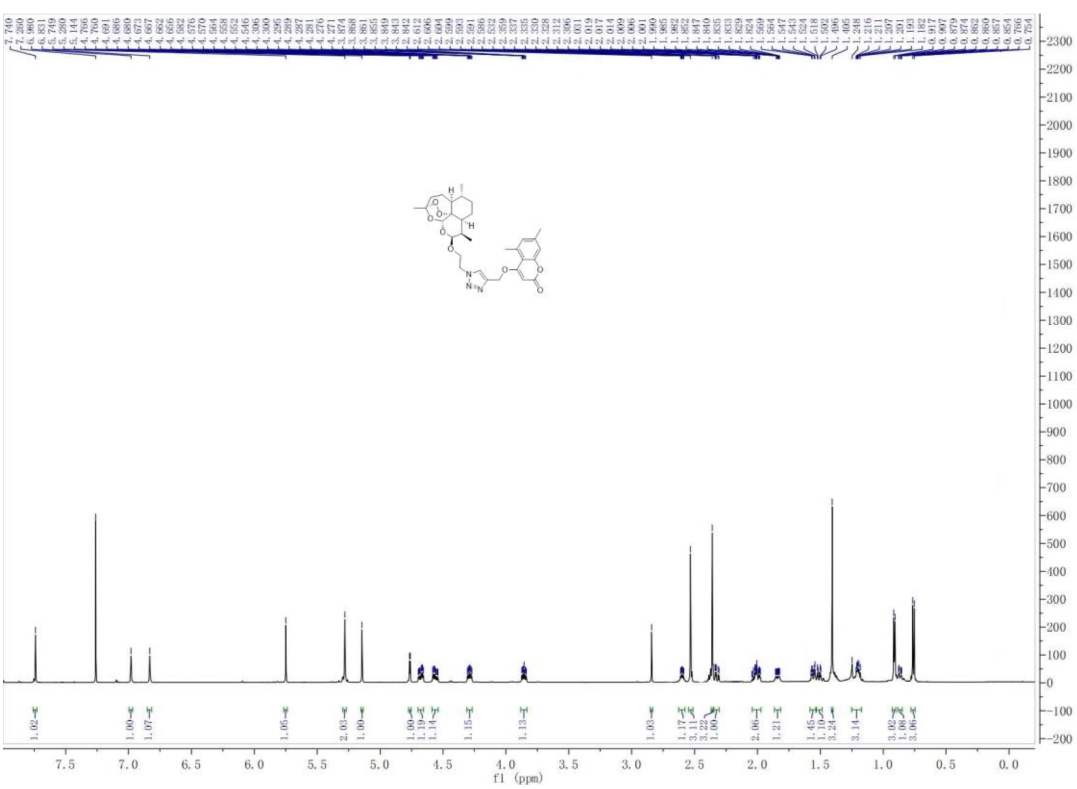

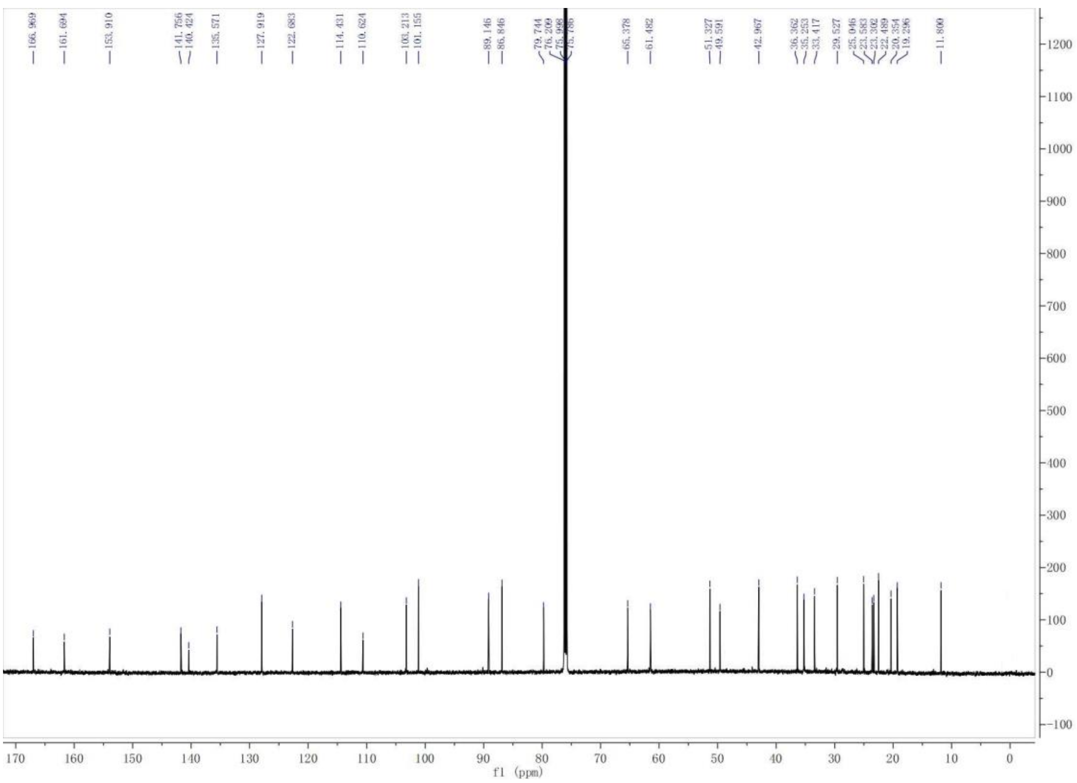

Compound **5m**

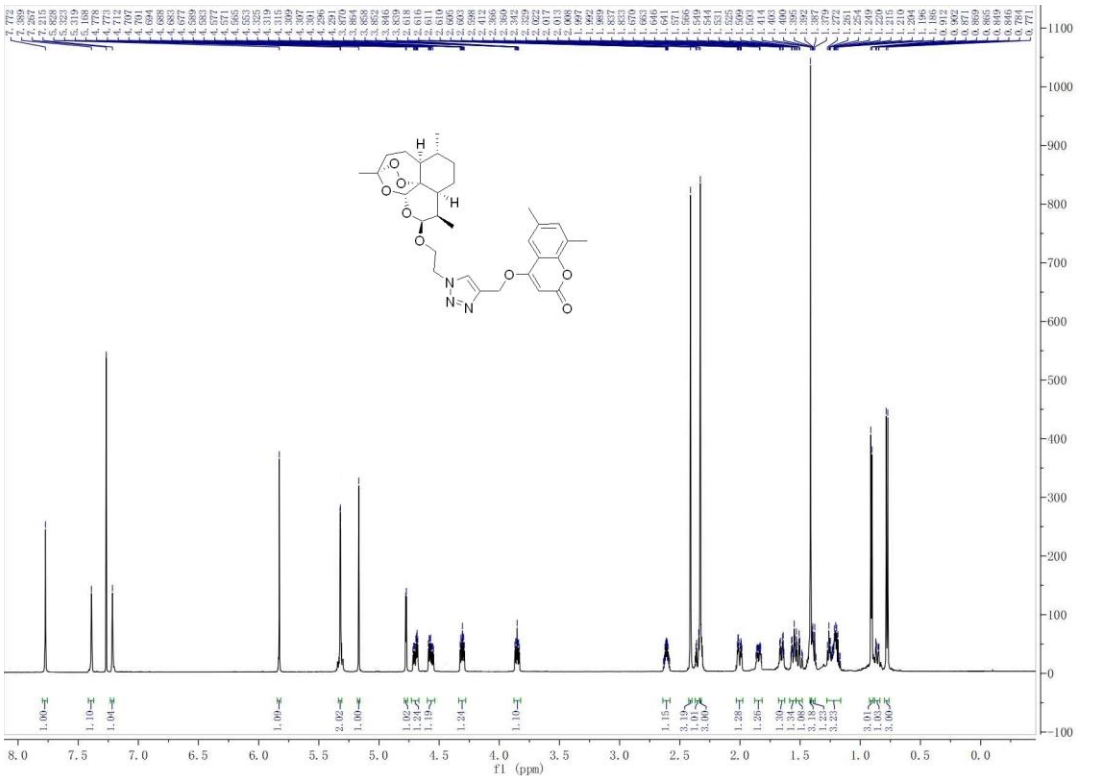

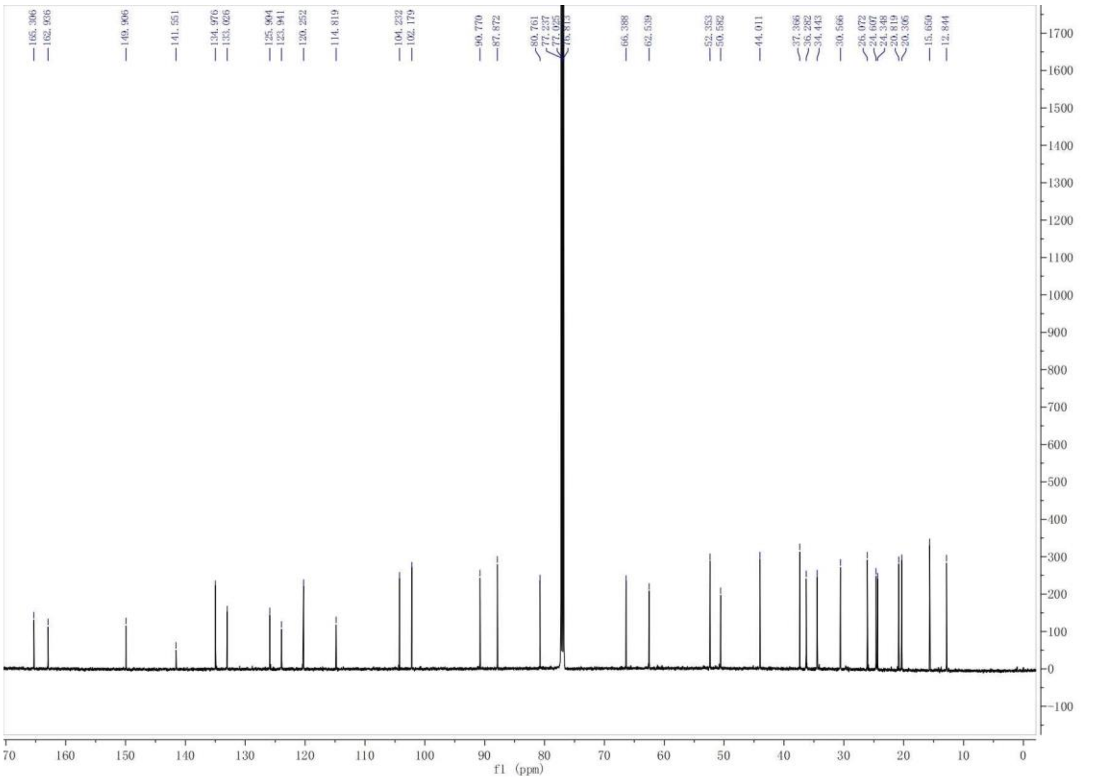

Compound **5n**

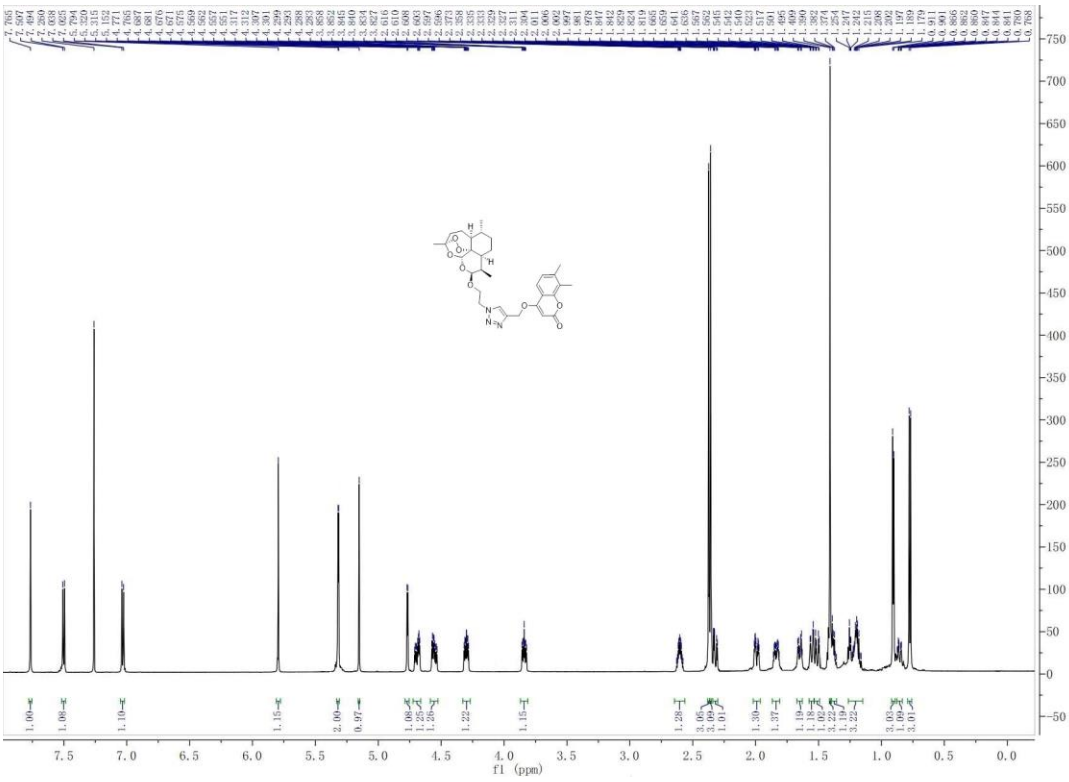

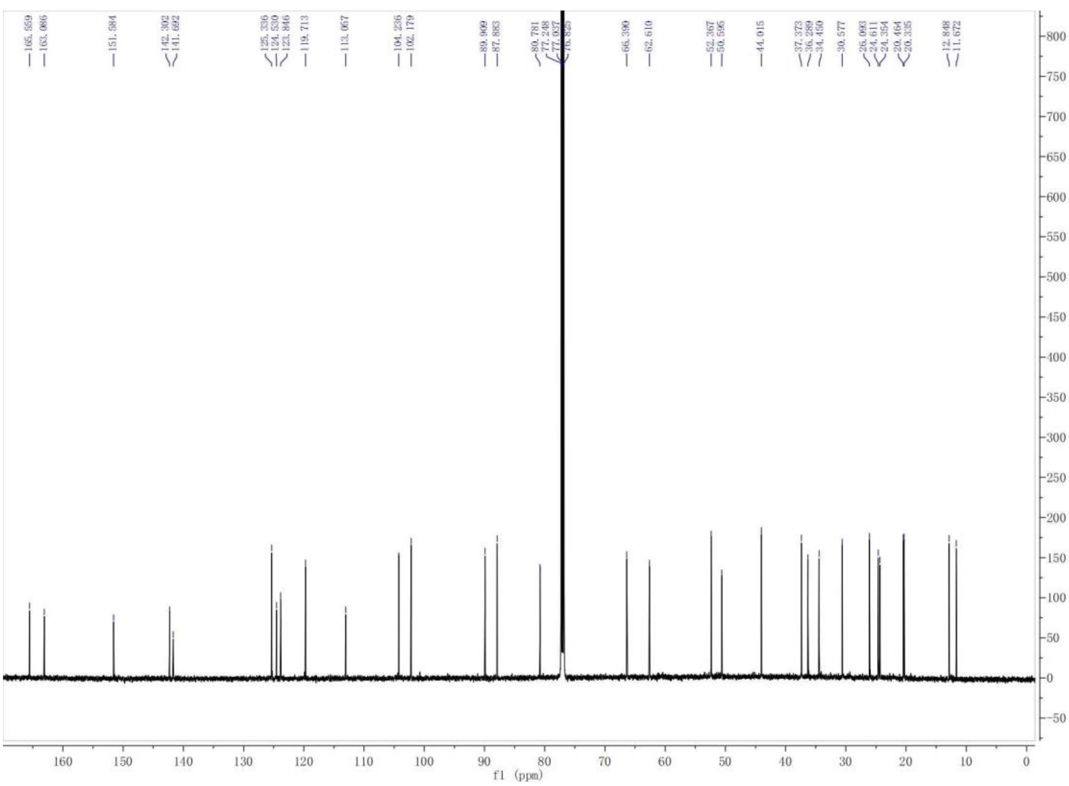

Compound **5o**

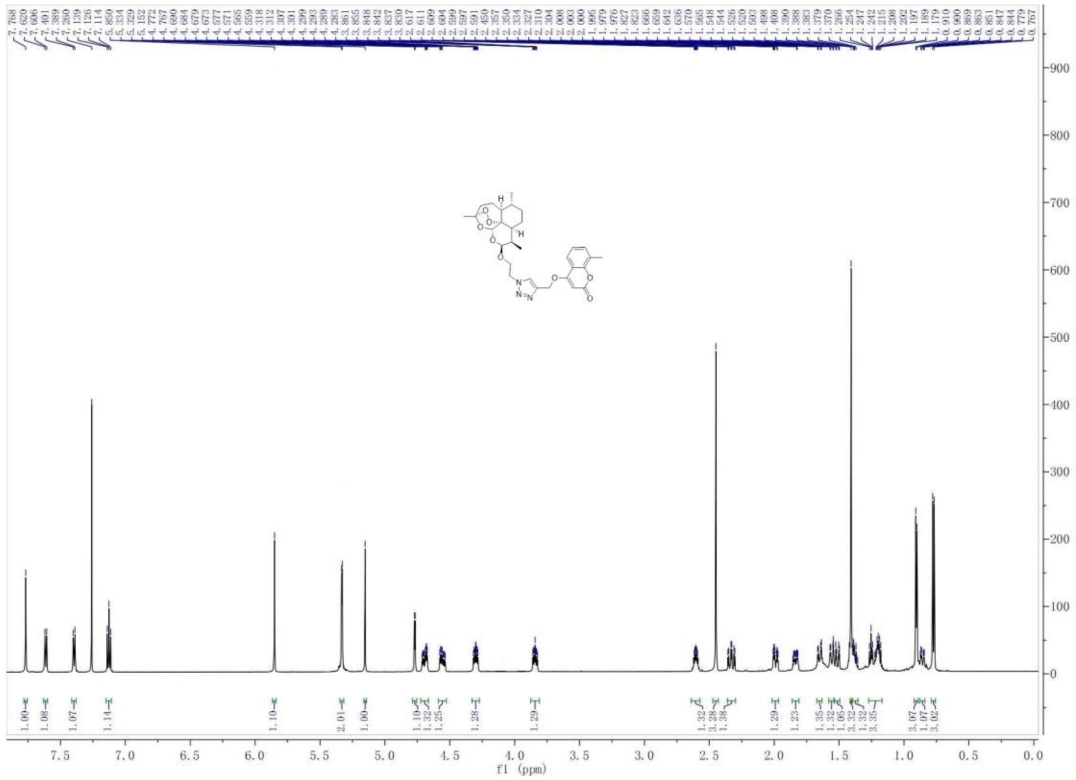

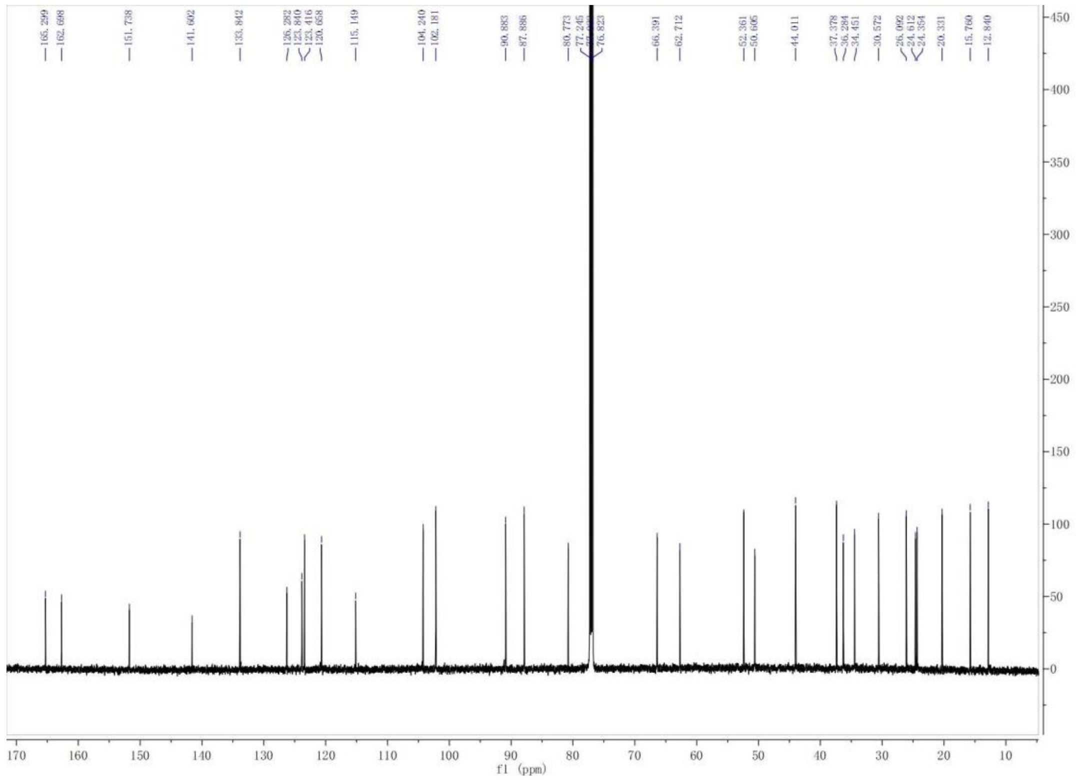

Compound **6j**

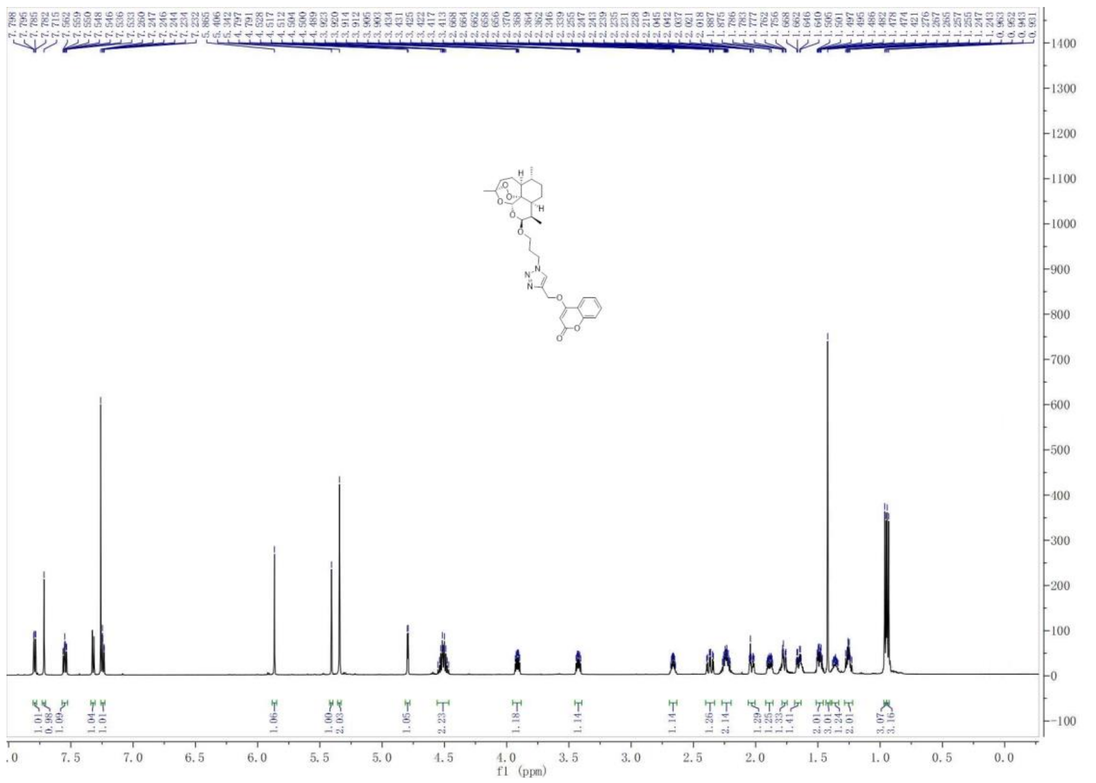

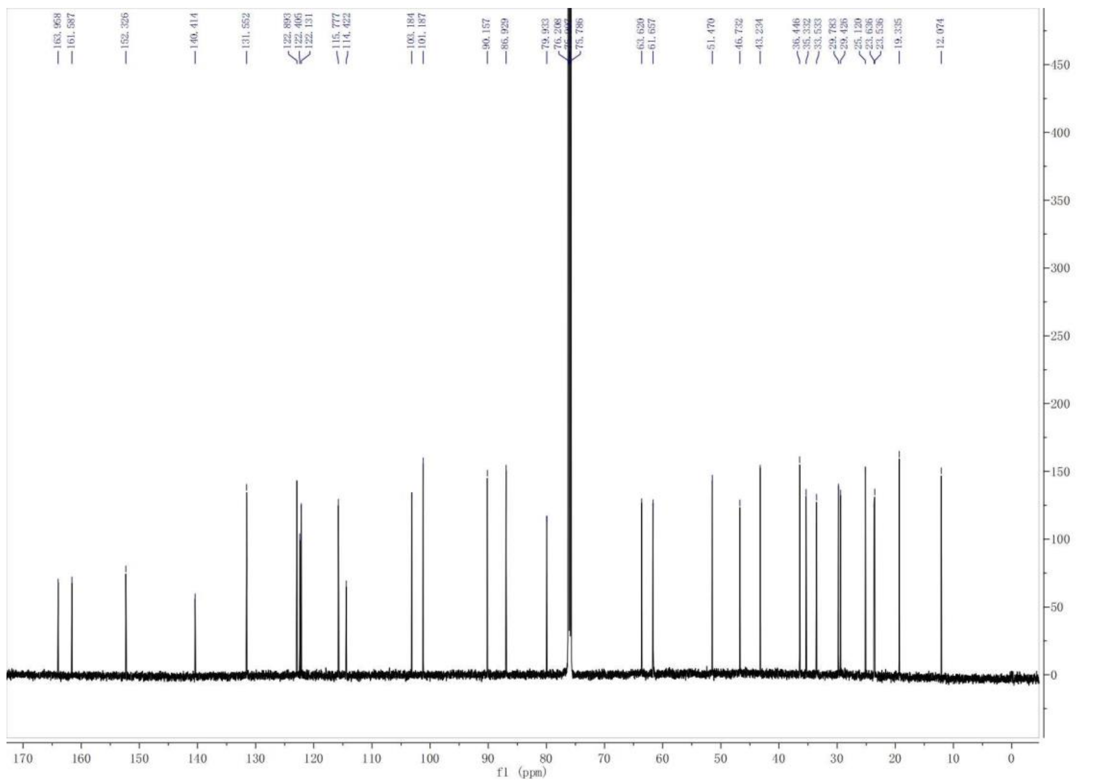

Compound **6k**

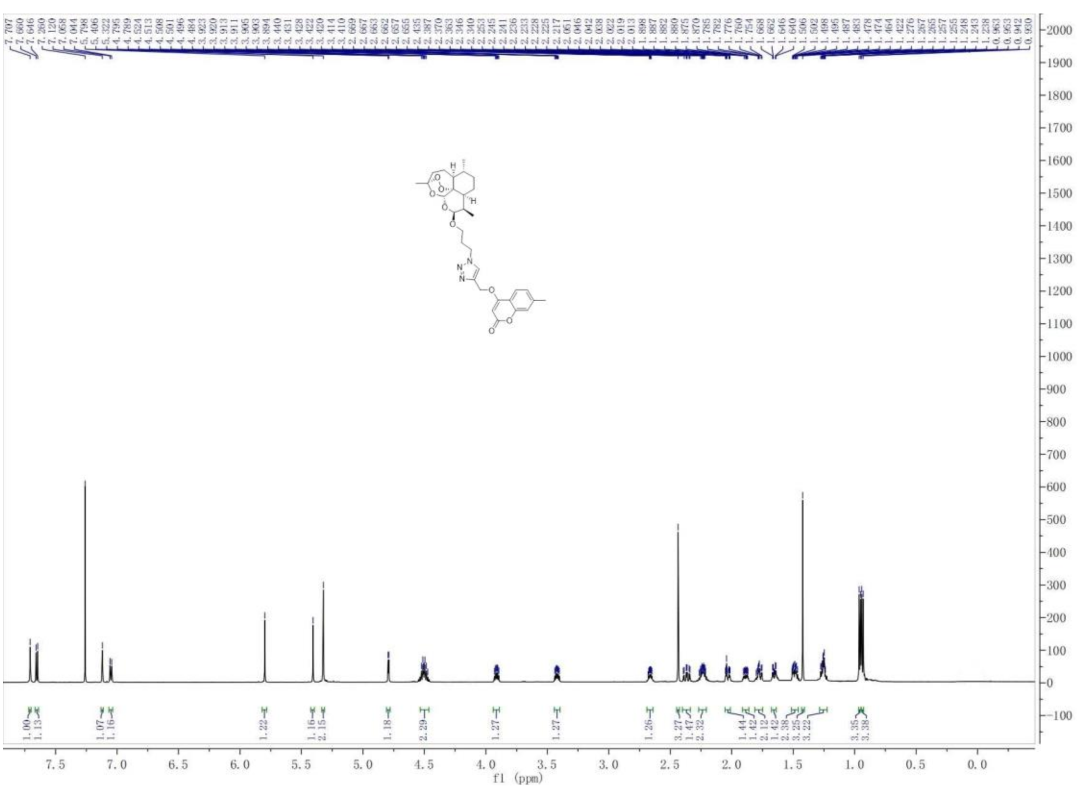

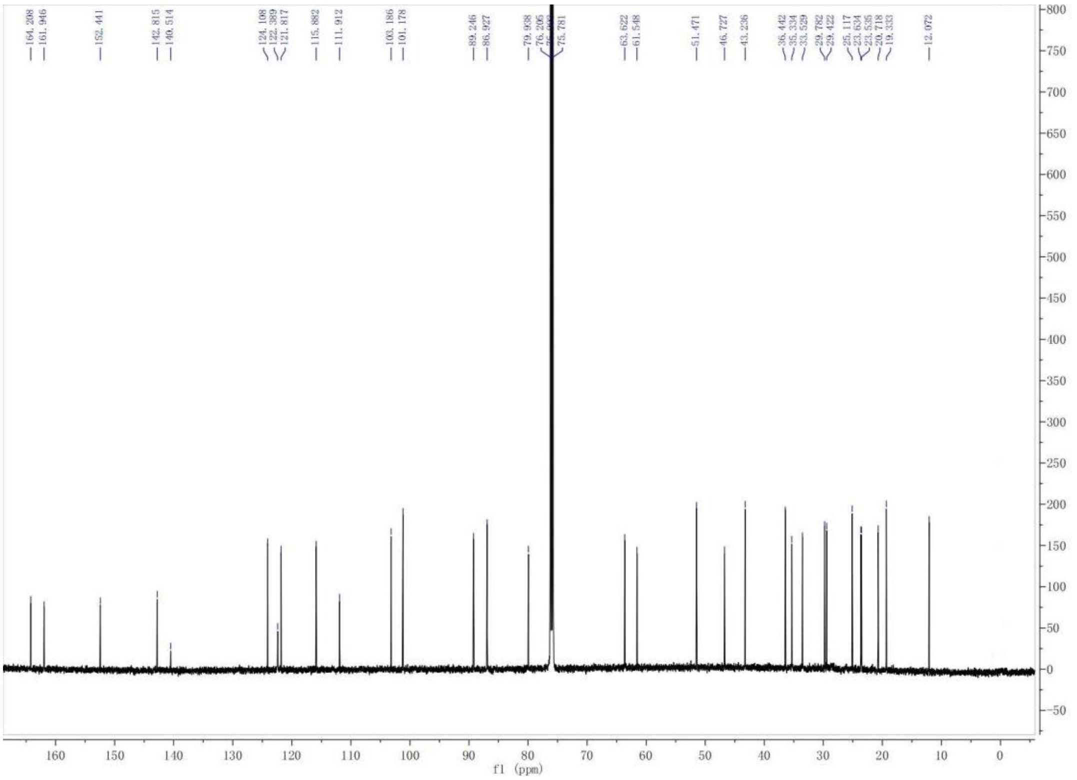

Compound **6l**

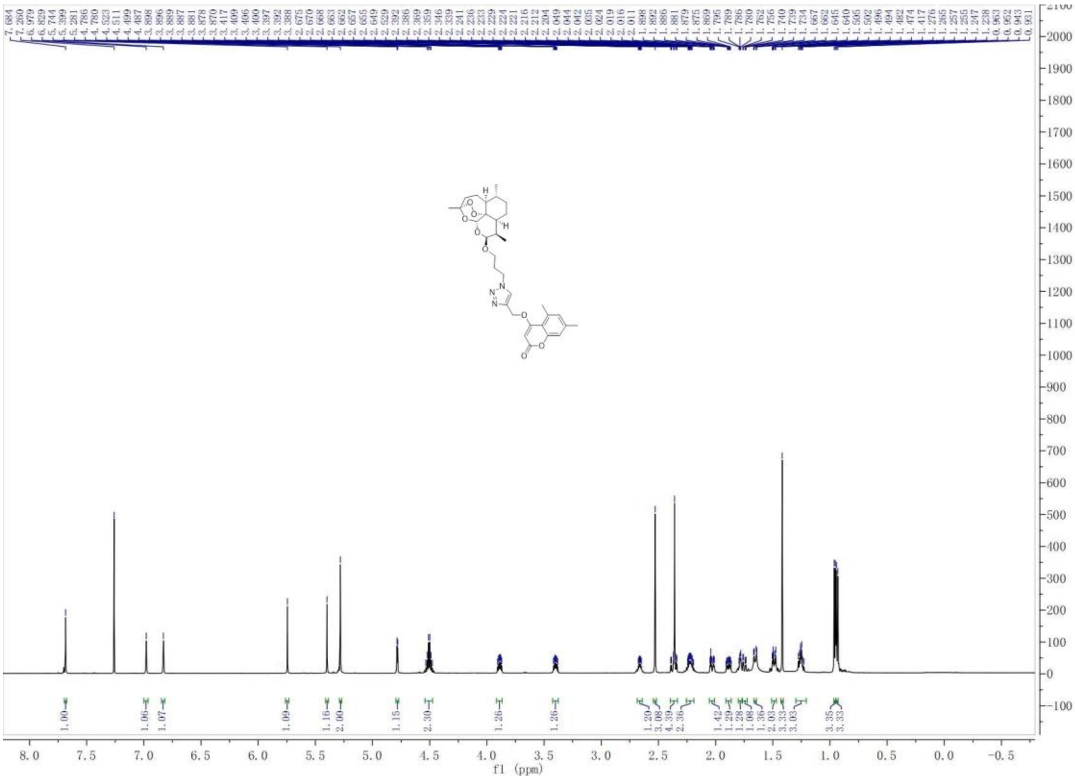

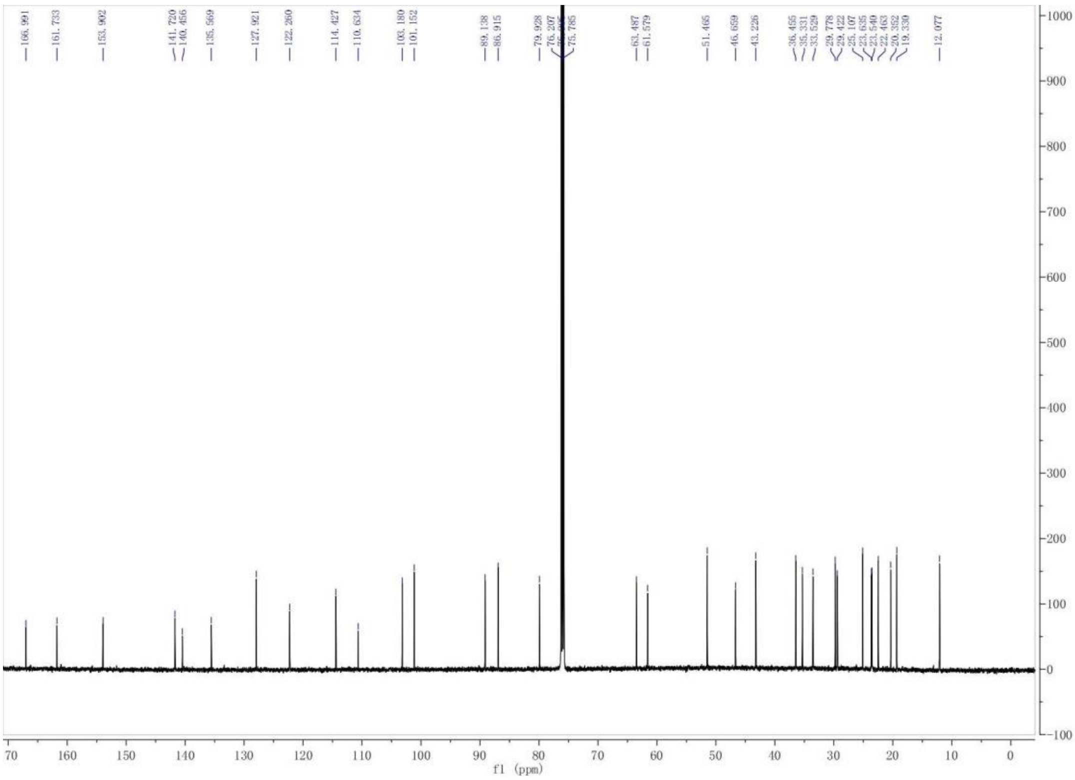

Compound **6m**

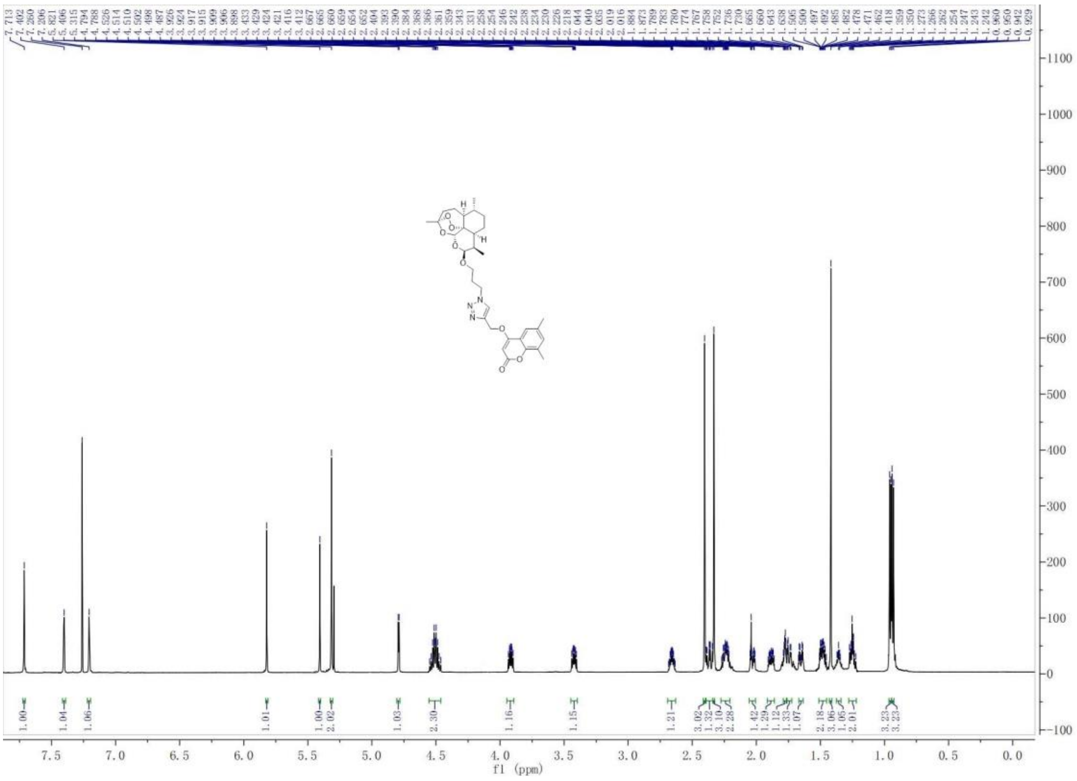

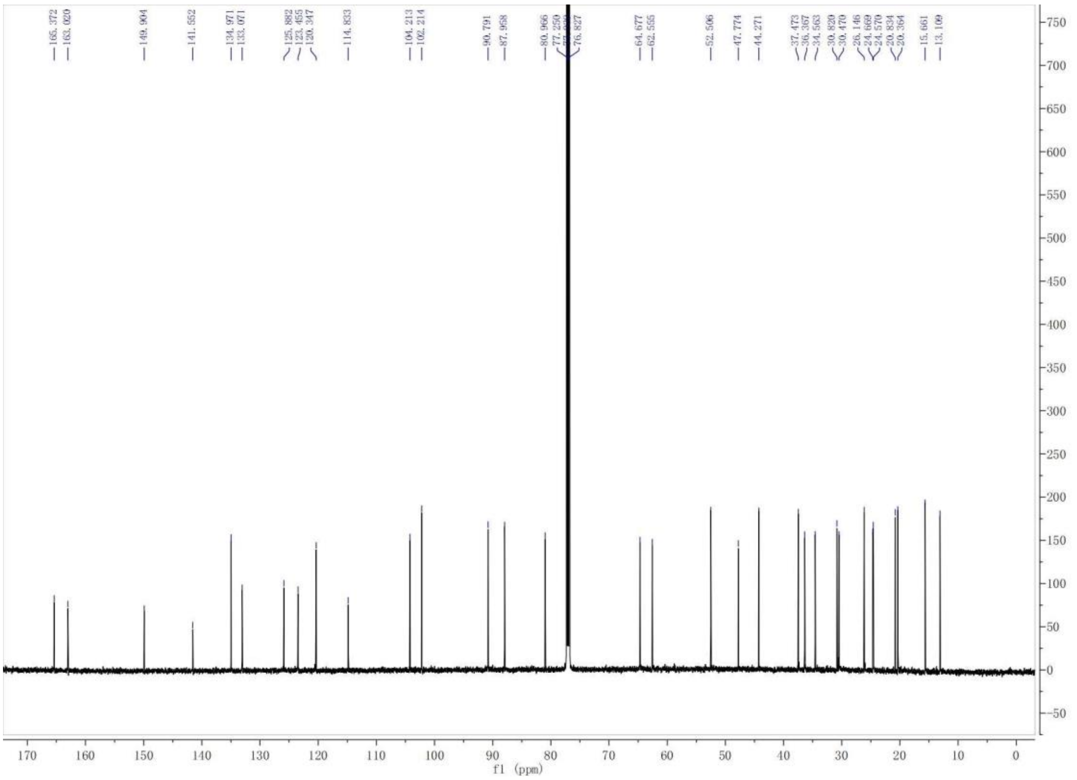

Compound **6n**

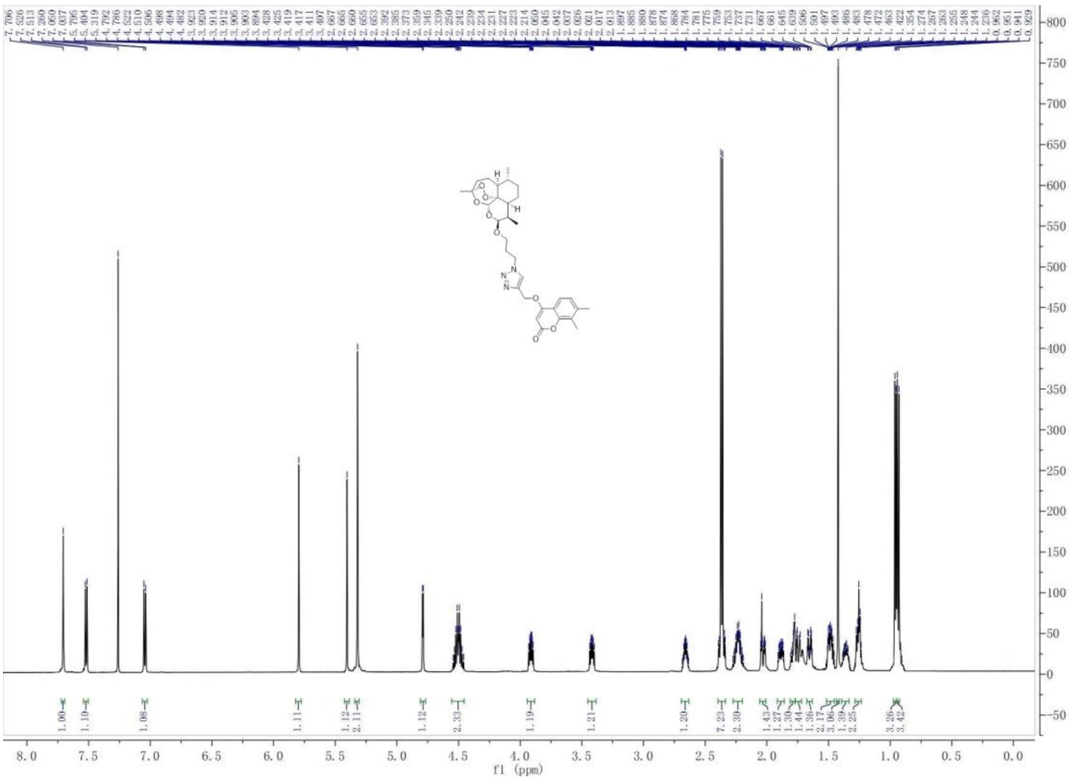
f1 (ppm)

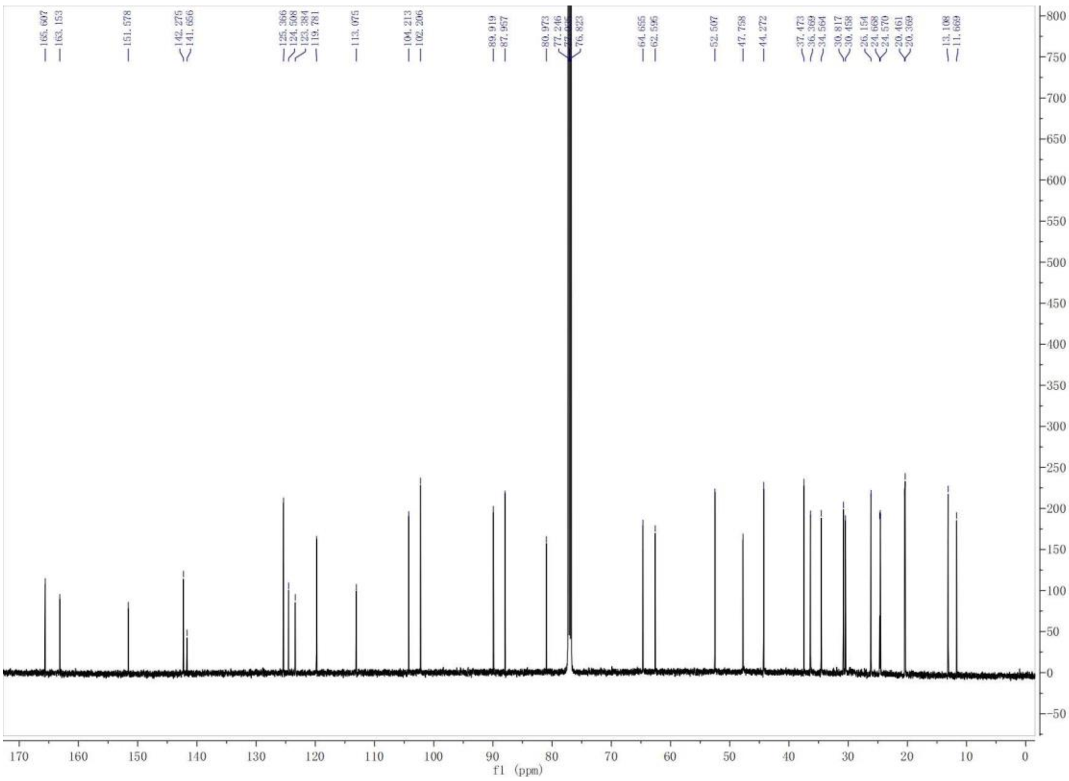
165.607
163.153
151.578
142.275
141.656
125.366
124.508
123.384
119.781
113.075
104.213
102.206
89.919
87.957
80.973
77.246
76.823
64.655
62.595
52.507
47.758
44.272
37.473
36.369
34.564
30.817
30.458
26.154
24.668
24.570
20.461
20.369
13.108
11.669
f1 (ppm)

Compound **6o**

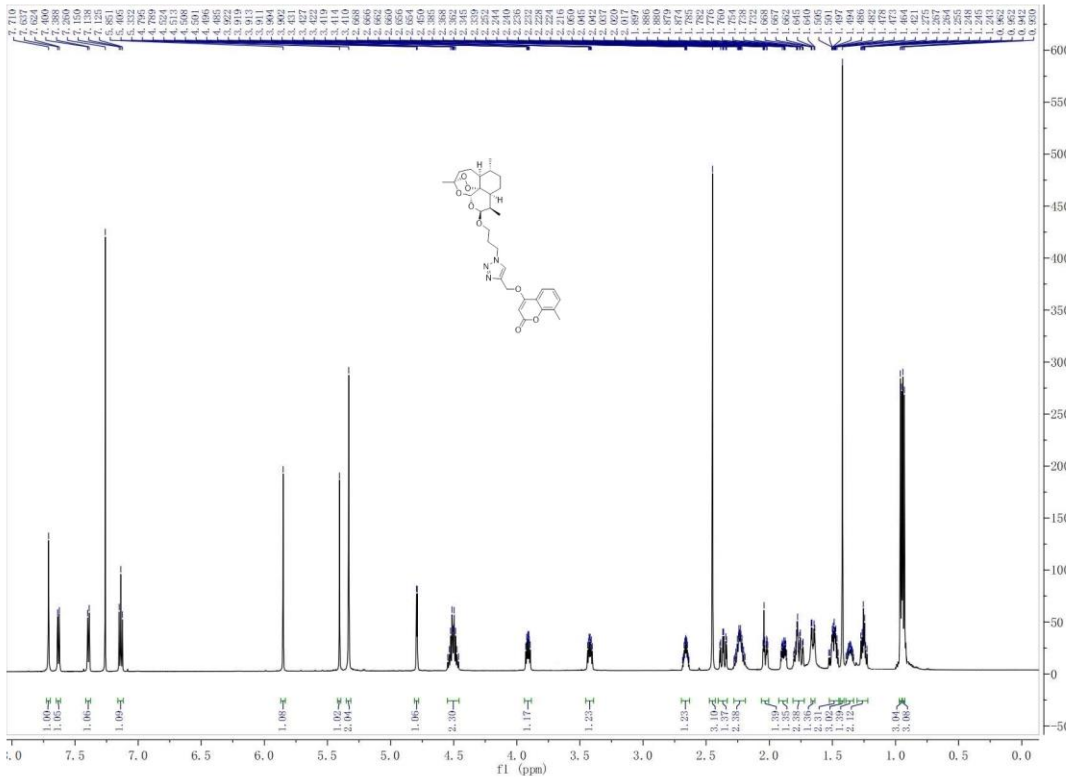

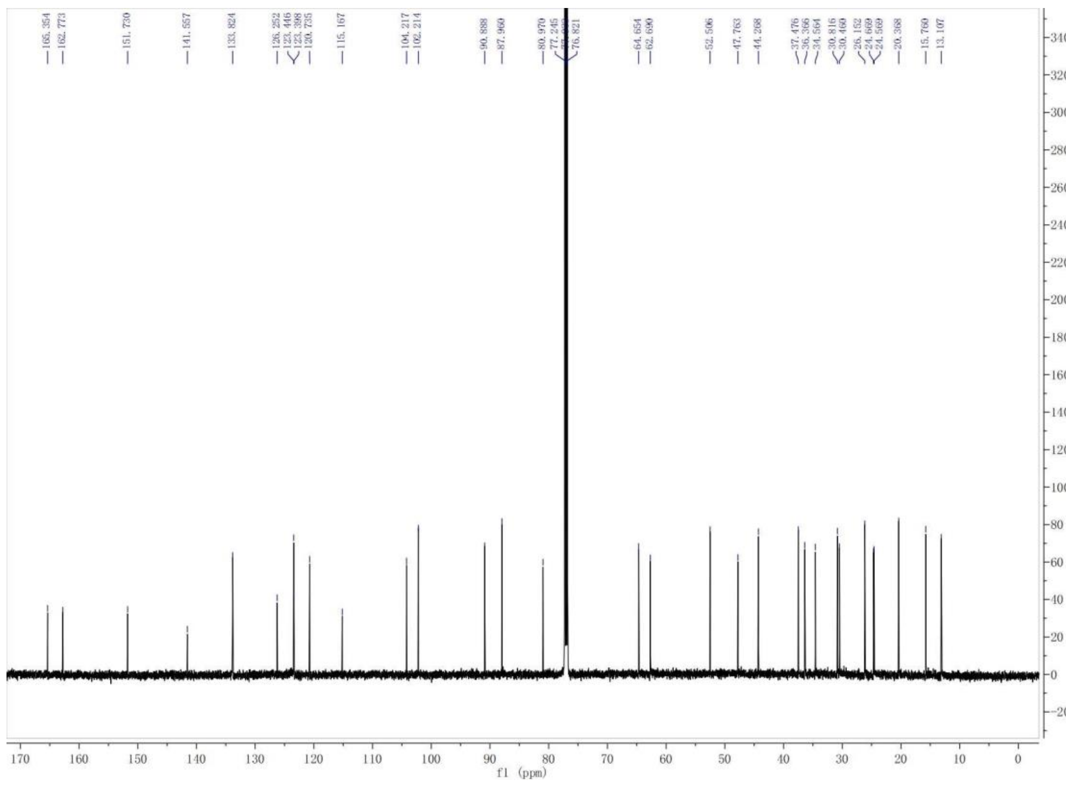

Compound **6p**

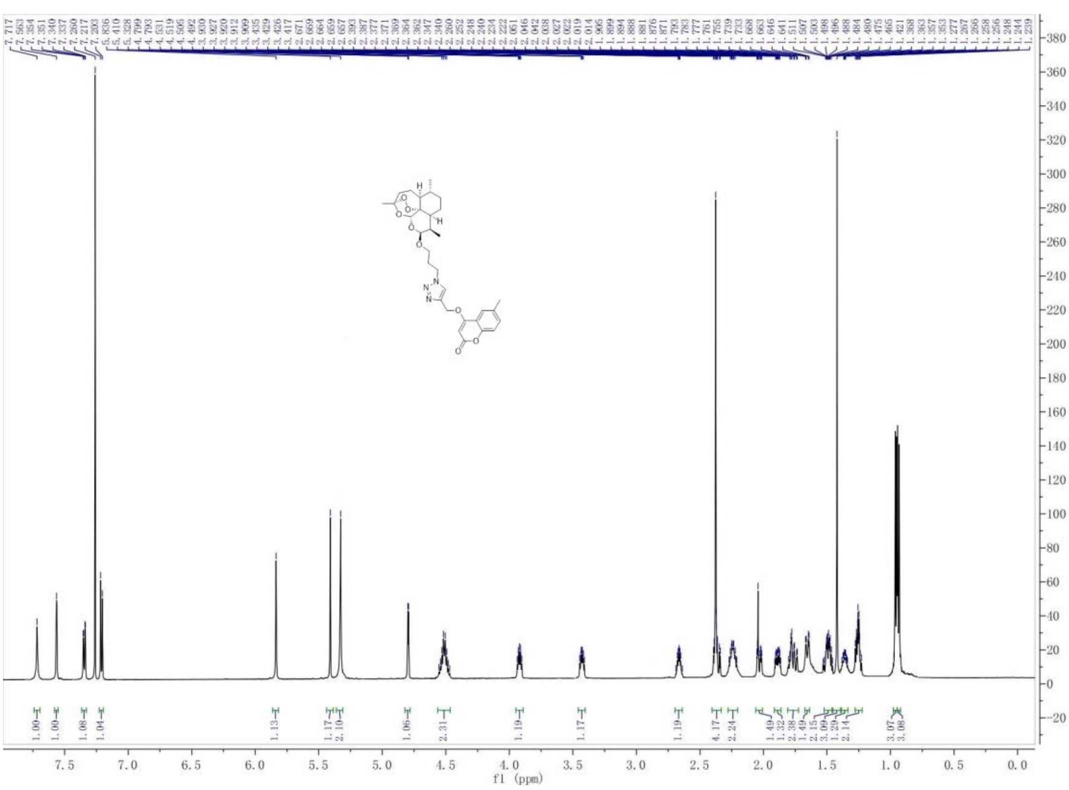

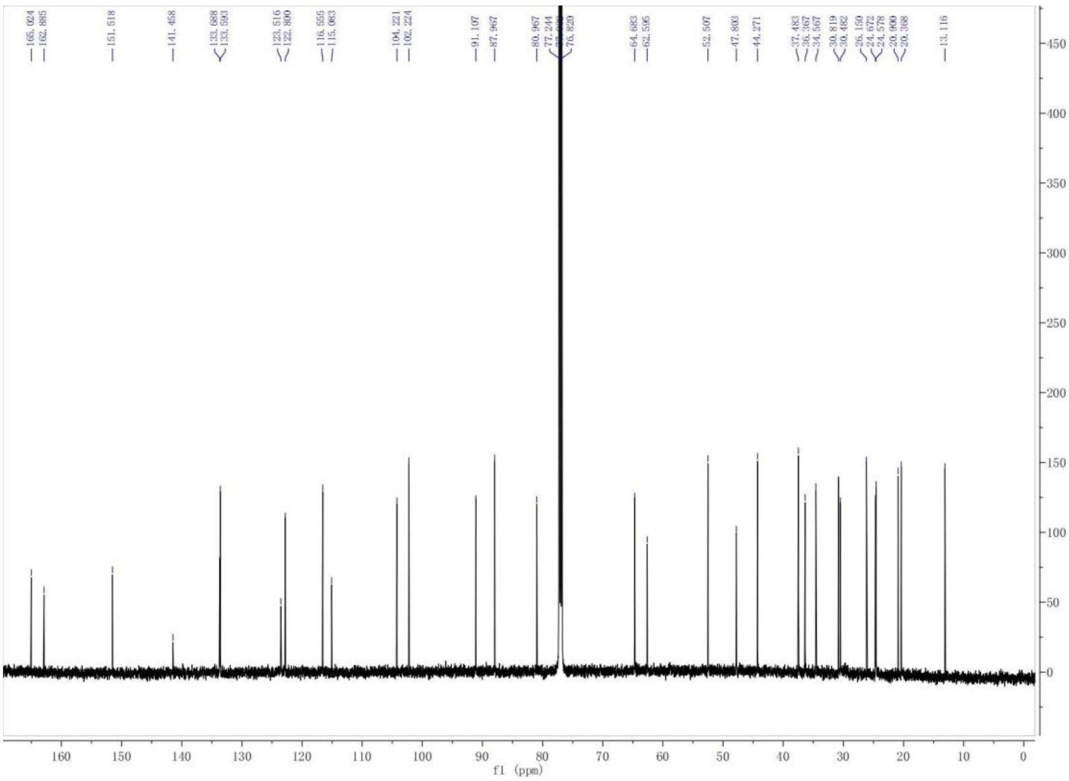

Supplement: Supplementary file 1 [file molecules-24-01672-s001.pdf]
